# Investigation of ion-electrode interactions of linear polyimides and alkali metal ions for next generation alternative-ion batteries†

**DOI:** 10.1039/d2sc02939a

**Published:** 2022-07-04

**Authors:** Cara N. Gannett, Jaehwan Kim, Dave Tirtariyadi, Phillip J. Milner, Héctor D. Abruña

**Affiliations:** Department of Chemistry and Chemical Biology, Cornell University Ithaca NY 14850 USA pjm347@cornell.edu

## Abstract

Organic electrode materials offer unique opportunities to utilize ion-electrode interactions to develop diverse, versatile, and high-performing secondary batteries, particularly for applications requiring high power densities. However, a lack of well-defined structure–property relationships for redox-active organic materials restricts the advancement of the field. Herein, we investigate a family of diimide-based polymer materials with several charge-compensating ions (Li^+^, Na^+^, K^+^) in order to systematically probe how redox-active moiety, ion, and polymer flexibility dictate their thermodynamic and kinetic properties. When favorable ion-electrode interactions are employed (*e.g.*, soft K^+^ anions with soft perylenediimide dianions), the resulting batteries demonstrate increased working potentials and improved cycling stabilities. Further, for all polymers examined herein, we demonstrate that K^+^ accesses the highest percentage of redox-active groups due to its small solvation shell/energy. Through crown ether experiments, cyclic voltammetry, and activation energy measurements, we provide insights into the charge compensation mechanisms of three different polymer structures and rationalize these findings in terms of the differing degrees of improvements observed when cycling with K^+^. Critically, we find that the most flexible polymer enables access to the highest fraction of active sites due to the small activation energy barrier during charge/discharge. These results suggest that improved capacities may be accessible by employing more flexible structures. Overall, our in-depth structure–activity investigation demonstrates how variables such as polymer structure and cation can be used to optimize battery performance and enable the realization of novel battery chemistries.

## Abstract 2

Organic electrode materials offer unique opportunities to utilize ion-electrode interactions to develop diverse, versatile, and high-performing secondary batteries, particularly for applications requiring high power densities.

## Graphical abstract

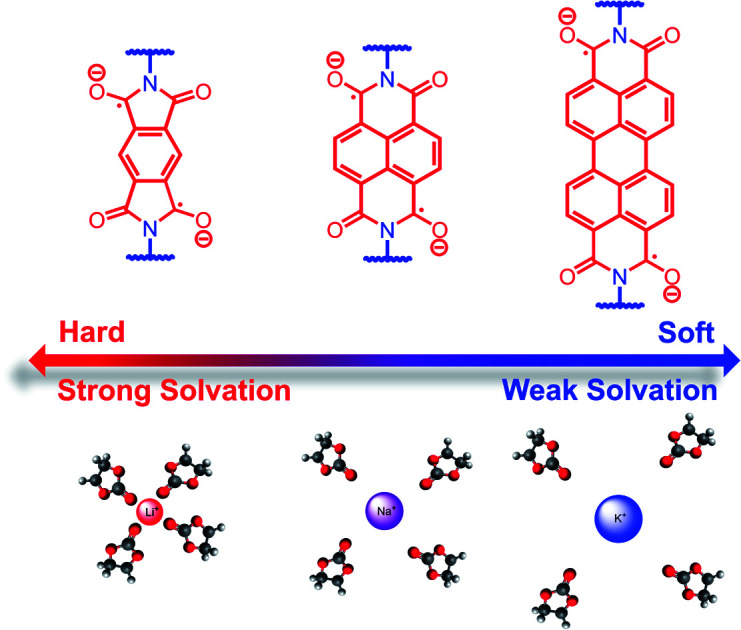

## Introduction

As anthropogenic activity increases, the global demand for energy will also continue to grow.^1^ Currently, the dominant methods of generating energy (*e.g.*, combustion of fossil fuels) are the leading sources of greenhouse gas emissions.^2,3^ Thus, it is imperative that the world quickly transition to renewable energy to avoid irreversible consequences to the planet. However, the intermittent nature of renewable energy sources requires reliable energy storage systems that satisfy the requirements of a sustainable global economy.^4^

Lithium-ion batteries (LIBs) are considered the best-in-class energy-storage technologies due to their high energy densities, long cycle lifetimes, and safety.^5^ However, LIBs do not meet all of the requirements of versatile and sustainable energy storage technologies. For example, modern LIBs utilize inorganic cathode materials that are energy-intensive to produce due to the associated extraction, refinement, and synthesis procedures.^5–8^ Moreover, Li^+^ salts also have high extraction and refinement costs, and it is unknown if salt production can match future demand.^9,10^ As such, it is desirable to implement alternative ions, such as Na^+^ and K^+^, into secondary battery technologies.^11,12^ However, the rigid and dense structures of modern LIB electrode materials cannot be readily adapted to work with cations other than Li^+^.^7,8,13–15^ Alternative-ion battery research has therefore focused on developing inorganic electrode materials with open framework structures or those involving conversion reactions.^16^ Although both approaches show promise, the restrictions of using inorganic cathodes mandates design of a specific electrode material for a specific alternative-ion battery application, limiting their generalizability.

Organic electrode materials have generated increased interest due to their abundant constituents, tunable and flexible structures, and high theoretical specific capacities.^7,8,13–15,17^ These qualities make organic electroactive materials sustainable, versatile, and powerful candidates for future battery applications.^18^ Further, many organic electrode materials are compatible with a wide range of ions.^19–24^ This enables a given material to be studied with different charge-compensating ions in order to systematically investigate how the cation affects battery performance. Recent interest in examining organic electrode materials for alternative-ion batteries has surged, particularly for their use in sodium-ion batteries. However, to date, there are only a handful of studies aimed at systematically understanding how alternative ions perform with a particular material, or family of related materials, in order to elucidate useful structure–property trends that can be generalized to enable future battery development.^13,15,25–30^

**Fig. 1:**
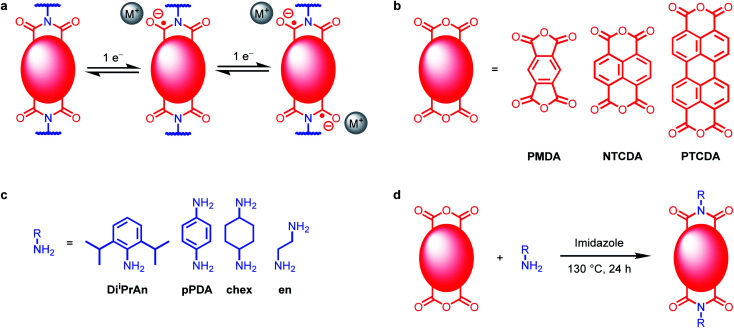
(a) Two one-electron reductions of diimides. (b) Tetracarboxylic acid dianhydrides used in this work. (c) Amines used in this work. (d) Synthesis of polymeric diimides.

Herein, we examine a family of n-type diimide organic electrode materials for their battery performance when paired with different charge-compensating ions (Fig. 1). The common arylene diimide functional group shared by all investigated materials enables us to examine the interactions of ions with redox-active moieties with differing abilities to delocalize electrons within their aromatic structures. We demonstrate that hard-soft acid base theory enables predictions about the interaction strength between cation and reduced polymer, allowing us to predict that, for example, soft K^+^ cations perform best when paired with soft perylene diimide (PDI) units. In addition, by investigating several closely related polymers containing PDI units, we reveal that charge compensation mechanism and battery performance are highly dependent on both the polymer structure and the solvation energy of the charge-compensating ions. In contrast to the movement of the field towards rigid crystalline materials such as covalent organic frameworks (COFs),^31^ we demonstrate the limiting processes of ion insertion can be minimized in flexible polymers, leading to the highest capacities and rate capabilities reported in this work. These findings point to the importance of systematic studies for uncovering properties of organic electrode materials that can be further optimized for next-generation alternative-ion batteries.

## Results and discussion

### Effect of carbonyl-ion interactions on redox potentials

We set out to understand the role of electrode-ion interactions on the electrochemical performance of battery systems. To effectively examine these interactions, we varied both the redox-active moiety and the charge-compensating ion in both molecular and polymeric systems. A standardized redox-active functional group, namely, arylene diimide (Fig. 1a), was chosen for this investigation due to the promising performance of arylene diimides as organic electrode materials.^30,32,33^ The size of the redox-active unit can be varied by changing the aromatic anhydride used during the synthesis: pyromellitic dianhydride (PMDA) to prepare pyromellitic diimides (PMDIs), 1,4,5,8-naphthalene tetracarboxylic dianhydride (NTCDA) to prepared naphthalene diimides (NDIs), or 3,4,9,10-perylene tetracarboxylic dianhydride (PTCDA) to produce PDIs (Fig. 1b; see ESI Section 2† for details). Although PDI-based materials are generally considered challenging to prepare due to the poor solubility of PTCDA, we have found that they can generally be synthesized from PTCDA and (poly)amines using molten imidazole as the reaction solvent.^34^

**Fig. 2:**
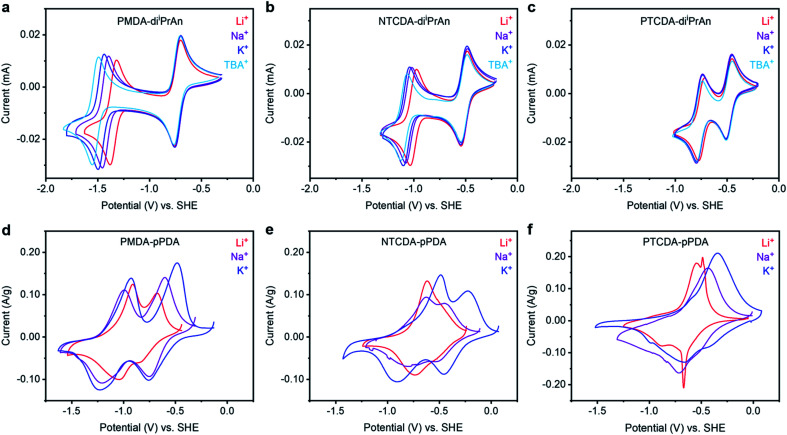
Cyclic voltammograms at 50 mV s^−1^ of solutions containing (a) PMDA-di^i^PrAn, (b) NTCDA-di^i^PrAn, and (c) PTCDA-di^i^PrAn in DMF in the presence of LiClO_4_, NaClO_4_, KClO_4_, or TBAP (0.1 M). Cyclic voltammograms at 0.25 mV s^−1^ of (d) PMDA-pPDA, (e) NTCDA-pPDA, and (f) PTCDA-pPDA in metal (Li, Na, or K) half cells containing 1 : 1 EC : DEC (by vol) in the presence of LiPF_6_, NaPF_6_, or KPF_6_. The potential scales of the voltammograms were converted to potentials *vs.* SHE using tabulated values (see ESI† for conversion values).

In order to probe the effects of charge-compensating cations on electrochemical performance at the molecular level, PMDA-di^i^PrAn, NTCDA-di^i^PrAn, and PTCDA-di^i^PrAn were synthesized and characterized (Fig. 1c and d). The small molecules were dissolved in *N*,*N*-dimethylformamide (DMF) and examined by cyclic voltammetry (CV) in the presence of four perchlorate salts: LiClO_4_, NaClO_4_, KClO_4_, and (^*n*^Bu_4_N)ClO_4_ (TBAP). The resulting voltammograms are plotted in Fig. 2a–c. The voltage range was limited to the two-electron reduction of the molecules due to the irreversible decomposition that typically occurs when arylene diimides are further reduced.^28,35–37^ As such, two redox couples are observed in each voltammogram, corresponding to reduction to the anion and dianion, respectively.

The potential at which the first redox couple (0/−1) occurs for all three molecules does not significantly vary as a function of the charge-compensating ion. Notably, the formal potential (*E*^0^′) becomes more positive as the size of the aromatic unit increases (*E*^0^′(TBA^+^) for PMDI: −0.736 V → NDI: −0.517 V → PDI: −0.481 V), as the negative charge is more delocalized over the reduced diimide unit (ESI Fig. S88–S90†).^37,38^ Additionally, as the size of the conjugated unit increases, the difference in the potential of the first and second reduction events (Δ*E*) decreases (Δ*E*(TBA^+^) for PMDI: 0.786 V → NDI: 0.553 V → PDI: 0.288 V), resulting in a more stable battery working potential during discharge for the materials with larger aromatic systems. The potential difference arises from electrostatic repulsion; larger conjugated units provide a larger area to delocalize the negative charge and thus ameliorate the destabilizing repulsive effect.^39^

In contrast to the similar potential of the first redox couple across cations, the potential of the second redox couple of PMDA-di^i^PrAn exhibits a strong dependance on the charge-compensating ion (Fig. 2a). As the size of the charge-compensating ion decreases from TBA^+^ to Li^+^, the redox couple shifts anodically by over 170 mV, which corresponds to a 16.6 kJ mol^−1^ increase in the binding energy (ESI Table S13†). A positive shift in the reduction potential with higher charge density ions signals a higher binding energy between the reduced unit and the charge-compensating ion. This behavior is consistent with that of 1,2-diones, for which smaller, more densely charged ions bind more favorably to the reduced carbonyl units.^40^ However, this shift is less pronounced for NTCDA-di^i^PrAn and is not observed for PTCDA-di^i^PrAn (ESI Table S13†). This finding suggests that reduced NDI and PDI units are not stabilized to the same extent (or at all) by the interaction with more densely charged ions.

Polymeric materials incorporating arylene diimides were synthesized to investigate if the phenomena observed in solution-state studies extend to battery systems. The diamine *para*-phenylenediamine (pPDA) was reacted with PMDA, NTCDA, and PTCDA to form the linear diimide polymers PMDA-pPDA, NTCDA-pPDA and PTCDA-pPDA, respectively, by heating the monomers together in imidazole at 130 °C for 24 h. The high quality of the insoluble polymers was confirmed through characterization by ^1^H and cross-polarized (CP) ^13^C magic angle spinning (MAS) solid-state nuclear magnetic resonance (SSNMR) spectroscopies, attenuated total reflectance (ATR) Fourier transform infrared (FTIR) spectroscopy, UV-Vis spectroscopy, powder X-ray diffraction (PXRD), energy dispersive X-ray spectroscopy (EDS), and combustion analysis (see ESI Section 3† for details). In addition, thermogravimetric analysis (TGA) and differential scanning calorimetry (DSC) confirm the conversion of the monomers into the thermally stable polymers. In particular, all three polymers were confirmed to be free of the corresponding poorly soluble dianhydrides by PXRD, IR, and SSNMR. The successful synthesis of PTCDA-pPDA using this method presents an advantage over previous syntheses of PDI-based polymers that employ Lewis acids such as Zn(OAc)_2_,^41,42^ as it avoids unnecessary metal waste and minimizes potential decarboxylation of the anhydride units by Lewis acids during the polymerization process.^43^

After synthesis, the polymers were integrated into battery electrodes by mixing 60% active material, 30% Super P carbon, and 10% polyvinylidene fluoride (PVDF) binder by weight. The electrodes were assembled as cathodes into 2032 coin cells using Li, Na, or K metal as the anodes accompanied by the corresponding electrolyte solutions (LiPF_6_, NaPF_6_, or KPF_6_, respectively) in ethylene carbonate (EC):diethyl carbonate (DEC) (1 : 1 by vol). The EC : DEC mixture was chosen due to its wide electrochemical stability window and broad application in alkali-ion batteries, but a DOL : DME mixture was also preliminarily tested (ESI Fig. S76†). Furthermore, we aimed for electrolyte solutions to be as close to 1.0 M as possible, but KPF_6_ was only soluble up to 0.8 M. However, only small differences in performance were observed between 0.8 M and 1.0 M LiPF_6_ electrolyte solutions (ESI Fig. S75†). Solid-state CV experiments at 0.25 mV s^−1^ were performed to characterize the redox processes occurring as a function of arylene diimide unit and charge-compensating ion (Fig. 2d–f and ESI Fig. S54†). Again, notable trends were observed as a function of both the redox-active unit and the charge-compensating ion. As the size of the conjugated region within the redox-active moiety increased from PMDA-pPDA to NTCDA-pPDA to PTCDA-pPDA, the first reduction peak potential increased (−0.758 V → −0.455 V → −0.404 V *vs.* SHE for K cells), consistent with observations from the small molecule analogues (ESI Fig. S55–S56†). Additionally, as the size of the conjugated region increases for cells with Na^+^ and K^+^, the difference in the potential of the first and second reduction events decreases (ESI Fig. S56†), again consistent with the trends observed for the molecular analogues. However, this phenomenon was not observed in the presence of Li^+^.

Investigating the cation dependance of the reduction potentials for each polymer yields further insights into the ion-electrode interactions that were not observed in the solution-based voltammetric studies.^44^ The second reduction of PMDA-pPDA occurs at the most positive potentials when Li^+^ is present (−1.043 V) and the most negative potentials with K^+^ (−1.232 V), as observed with PMDA-di^i^PrAn. However, the opposite is true for PTCDA-pPDA, which is reduced to the dianion at the most negative potentials with Li^+^ (−0.883 V) and most positive potentials with K^+^ (−0.655 V). As the reduction potential represents the extent to which ion-electrode interactions are thermodynamically favored, these results suggest that reduced PDI^2−^ units are best stabilized by K^+^ ions, while reduced PMDI^2−^ units are best stabilized by Li^+^ ions.

Previous studies regarding similar potential shifts with certain ion-electrode pairings have attributed this behavior to hard–soft acid–base (HSAB) theory:^45–47^ a hard acid (cation) is expected to interact most favorably with a hard base (reduced carbonyl unit), while a soft acid should interact more favorably with a soft base.^48^ The extended conjugation in PTCDA-pPDA enables the negative charges to be delocalized over a large area, such that reduced PTCDA-pPDA acts as a soft base. In contrast, reduced PMDA-pPDA acts as a hard base. The larger size and lower charge density of K^+^ makes it a softer acid (chemical hardness *η* = 3.22) relative to Li^+^ (*η* = 3.80) and, as such, it should interact more favorably with a softer reduced species.^49^ Thus, when PTCDA-pPDA is paired with K^+^ (soft–soft), the most favorable interactions are observed in the form of an anodic shift in the reduction potential. Conversely, the opposite trend is observed when a hard base (PMDA-pPDA) is paired with a hard acid (Li^+^), and the largest anodic shift in the reduction potential of PMDA-pPDA was observed.

The NTCDA-pPDA polymer represents an intermediate case between PMDA-pPDA and PTCDA-pPDA, as NTCDA-pPDA shows both significant hard and soft base behavior depending on the extent of reduction. Specifically, the singly-reduced state acts as a somewhat soft base, interacting more favorably with Na^+^ or K^+^, and the dianionic state acts as a somewhat hard base, interacting more favorably with Li^+^. Therefore, the first reduction occurs at the most negative potential (−0.736 V) with Li^+^*vs.* with K^+^ (−0.463 V), while the second reduction the opposite trend is observed, as the reduction with K^+^ occurs at the most negative potential (−0.910 V) while the second reduction peak with Li^+^ merged with the first peak (−0.736 V).

It should be emphasized that these changes in potential arise solely due to different interactions between the cation and the redox-active unit. The chemical reaction, namely the reduction of the diimide units, remains the same across all three examined polymers. Nonetheless, very different reduction potentials are observed from the different interactions incurred by each ion-electrode pairing. By understanding and strategically utilizing these interactions, the formal potential and ensuing energy density of a battery material could be modified simply by tuning ion-electrode interactions.

### Effect of electrode-ion interactions on battery performance

**Fig. 3:**
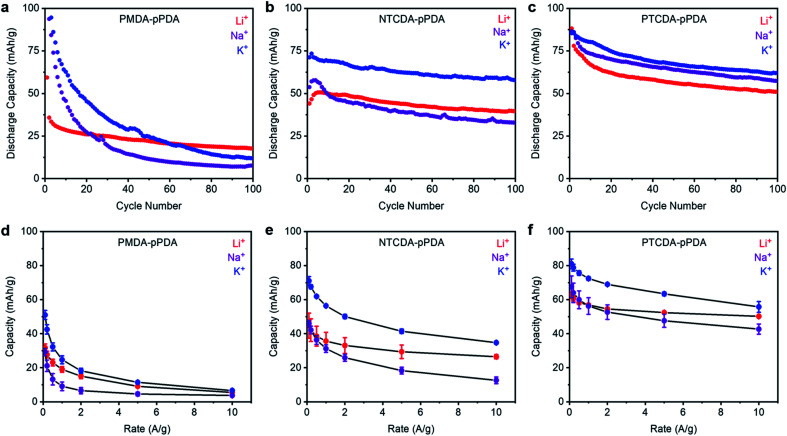
Cycling performance of (a) PMDA-pPDA, (b) NTCDA-pPDA, and (c) PTCDA-pPDA in metal (Li, Na, K) half cells at a discharge rate of 100 mA g^−1^. Average discharge capacities from rate tests for (d) PMDA-pPDA, (e) NTCDA-pPDA, and (f) PTCDA-pPDA in Li, Na, and K cells. The error bars represent one standard deviation of error in the measured capacity, determined from data obtained from at least three coin cells.

To evaluate how ion-electrode interactions affect battery performance, each combination of polymer and cation were first subjected to galvanostatic charge/discharge tests at 100 mA g^−1^ (ESI Fig. S58–S59†). The trends in the reduction potentials observed by CV are consistent with those observed in the voltage profiles from galvanostatic charge/discharge measurements. Next, each combination of polymer and cation was subjected to 100 cycles at a discharge rate of 100 mA g^−1^ (Fig. 3a–c). The Columbic efficiencies for all cells are included in ESI Fig. S57† and are close to 100% in all cases. For all materials examined here, the highest initial capacities were obtained in the cells containing K^+^. This implies that K^+^ is able to access more of the redox-active sites in each of the examined polymers. We hypothesize that this is due the smaller solvation shell and lower desolvation energy associated with the smaller charge density of K^+^.^50^ This may enable K^+^ to more easily diffuse into the electrode materials and access more of the arylene diimide sites than Na^+^ and Li^+^, which have larger solvation shells and higher desolvation energies.^19,51,52^ Further, PTCDA-pPDA exhibited the highest experimental capacity of the three materials studied here, despite its lower theoretical capacity (ESI Table S12†), which is consistent with previous studies.^53^

Upon cycling, PTCDA-pPDA exhibited the most stable performance in the presence of K^+^ (Fig. 3a–c, ESI Table S12†). Meanwhile, PMDA-pPDA exhibited the highest cycling stability when paired with Li^+^. To determine if the observed capacity decay for PTCDA-pPDA arises from degradation or from polymer dissolution, PTCDA-pPDA was cycled with Li^+^ using a solid polymer electrolyte (polyethylene oxide, PEO) instead of a liquid electrolyte solution (ESI Fig. S60†). Under these conditions, PTCDA-pPDA exhibited stable cycling capacity over 50 cycles, demonstrating that the observed capacity fade likely arises from polymer dissolution. Since the same solvent was utilized in all cells, we hypothesize that the difference in the ion-electrode interactions likely contributes to the differences in polymer solubilization upon cycling. Favorable interactions in the form of hard–hard or soft–soft pairings should provide stability to the reduced carbonyl units and prevent polymer dissolution. This could explain the more stable cycling performance of PTCDA-pPDA with K^+^ and PMDA-pPDA with Li^+^. Material dissolution is a common obstacle for organic electrode materials.^54,55^ Utilizing favorable ion-electrode interactions could provide an alternative way to combat this challenge without adding mass or altering the structure of organic electrode materials.

The performances of the three polymers with Li^+^, Na^+^, and K^+^ at different discharge rates were investigated to examine how cation–electrode interactions affect capacity retention at fast discharge rates (Fig. 3d–f, see ESI Fig. S61–S62† for raw data). Among the studied materials, PTCDA-pPDA exhibited the best performance in terms of capacity and capacity retention with increasing discharge rate, followed by NTCDA-pPDA, and then PMDA-pPDA, as predicted previously.^37^ This indicates that more of the active sites in PTCDA-pPDA are accessible than in NTCDA-pPDA and PMDA-pPDA, which is likely due to improved electronic access and lowered repulsion between active sites in PTCDA-pPDA. Notably, PTCDA-pPDA retained 80% of its capacity when its discharge rate was increased from 0.1 A g^−1^ to 10 A g^−1^ using Li^+^, while PMDA-pPDA retained only 17% of its capacity. Interestingly, the K^+^ cells exhibit the highest capacity at faster rates of discharge in all samples (Fig. 3d–f). For instance, PTCDA-pPDA delivered 56 mA h g^−1^ in a K^+^ battery and 50 mA h g^−1^ in a Li^+^ battery when discharged at 10 A g^−1^, respectively. Thus, the lower solvation energy of K^+^ allows access to more redox-active sites and allows them to be more quickly accessed on the shorter time scales associated with faster charge/discharge rates.^19^

By examining the interactions of PMDI, NDI, and PDI polymers with three different charge-compensating ions (Li^+^, Na^+^, and K^+^), we demonstrate the significant impact of ion-electrode interactions on the thermodynamics and kinetics of the charge/discharge process. The observed trends are consistent with predictions from HSAB theory: by utilizing favorable interactions (hard–hard or soft–soft), the working potential and stability of the battery system increases. Further, for all polymers examined thus far, we observed the highest capacities in K^+^ batteries, demonstrating the promise of pairing K^+^ with organic electrode materials.

### Influence of PDI polymer structure on performance

Having demonstrated the importance of ion-electrode interactions on the thermodynamic properties of battery materials, we sought to better understand their influence on kinetic properties as well. Previous studies have demonstrated that the kinetics of organic battery systems are heavily influenced by the bulk structure of the redox-active material.^56,57^ Further, based on our findings above and previous work in the literature, we hypothesized that the kinetics should be influenced by the nature of the solvation shell associated with the charge-compensating ion as well.^19^ By varying both the structure of the electroactive materials and the charge-compensating ions, we aim to elucidate how these two parameters interact and ultimately dictate battery performance.

To understand how polymer features such as flexibility and crystallinity influence battery performance, we prepared several polymer materials from the highest performing redox-active moiety, PDI. By altering the structural ordering and chain flexibility of the organic electrode materials, the diffusion of the charge-compensating ions should be significantly altered as well.^56,58,59^ In this vein, two additional polymers, PTCDA-chex and PTCDA-en, were synthesized from *trans*-1,4-diaminocyclohexane (chex) and 1,2-ethylenediamine (en) and PTCDA to compare with PTCDA-pPDA (see ESI Section 3† for details). While PTCDA-chex is expected to share the rigidity and tight packing associated with PTCDA-pPDA, the non-planar cyclohexane rings should disrupt π–π stacking interactions. In contrast, PTCDA-en should be both less ordered and more flexible due to the linear alkyl linking units.^58^ Indeed, the ^1^H SSNMR resonance of the alkyl C–H protons in PTCDA-en is sharper than that of PTCDA-chex, reflecting their more dynamic and thus liquid-like environment (ESI Fig. S84†). It is worth noting that PTCDA-chex and PTCDA-en lack extended conjugation between the aromatic units and thus would likely conduct charge *via* charge hopping across PDI units. Preliminary calculations (ESI Fig. S85†) and previous studies on similar aromatic polyimide systems suggest that the optimized structure of PTCDA-pPDA is not completely planar either,^60^ making delocalization of charge across the fully sp^2^-hybridized polymer unlikely (see ESI Section 6†). Thus, it is expected that the charge transport mechanism is a hybrid of charge hopping across PDI units and limited delocalization of charge through the pPDA units in this material.

The PXRD patterns of all three PTCDA-based polymers indicate they are microcrystalline (ESI Fig. S82†). Among the three materials, PTCDA-pPDA possesses the greatest number of sharp reflections by PXRD, including several within the range of 2*θ* = 23–30° (corresponding to d-spacings between 3.0–3.8 Å).^61^ This suggests that PTCDA-pPDA exhibits a higher degree of ordered π–π stacking interactions relative to the other two polymers. Consistently, PTCDA-pPDA is the only polymer to exhibit significant N_2_ uptake at 77 K, with a Brunauer–Emmett–Teller (BET) surface area of 76 m^2^ g^−1^ (ESI Fig. S78–S79†). In contrast, PTCDA-en is only slightly microporous (BET surface area = 10 m^2^ g^−1^, ESI Fig. S80–S81†), and PTCDA-chex is nonporous to N_2_ at 77 K. Rigid polymers often exhibit enhanced surface areas compared to flexible polymers, although these results do not preclude PTCDA-pPDA from having densely packed regions that impede ion diffusion.^59,62^ Notably, no glass or melting transitions below 300 °C were observed for any of the materials by DSC (ESI Fig. S78†). This indicates that any ordering observed by PXRD likely results from local interactions between the aromatic systems and that the polymers do not possess significant long-range order.

**Fig. 4:**
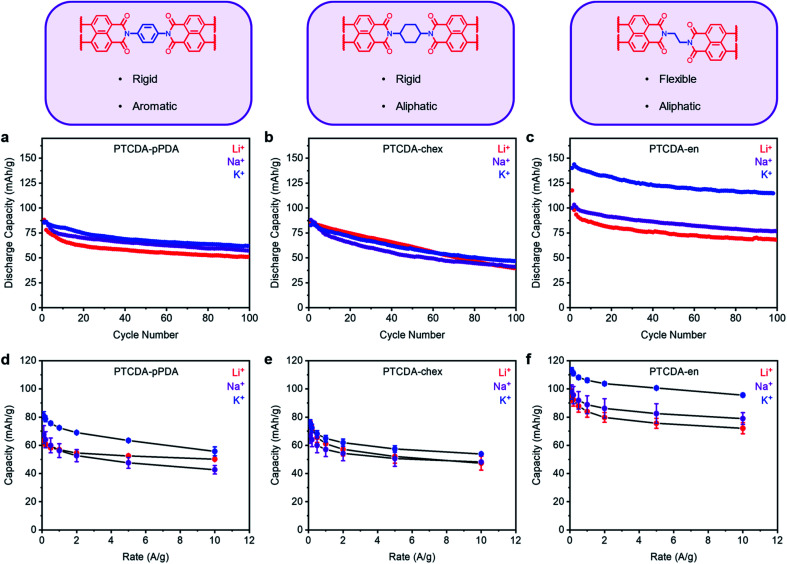
Structural descriptions and cycling performance of (a) PTCDA-pPDA, (b) PTCDA-chex, and (c) PTCDA-en in metal (Li, Na, K) half cells at 100 mA g^−1^. Average discharge capacities from rate tests for (d) PTCDA-pPDA, (e) PTCDA-chex, and (f) PTCDA-en in Li, Na, and K cells. The error bars represent one standard deviation of error in the measured capacity, determined from data obtained from at least three coin cells.

Following the procedure outlined above, PTCDA-en and PTCDA-chex were assembled into Li, Na, and K half cells and subjected to 100 cycles at a discharge rate of 100 mA g^−1^ (Fig. 4a–c). Among the tested combinations, PTCDA-en exhibited the highest capacity in the presence of K^+^, delivering an extra 45 mA h g^−1^ compared to the Li^+^ cell (Fig. 4c). This is greater than the improvement observed with PTCDA-pPDA, which delivered only 8 mA h g^−1^ more capacity with K^+^ compared to Li^+^ (Fig. 4a). Further, PTCDA-en with K^+^ delivers a maximum capacity of 143 mA h g^−1^, uniquely representing access to the full theoretical capacity among materials studied herein (*C*_theor_ = 128 mA h g^−1^, ESI Table S12†). The observed excess capacity is likely due to a combination of capacitive contributions from the carbon additives and solid electrolyte interface (SEI) formation during the initial cycles.^54,63^ Surprisingly, PTCDA-chex does not deliver more capacity in the presence of K^+^. Instead, the capacities and cycling performances with Li^+^, Na^+^, and K^+^ are nearly identical (Fig. 4c). Given that all the polymers investigated in these experiments have the same redox-active moiety, the discrepancies in performance with different charge-compensating ions indicate that factors beyond ion-electrode interactions play important roles in determining PDI battery performance.

The rate capabilities of the three PTCDA polymers were characterized and are summarized in Fig. 4d–f and ESI Fig. S66–S67 †. The most flexible polymer, PTCDA-en, delivered the best rate capabilities, consistent with previous reports regarding the improved rate performance of flexible polymers compared to their rigid counterparts.^59^ Further, PTCDA-en delivered the highest rate capabilities with K^+^: at 10 A g^−1^, the polymer delivered 96 mA h g^−1^ (84% retention), while with Li^+^, the capacity dropped to 72 mA h g^−1^ (76% retention). PTCDA-chex also delivered improved rate capabilities with K^+^ compared to Li^+^. Importantly, the higher observed rate capabilities of PTCDA-en compared to PTCDA-pPDA indicate that the introduction of aliphatic linkages through the polymer structure does not affect the intrinsic electrical conductivity of the polymer to an extent that limits battery performance.

### Effect of polymer structure on solvation dynamics

We hypothesized that solvation dynamics are confounded by the different polymer microstructures employed here. In addition to readily accommodating different charge-compensating ions, the flexible, spacious natures of redox-active polymers are known to accommodate ions with either no solvent molecules, a partial solvation shell, or even a full solvation shell.^56,64,65^ This contrasts with metal oxide electrode materials which require the charge-compensating ion to shed its solvent shell at the electrode–electrolyte interface prior to intercalation into the tightly packed lattice.^66^ The differences in performance as a function of ion in the three PDI-based polymers likely arise due to different phenomena associated with accommodating these ions within the polymer structures. To better probe the interactions responsible for the differing properties among polymer–ion combinations, crown ethers were added to the electrolyte solutions (12-crown-4 ether for Li^+^ batteries, 18-crown-6 ether for K^+^ batteries) to serve as strongly-coordinating and highly persistent chelating agents around the charge-compensating ions.^67–71^ In essence, crown ethers serve as a solvent shell that cannot be shed by the ion.

**Fig. 5:**
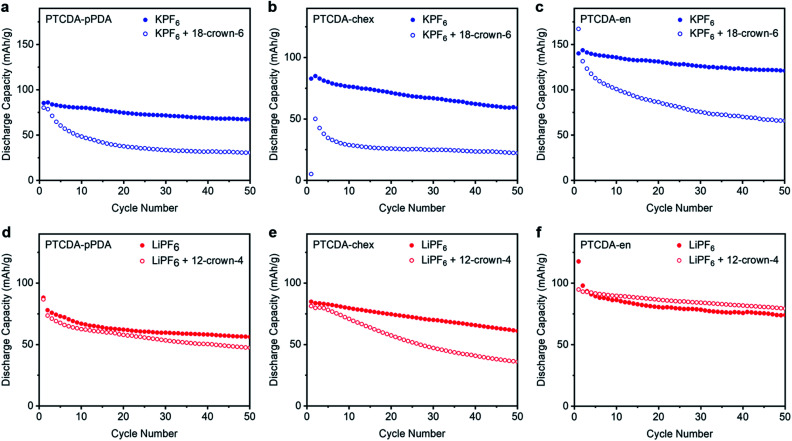
Cycling performance at 100 mA g^−1^ for (a) PTCDA-pPDA, (b) PTCDA-chex, and (c) PTCDA-en in metal half cells containing 0.8 M KPF_6_ with and without 0.8 M 18-crown-6 ether and (d) PTCDA-pPDA, (e) PTCDA-chex, and (f) PTCDA-en in cells containing 1.0 M LiPF_6_ with and without 1.0 M 12-crown-4 ether.

The three PDI-based polymers were cycled in Li^+^ and K^+^ batteries with the respective crown ethers at 100 mA g^−1^; the results are plotted in Fig. 5. All potassium batteries cycled in the presence of 18-crown-6 (Fig. 5a–c) suffered from dramatic capacity loss, delivering capacities less than those in Li^+^ cells (ESI Fig. S68†). The increased capacities and improved cycling stabilities previously observed in K^+^ batteries were completely lost in the presence of the chelating agent. The capacity loss observed in the presence of strongly binding crown ether confirms that the improvements arising from K^+^ are a result of its low desolvation energy and/or small solvation shell. Additionally, we hypothesize that the crown ether may be preventing the stabilizing interactions previously observed between the PDI-based polymers and K^+^, resulting in the poor cycling stability observed in the crown ether experiments.

Unexpectedly, not all of the Li^+^ batteries experience capacity losses in the presence of 12-crown-4 (Fig. 5d–f). Instead, the performance of PTCDA-pPDA and PTCDA-en were not significantly impacted by the presence of crown ether. This suggests that the chelated Li^+^ can still access the same redox sites as the standard Li^+^ electrolyte, implying that Li^+^ does not fully shed its solvation shell before it is inserted into PTCDA-pPDA and PTCDA-en. Given the lower access of redox-active groups by Li^+^ compared to K^+^ (in the absence of crown ether), we hypothesize that solvated/partially solvated Li^+^ enters the structures of these polymers, and its larger effective radius prevents it from accessing the same fraction of redox-active groups that are accessible to K^+^. Contrarily, PTCDA-chex does deliver less capacity in the presence of 12-crown-4 ether upon cycling. We hypothesize that the rigid structure of PTCDA-chex may require the ions to be desolvated at the electrode–electrolyte interface. In the presence of crown ether, this desolvation process is energetically unfavorable. This finding is consistent with the completely non-porous structure of PTCDA-chex suggested by the 77 K N_2_ adsorption measurements, indicating there is likely minimal volume for diffusion of solvated ions through this material.

**Fig. 6:**
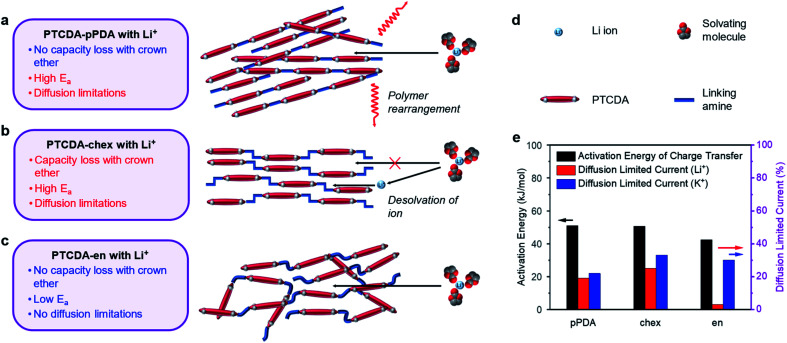
Schematics for the proposed charge compensation mechanisms for (a) PTCDA-pPDA, (b) PTCDA-chex, and (c) PTCDA-en in Li^+^ containing electrolyte solutions. The black arrows represent ion diffusion, and the red arrows represent polymer diffusion/rearrangement. (d) Legend corresponding to a–c. (e) Bar chart summarizing the activation energy of charge transfer with Li^+^ and diffusion-limited current proportion in Li^+^ and K^+^ batteries based on CV tests for the PDI-based polymers.

To further supplement this evidence, we measured the activation energies of the charge transfer processes in PTCDA-pPDA, PTCDA-chex, and PTCDA-en using potentiostatic electrochemical impedance spectroscopy (PEIS) at different temperatures (Fig. 6e, plots in ESI Fig. S71 and S72†). Among the polymeric materials, PTCDA-en exhibits the smallest activation energy (42.5 ± 1.0 kJ mol^−1^), indicative of a smaller kinetic barrier associated with the assimilation of Li^+^ into the structure of the polymer. Such a small activation energy may be expected if Li^+^ is incorporated into the polymer with the solvent shell still (at least partially) intact, as supported by the crown ether experiments with this combination of ion and polymer (Fig. 5f). Meanwhile, higher activation energies were measured for PTCDA-pPDA (51.1 ± 1.6 kJ mol^−1^) and PTCDA-chex (50.5 ± 0.4 kJ mol^−1^), implying larger kinetic barriers associated with the charge transfer processes in these materials. These findings indicate that energetic processes associated with charge/discharge such as the desolvation of ions or structural rearrangement of the polymers limit the performance of these materials compared to PTCDA-en.

To gain further insight into the processes dictating the electrochemical performance of PDI-based polymers, the charge storage kinetics of the polymers were evaluated by slow scan rate CV. Based on the relationships between current and scan rate, the portion of the current arising from diffusion-limited processes were determined (see ESI Section 4† for details). Diffusion limitations are known to arise from structural rearrangements, such as changes to the structure of the electrode material. We hypothesize that these limitations could also arise from changes in the structure of the solvation shell surrounding the charge-compensating ions. In the presence of Li^+^, PTCDA-chex exhibits the highest degree of diffusion-limited current at 25%, followed by PTCDA-pPDA at 19%, and PTCDA-en with only 2.5% (Fig. 6e, ESI Fig. S69†).

We attribute the high degree of surface-controlled kinetics in PTCDA-en to its flexible, amorphous structure which can accommodate charge-compensating ions without the need for significant structural rearrangement.^56,58^ The absence of diffusion limitations, low activation energy, and identical capacity delivered in the presence of crown ether likely indicates that the flexible structure of PTCDA-en enables Li^+^ to diffuse into the structure with its solvation shell intact (Fig. 6c). Interestingly, PTCDA-en exhibits a higher degree of diffusion limitations when paired with K^+^, where 30% of the current was found to be diffusion-limited (Fig. 6e and ESI Fig. S70†). Notably, the K^+^ battery can access 25% more of the redox-active material during charge/discharge tests. This suggests that the diffusion limitations are incurred when accessing the redox sites that are inaccessible to Li^+^. These diffusion limitations could arise from the structural rearrangement of the solvent molecules surrounding K^+^, enabling the ions to access the remaining redox-active sites.^72,73^

In the case of PTCDA-pPDA, nearly identical capacities were observed in Li^+^ batteries with and without crown ether, likely indicating that Li^+^ enters the structure of the polymer without the removal of its solvation shell (Fig. 6a). The high activation energy and diffusion-limited charge storage could indicate that the polymer undergoes structural changes to accommodate ions and their solvent molecules during charge/discharge. This is further supported by the sharp peak observed in CV experiments, which is a typical signature of redox reactions coupled to structural rearrangements (ESI Fig. S69a†).^74^ The necessity of structural rearrangements in this material are likely due to its rigid, crystalline structure.

The addition of 12-crown-4 ether to Li^+^ batteries containing PTCDA-chex led to significant capacity loss. This finding, coupled with the high activation energy and diffusion-limited charge storage in this material, points to a mechanism in which Li^+^ sheds its solvent shell before diffusing into the polymer structure (Fig. 6b), reducing the effective radius of the ion. Indeed, PTCDA-chex exhibits nearly identical capacity in the presence of Li^+^, Na^+^, and K^+^, unlike the other PDI-based polymers that exhibit decreased capacities when coupled with Li^+^. Owing to the smaller effective radius of the desolvated Li^+^, it can now access the same number of redox-active groups as the other two desolvated ions.

The different responses recorded in the experiments reported herein point to different charge compensation mechanisms as a function of both ion and polymer structure. The responses and different proposed charge compensation mechanisms are summarized in Fig. 6.

## Conclusions

The dominance of inorganic materials as battery electrodes has resulted in the consideration of alternative-ion batteries as distinct technologies, instead of as another handle for tuning battery performance. Organic electrode materials allow for the simple implementation of alternative ions within their more flexible, spacious structures. Herein, we reveal several aspects of polymer battery chemistry that demonstrate how alternative ions can be rationally employed to improve the performance of organic electrode materials.

Through the rigorous examination of five polymers (PMDA-pPDA, NTCDA-pPDA, PTCDA-pPDA, PTCDA-chex and PTCDA-en) and three ions (Li^+^, Na^+^, and K^+^), we have identified several interactions that can be used to tune both thermodynamic and kinetic aspects of battery performance. By changing the interactions between cations and reduced redox-active species, the potential and cycling stability of the redox couples can be modulated following the principles of HSAB theory. In addition, differences in the solvation structures of the examined ions—and the need or lack thereof for the ion to shed its solvent shell to reach redox-active units—were found to heavily influence battery performance. These effects can be further exaggerated by polymer structure. The mechanism by which ions diffuse into an active material is determined by the structural traits of a polymer including structural ordering, flexibility, and packing mode. Further, the nature of the solvation of the charge-compensating ion affects the energetics associated with these different incorporation pathways.

In designing an electrode material with high accessible capacity at fast discharge rates, the activation barrier associated with charge storage must be minimized. In this work, we demonstrate that this can be best accomplished with a flexible polymer and ions of lower solvation energies (*e.g.*, K^+^). Future work will expand upon these findings by investigating polymer networks that allow better control over other structural features including dimensionality, crystallinity, and porosity.

## Data availability

Experimental data is available upon request to the corresponding authors.

## Author contributions

C. N. G., J. K., P. J. M., and H. D. A. conceived the project. J. K. synthesized and characterized all molecules and polymers. C. N. G. carried out all electrochemical measurements with assistance from D. T. The final manuscript was written through the contributions of all authors, and all authors approved of the final version.

## Conflicts of interest

There are no conflicts to declare.

## Supplementary Material

SC-013-D2SC02939A-s001
