# Supplementary material for: Investigation of ion-electrode interactions of linear polyimides and alkali metal ions for next generation alternative-ion batteries
Source: Chem Sci. 2022 Jul 4;13(32):9191–201. doi: 10.1039/d2sc02939a (PMC9384138; doi:10.1039/d2sc02939a)
Supplement: SC-013-D2SC02939A-s001 [file SC-013-D2SC02939A-s001.pdf]

Electronic Supplementary Material (ESI) for Chemical Science.
This journal is © The Royal Society of Chemistry 2022

Investigation of Ion-Electrode Interactions of Linear Polyimides and Alkali Metal Ions for Next Generation Alternative-Ion Batteries

Cara N. Gannett,[a, ‡] Jaehwan Kim,[a, ‡] Dave Tirtariyadi,[a] Phillip J. Milner,[a,*] Héctor D. Abruña[a,**]

[a]Department of Chemistry and Chemical Biology, Cornell University, Ithaca, NY, 14850, United States
*pjm347@cornell.edu
**hda1@cornell.edu
‡These authors contributed equally.

Supporting Information

Table of Content

1. General reagents and procedures. 3
2. Synthesis and characterization of molecular analogs. 5
   a. Synthesis of *N*,*N*'-bis(2,6-diisopropylphenyl)-pyromellitic diimide (PMDA-di^{i}PrAn)....... 5
   b. Synthesis of *N*,*N*'-bis(2,6-diisopropylphenyl)-1,4,5,8-naphthalenetetracarboxylic diimide (NTCDA-di^{i}PrAn). 7
   c. Synthesis of *N*,*N*'-bis(2,6-diisopropylphenyl)-3,4,9,10-perylenetetracarboxylic diimide (PTCDA-di^{i}PrAn). 9
3. Polymer synthesis and characterization. 11
   a. Synthesis and characterization of PMDA-pPDA from pyromellitic dianhydride (PMDA) and *para*-phenylene diamine (pPDA). 12
   b. Synthesis and characterization of NTCDA-pPDA from 1,4,5,8-naphthalene tetracarboxylic dianhydride (NTCDA) and *para*-phenylene diamine (pPDA). 17
   c. Synthesis and characterization of PTCDA-pPDA from 3,4,9,10-perylene tetracarboxylic dianhydride (PTCDA) and *para*-phenylene diamine (pPDA) 22
   d. Synthesis and characterization of PTCDA-chex from 3,4,9,10-perylene tetracarboxylic dianhydride (PTCDA) and 1,4-diaminocyclohexane (chex). 28
   e. Synthesis and characterization of PTCDA-en from 3,4,9,10-perylene tetracarboxylic dianhydride (PTCDA) and 1,2-ethylenediamine (en). 34
4. Electrochemical experiments. 40
   a. Solution CV/RDE experiments. 40
   b. Coin cell fabrication. 40
   c. Cyclic voltammetry studies to determine diffusion-limitations. 41
   d. Activation energy measurements. 41
   e. Conductivity measurements. 42
   f. Supporting figures. 43

g. Additional electrochemical experiments with changed parameters and solubility tests.... 53
5. Structural comparison and surface area measurements for PTCDA-based polymers. 56
6. Computational structural models. 60
a. PTCDA-based polymers. 60
b. Molecular analogues made with di^{i}PrAn. 62
7. Tabulated calculated data for polymers and molecular analogues. 63
8. References. 65

1. General reagents and procedures.

All reagents and materials, including imidazole (99%, Fluka), 1,4,5,8-naphthalene tetracarboxylic dianhydride (**NTCDA**, 97%, Alfa Aesar), 3,4,9,10-perylene tetracarboxylic dianhydride (**PTCDA**, 95%, Oakwood), pyromellitic dianhydride (**PMDA**, 98%, TCI), *para*-phenylene diamine (**pPDA**, 98%, VWR), *trans*-1,4-diaminocyclohexane (**chex**, 98%, TCI), 1,2-ethylenediamine (**en**, 99%, Acros), 2,6-diisopropylaniline (**di^{i}PrAn**, 90%, Fisher Scientific), hydrochloric acid (36.5–38.0%, J.T. Baker), acetone (99.5%, Petroleum and Solvents), acetic acid (AcOH, 99%, VWR), chloroform (99.8%, Fisher Scientific), dichloromethane (DCM, 99.5%, J.T. Baker), *N*,*N*-dimethylformamide (DMF, 99.8%, Fisher Scientific, dried over activated 4 Å molecular sieves before use), ethanol (EtOH, 95%, Fisher Scientific), ethyl acetate (99.5%, Fisher Scientific), hexanes (98.5%, Fisher Scientific), methanol (99.8%, Fisher Scientific), tetrahydrofuran (certified, Fisher Scientific), tetrabutylammonium perchlorate (Aldrich, recrystallized three times from ethyl acetate prior to use), potassium perchlorate (99%+, Aldrich), sodium perchlorate (98%+, Aldrich), lithium perchlorate (99.99%, Aldrich), 1-methyl-2-pyrrolidinone (NMP, anhydrous 99.5%, Sigma Aldrich), ethylene carbonate (EC, 99+%, Aldrich), diethyl carbonate (DEC, 99+%, Aldrich), anhydrous 1,3-dioxolane (DOL, 99.8%, Aldrich), anhydrous 1,2-dimethoxyethane (DME, 99.5%, Aldrich), lithium hexafluorophosphate (98+%, Aldrich), sodium hexafluorophosphate (98%, Aldrich), lithium bis(trifluoromethanesulfonyl)imide (99%, Aldrich), acetonitrile (MeCN, anhydrous, Fisher), polyethylene oxide (M_v ~600,000, Aldrich), lithium metal foil (0.75 mm, 99.9%, Alfa Aesar), sodium metal cubes (99.9%, Sigma-Aldrich), and potassium metal chunks (99%, Oakwood) were purchased from commercial vendors and used without further purification, unless otherwise specified.

Infrared (IR) spectra were collected on a Bruker Tensor II IR spectrometer with a diamond Attenuated Total Reflectance (ATR) attachment. Powder X-ray diffraction (PXRD) patterns were collected on a Rigaku Ultima IV diffractometer equipped with a Cu Kα source (λ = 1.5406 Å) and were baseline-corrected using OriginPro. Solution-state NMR data were collected on a Bruker INOVA 500 MHz spectrometer and are referenced to residual solvent. Magic angle spinning solid-state NMR (MAS SSNMR) ^{1}H and cross-polarized (CP) ^{13}C measurements were carried out using a Phoenix NMR HX NB Probe (3.2 mm) located within a Varian INOVA 500 MHz spectrometer. All MAS SSNMR experiments were carried out using samples packed within 35 µL rotors at a spinning speed of 20 kHz. Solid-state UV-Vis spectra were collected using a Shimadzu UV-2600i Spectrophotometer equipped with an ISR-2660 Plus Integration Sphere for solid-state measurements. Surface area data were collected on a Micromeritics 3-flex gas sorption analyzer using ultrapure N_2 (99.999%) and a liquid N_2 bath. Brunauer-Emmett-Teller (BET) and Langmuir surface areas were determined by linear least squares regression analysis using the linearized forms of the BET and Langmuir equations, respectively. Combustion elemental analysis was performed by Atlantic Microlab Inc. Thermogravimetric decomposition profiles were collected on a Q500 V6.7 thermogravimetric analyzer (TGA) using a temperature ramp of 5.00 °C/min from 40.00 °C to 600.00 °C under an atmosphere of zero grade air (20–22% O_2 in N_2). Differential scanning

calorimetry (DSC) measurements were carried out on a TA instruments DSC Auto 2500 differential scanning calorimeter using a heat-cool-heat procedure, with a heating temperature ramp of 10.00 °C/min from −50.00 °C to 320.00 °C and a cooling ramp of 5.00 °C/min from 320.00 °C to −50.00 °C.

Energy dispersive X-ray scattering (EDS) data were collected at 10.0 kV using a Zeiss Gemini 500 scanning electron microscope equipped with an Oxford Instruments Ultima Max 170 detector (detector type X-max) and processed using the AZtec software. To prepare samples for EDS, the powders were immobilized on carbon tape mounted on an aluminum stub. The samples were blown using compressed air to remove excess material not stuck to the tape and then were coated with a carbon layer.

2. Synthesis and characterization of molecular analogs.

a. Synthesis of *N,N'*-bis(2,6-diisopropylphenyl)-pyromellitic diimide (PMDA-di^{i}PrAn).

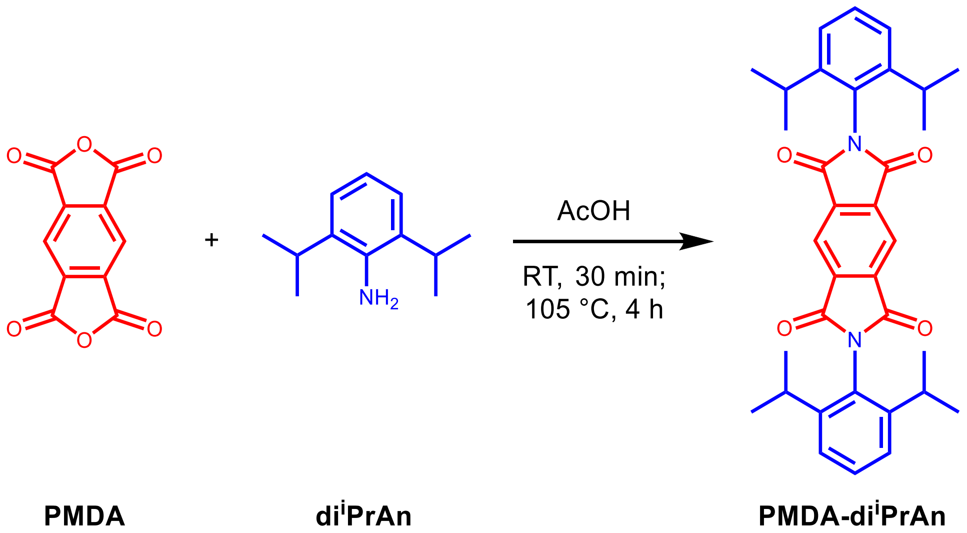

Figure S1. Synthesis of **PMDA-di^{i}PrAn**.

This procedure was adapted from the literature.[1] A 50 mL round-bottom flask equipped with a stir bar was charged with **PMDA** (1.00 g, 4.58 mmol, 1.00 equiv.), AcOH (20 mL), and **di^{i}PrAn** (2.59 mL, 13.8 mmol, 3.00 equiv.) in that order. The reaction mixture was allowed to stir for 30 min at room temperature. The reaction mixture was then headed to 105 °C and allowed to stir at 105 °C for 4 h with stirring. Additional AcOH (10 mL) was added when the reaction mixture reached 100 °C to facilitate mixing. A white solid precipitated from the reaction mixture after 90 min. The mixture was allowed to cool to room temperature and filtered. The resulting solid was rinsed with EtOH (50 mL) and deionized (DI) water (50 mL) and dried under vacuum to yield *N,N'*-bis(2,6-diisopropylphenyl)-pyromellitic diimide (**PMDA-di^{i}PrAn**) as an off-white solid (2.04 g, 83% yield). ^{1}H NMR (500 MHz, $CDCl_3$): δ 8.57 (s, 2H), 7.53 (t, J = 7.8 Hz, 2H), 7.35 (d, J = 7.8 Hz, 4H), 2.69 (sept, J = 6.8 Hz, 4H), 1.20 (d, J = 6.8 Hz, 24H) ppm; ^{13}C NMR (125 MHz, $CDCl_3$): δ 166.1, 147.0, 137.3, 130.7, 126.2, 124.2, 119.7, 77.2, 29.6, 24.0 ppm. These spectra are consistent with those reported in the literature.[1]

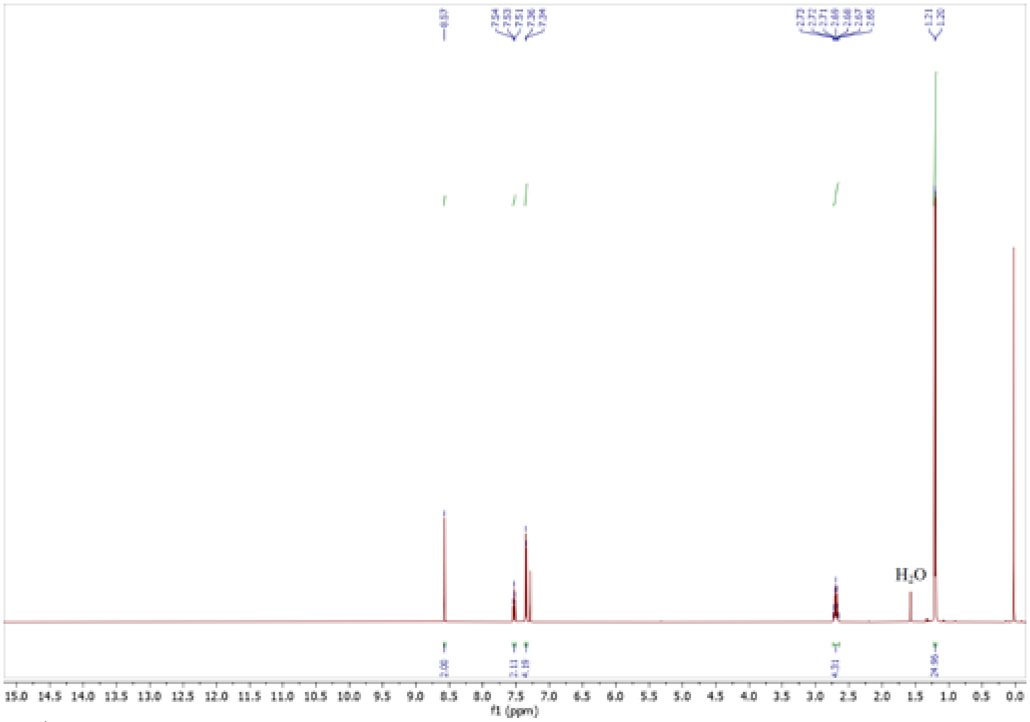

Figure S2. ^{1}H NMR (500 MHz, $CDCl_3$) spectrum of **PMDA-di^{i}PrAn** used in this work.

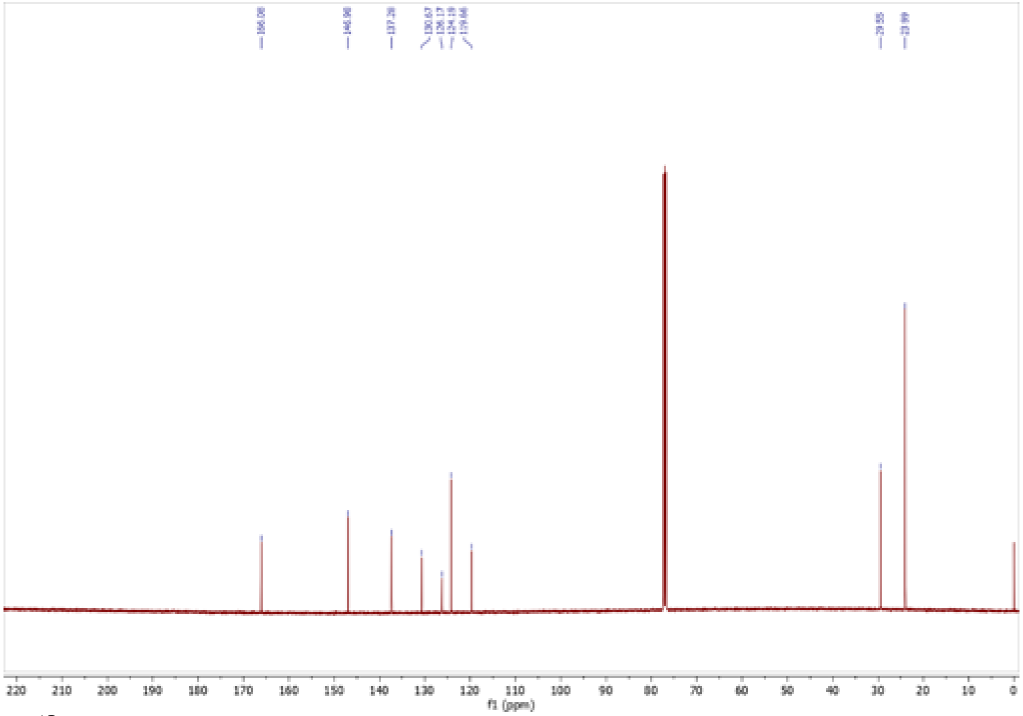

Figure S3. ^{13}C NMR (125 MHz, $CDCl_3$) spectrum of **PMDA-di^{i}PrAn** used in this work.

b. Synthesis of *N*,*N*'-bis(2,6-diisopropylphenyl)-1,4,5,8-naphthalenetetracarboxylic diimide (NTCDA-di^{i}PrAn).

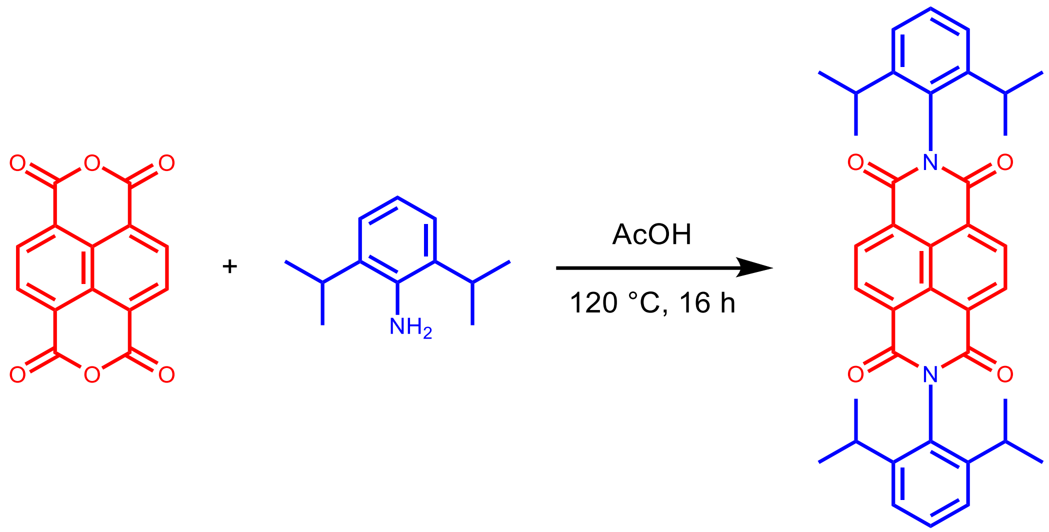

NTCDA **di^{i}PrAn** **NTCDA-di^{i}PrAn**

Figure S4. Synthesis of **NTCDA-di^{i}PrAn**.

This procedure was adapted from the literature.[2] **NTCDA** (1.34 g, 5.00 mmol, 1.00 equiv.) was suspended in AcOH (70 mL) in a 250 mL roundbottom flask equipped with a stir bar and reflux condenser. **Di^{i}PrAn** (6.32 mL, 33.5 mmol, 6.70 equiv.) was added to the suspension, and the mixture was heated to reflux (120 °C). The reaction mixture was allowed to stir at reflux for 16 h. The reaction was allowed to cool to room temperature and diluted with DI water (70 mL). The resulting precipitate was collected by filtration and rinsed with DI water (2 × 20 mL) and hexanes (3 × 20 mL). The solid was dried under vacuum at room temperature to yield *N*,*N*'-bis(2,6-diisopropylphenyl)-1,4,5,8-naphthalenetetracarboxylic diimide (**NTCDA-di^{i}PrAn**) as a pale orange-brown solid (2.05 g, 70% yield). ^{1}H NMR (500 MHz, $CDCl_3$): δ 8.91 (s, 4H), 7.55 (t, J = 7.8 Hz, 2H), 7.39 (d, J = 7.8 Hz, 4H), 2.73 (sept, J = 6.8 Hz, 4H), 1.19 (d, J = 6.9 Hz, 24H) ppm; ^{13}C NMR (125 MHz, $CDCl_3$): δ 163.0, 145.5, 131.6, 130.0, 127.7, 126.9, 124.3, 29.3, 24.0 ppm. These spectra are consistent with those reported in the literature.[3]

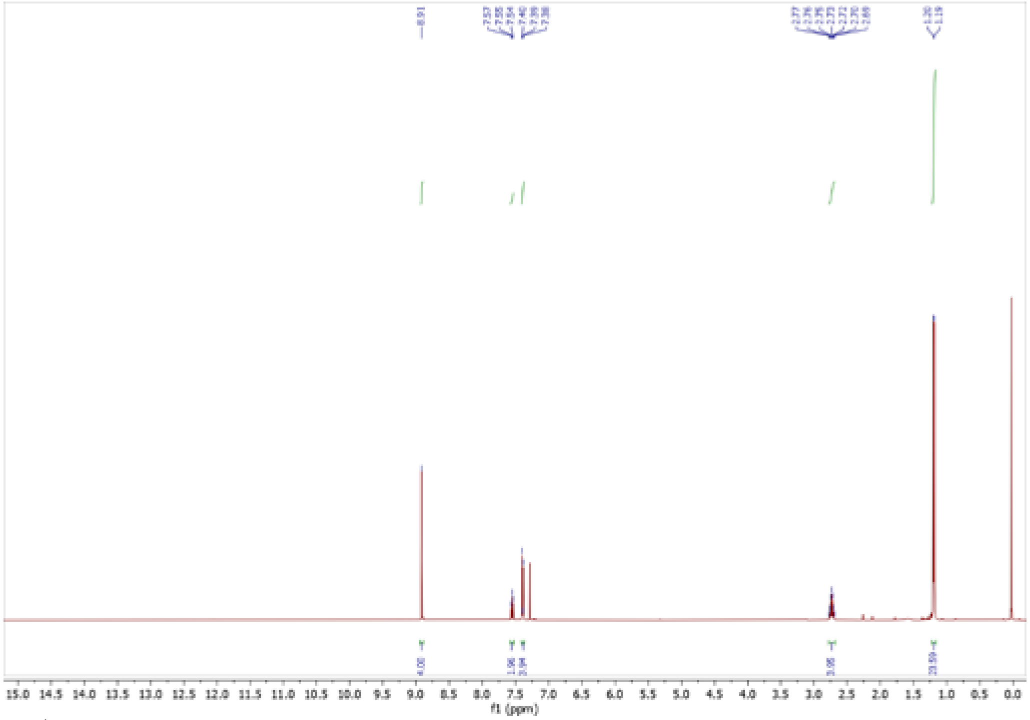

Figure S5. ^{1}H NMR (500 MHz, $CDCl_3$) spectrum of **NTCDA-di^{i}PrAn** used in this work.

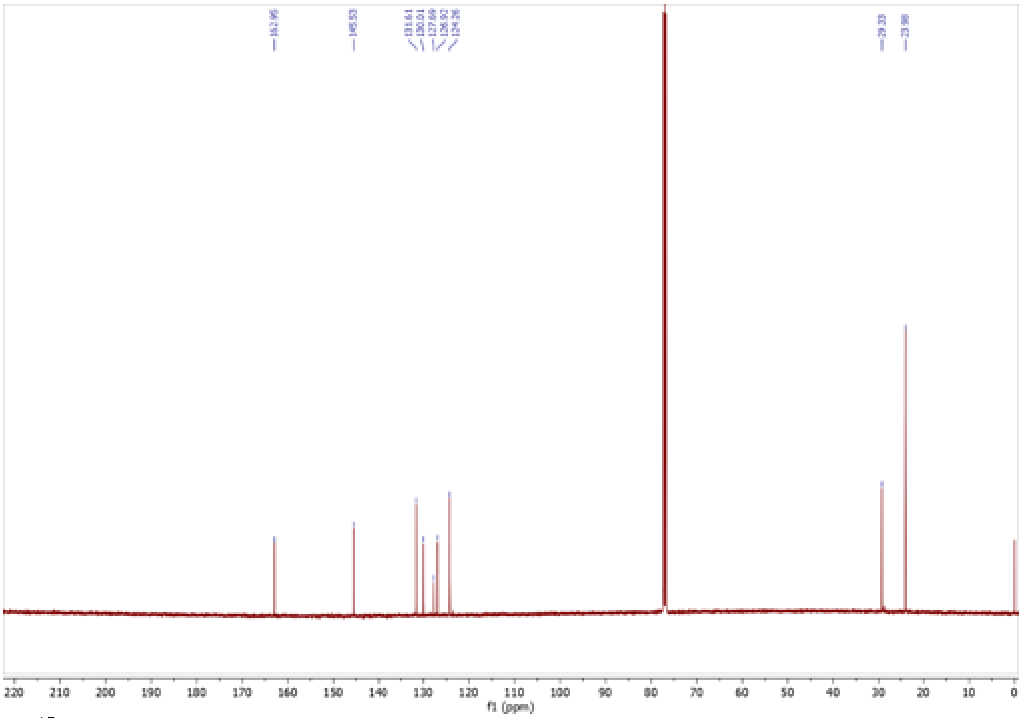

Figure S6. ^{13}C NMR (125 MHz, $CDCl_3$) spectrum of **NTCDA-di^{i}PrAn** used in this work.

c. **Synthesis of *N*,*N*'-bis(2,6-diisopropylphenyl)-3,4,9,10-perylenetetracarboxylic diimide (PTCDA-di^{i}PrAn).**

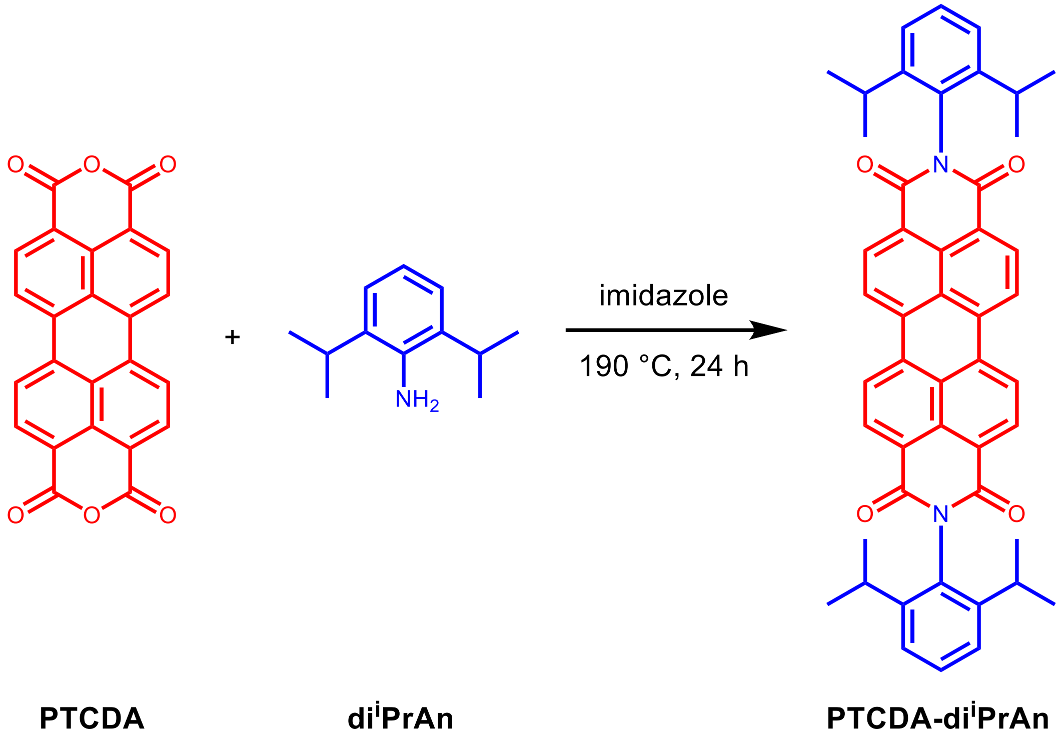

Figure S7. Synthesis of **PTCDA-di^{i}PrAn**.

This procedure was adapted from the literature.[4] **PTCDA** (785 mg, 2.00 mmol, 1.00 equiv.) and imidazole (6.0 g) were added to a 75 mL screw-cap reaction vessel equipped with a stir bar. **Di^{i}PrAn** (1.51 mL, 8.00 mmol, 4 equiv.) was added, and the flask was sealed. The reaction mixture was heated to 190 °C and allowed to stir at 190 °C for 24 h. At this time, the reaction mixture was allowed to cool to room temperature and then diluted with 95% EtOH (50 mL) and 2 M HCl (60 mL). The resulting mixture was stirred for 3 h and filtered. The resulting solid was rinsed with a 5:6 EtOH/2 M HCl mixture (110 mL), followed by a 1:1 EtOH/DI H_2O mixture (100 mL). The solid was extracted with acetone (400 mL). The acetone was collected, dried with anhydrous Na_2SO_4, and filtered. The solvent was then removed under reduced pressure to yield a purple-red solid, which was further purified by flash column chromatography (SiO_2, 50%→100% dichloromethane in hexanes) to yield *N*,*N*'-bis(2,6-diisopropylphenyl)-3,4,9,10-perylenetetracarboxylic diimide (**PTCDA-di^{i}PrAn**) as a bright red solid (403 mg, 28%). ^{1}H NMR (500 MHz, $CDCl_3$): δ 8.83 (d, J = 7.9 Hz, 4H), 8.77 (d, J = 8.0 Hz, 4H), 7.53 (t, J = 7.8 Hz, 2H), 7.39 (d, J = 7.8 Hz, 4H), 2.79 (sept, J = 6.8 Hz, 4H), 1.22 (d, J = 6.8 Hz, 24H) ppm; ^{13}C NMR (125 MHz, $CDCl_3$): δ 163.5, 145.7, 135.1, 132.1, 130.5, 130.2, 129.7, 126.9, 124.2, 123.4, 123.4, 29.2, 24.0 ppm. These spectra are consistent with those reported in the literature.[4]

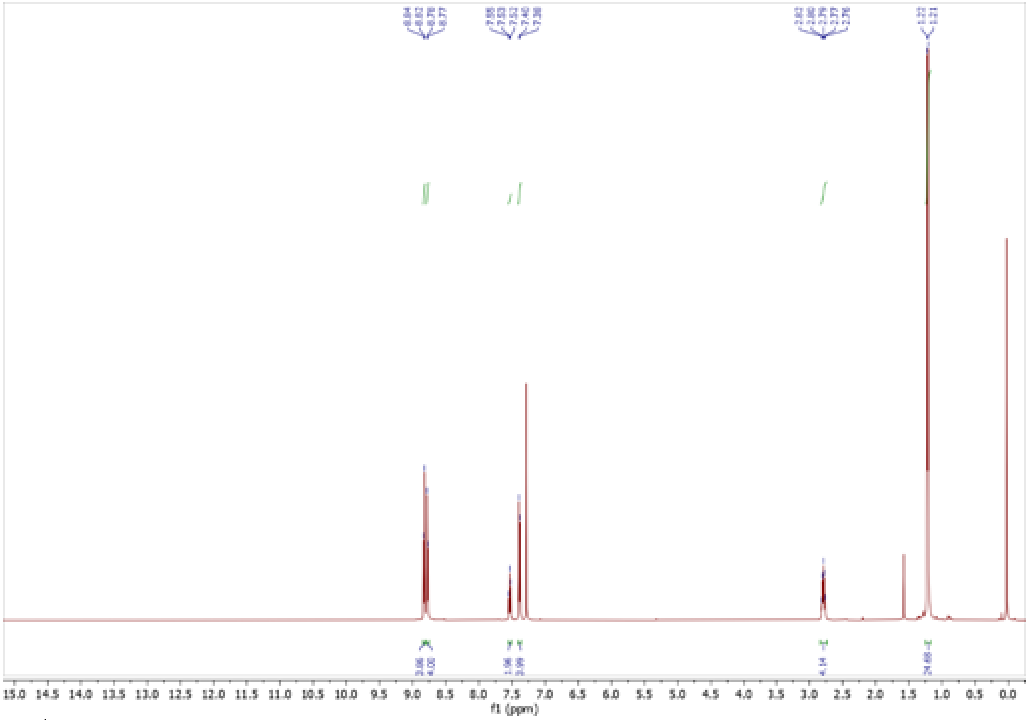

Figure S8. ^{1}H NMR (500 MHz, $CDCl_3$) spectrum of **PTCDA-di^{i}PrAn** used in this work.

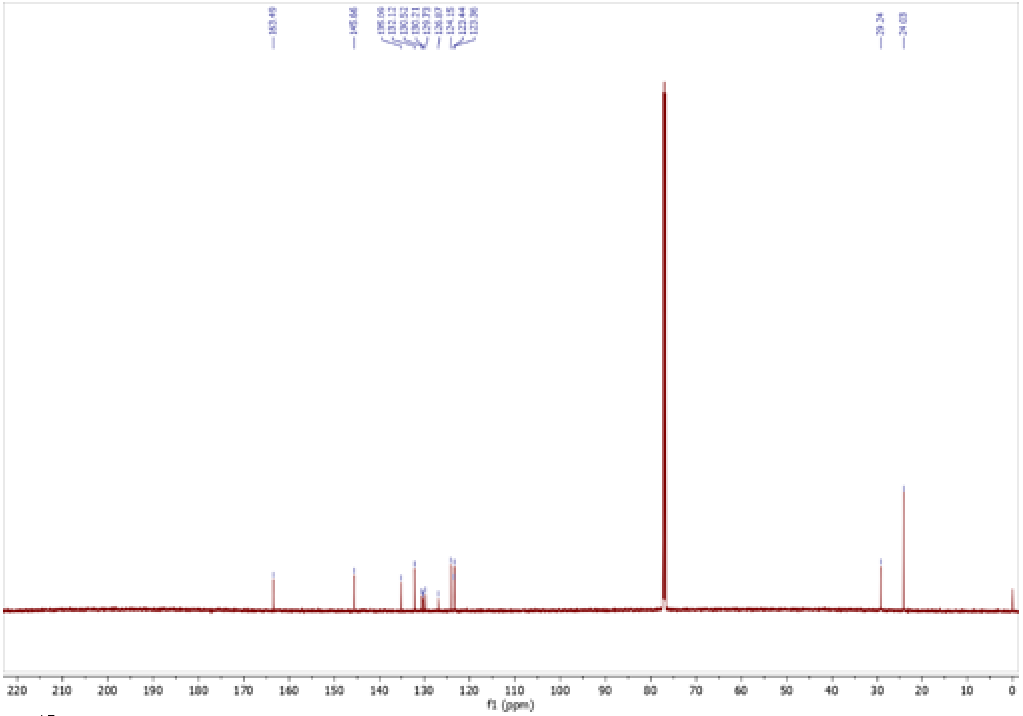

Figure S9. ^{13}C NMR (125 MHz, $CDCl_3$) spectrum of **PTCDA-di^{i}PrAn** used in this work.

3. Polymer synthesis and characterization.

General Procedure A. The following procedure was adapted from literature procedure for synthesizing molecular diimides.[5] A 100 mL round-bottom flask or a 75 mL screw-cap reaction vessel (if the amine boils below 130 °C) was equipped with a stir bar. Imidazole (17.7 g) and the dianhydride were added to the round-bottom flask. If the amine was solid, it was added with the imidazole and the imide to the reaction vessel. If the amine was a liquid, it was added after the solids were added to the reaction vessel. The reaction flask was sealed, and the reaction mixture was heated to 130 °C and allowed to stir at 130 °C for 24 h. The reaction mixture was allowed to cool to room temperature. The resulting solid was then diluted with DI water (300 mL) and centrifuged. The aqueous layer was decanted, and the resulting solid residue was rinsed with acetone (300 mL) and filtered, rinsing with additional acetone. The solid was further dried in air before being Soxhlet washed with chloroform for 48 h at 90 °C. The solid was then transferred to a scintillation vial and dried under vacuum at 120 °C for 16 h prior to characterization.

a. Synthesis and characterization of PMDA-pPDA from pyromellitic dianhydride (PMDA) and *para*-phenylene diamine (pPDA).

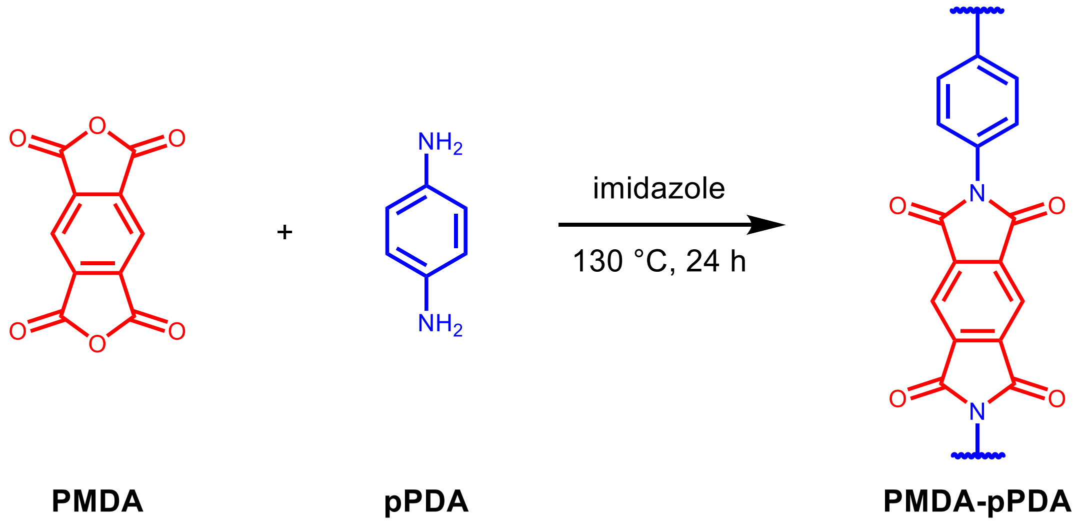

Figure S10. Scheme for the synthesis of **PMDA-pPDA** from **PMDA** and **pPDA**.

Following **General Procedure A**, imidazole (17.7 g), **PMDA** (436 mg, 2.00 mmol, 1.00 equiv.) and **pPDA** (216 mg, 2.00 mmol, 1.00 equiv.) were added to a 100 mL round-bottom flask and stirred at 130 °C for 24 h. The resulting solid was washed and dried according to the procedure to yield **PMDA-pPDA** (259 mg, 45% yield) as a light brown solid.

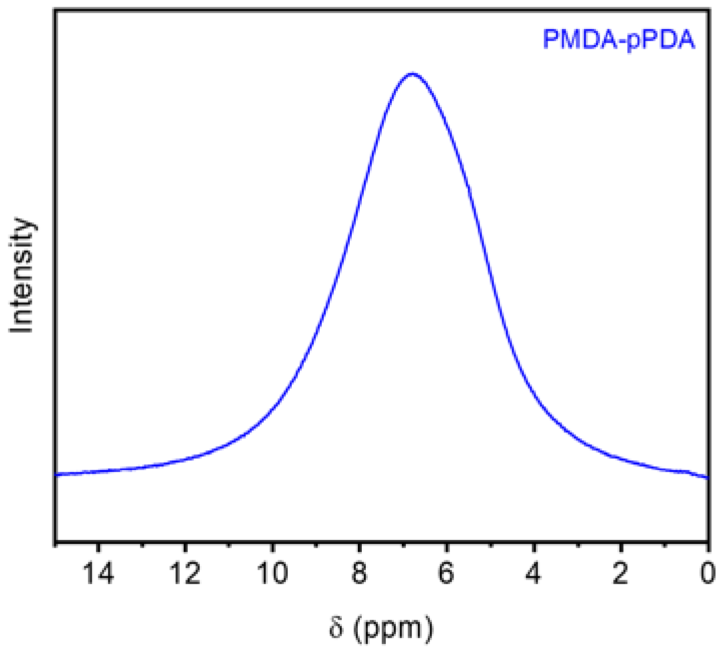

Figure S11. ^1H MAS SSNMR (500 MHz) spectrum of **PMDA-pPDA**, collected at a spinning speed of 20 kHz. The peak centered near 7 ppm indicates that this material contains only aromatic protons.

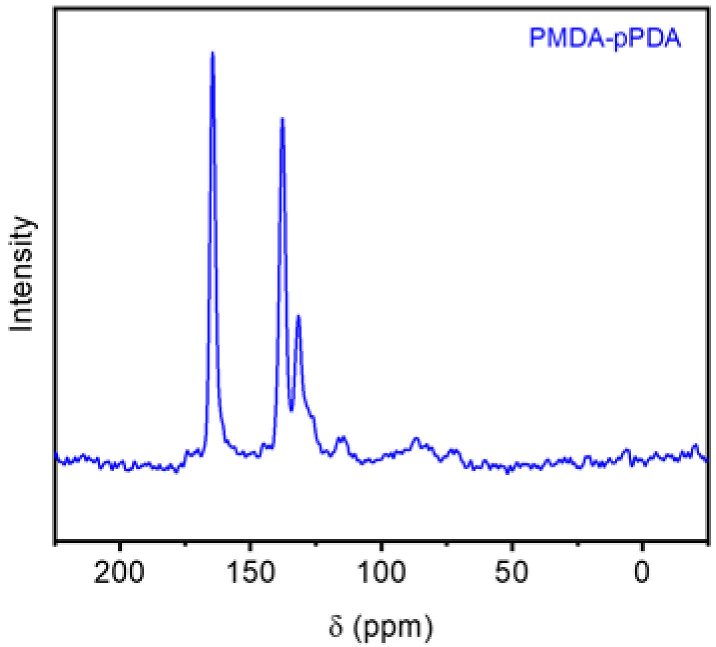

Figure S12. CP ^{13}C MAS SSNMR (125 MHz) spectrum of **PMDA-pPDA**, with a ^1H–^{13}C contact time of 10 ms, collected at a spinning speed of 20 kHz.

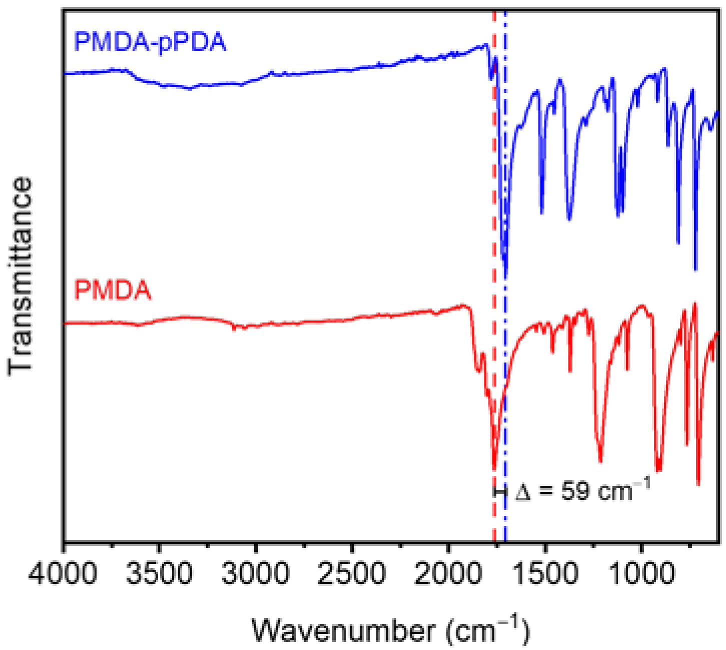

Figure S13. ATR-IR spectra for **PMDA** and **PMDA-pPDA**. The shift in the carbonyl stretching frequency between the dianhydride and the diimide is indicated.

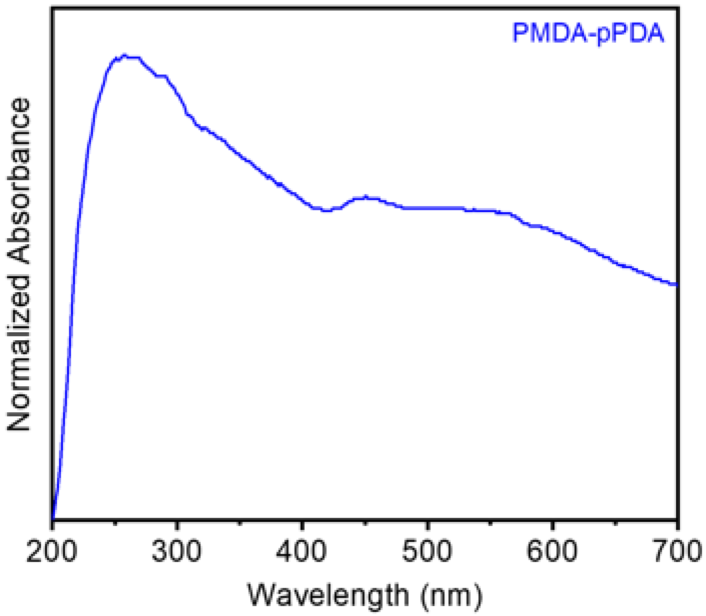

Figure S14. Solid-state UV-Vis absorption spectrum of **PMDA-pPDA**.

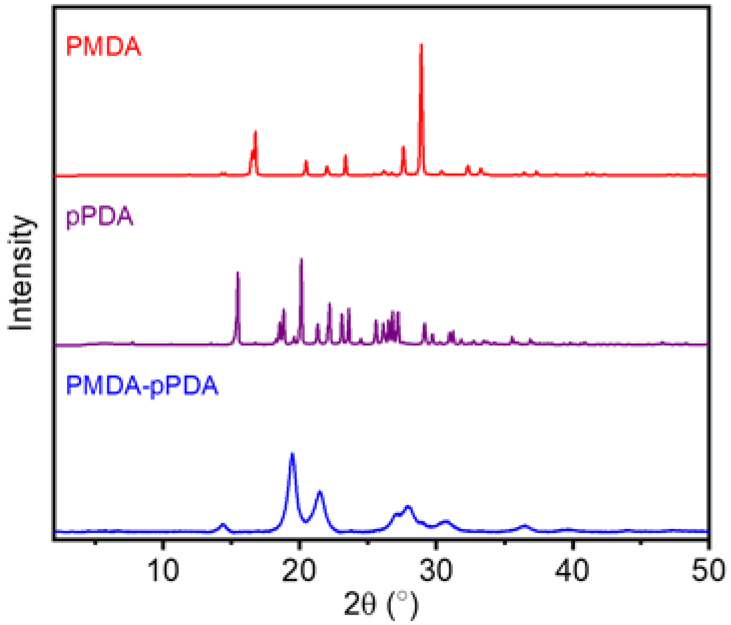

Figure S15. PXRD patterns (λ = 1.54 Å) of **PMDA**, **pPDA** and **PMDA-pPDA.** These patterns indicate that **PMDA-pPDA** is a microcrystalline polymer and is not contaminated with residual **pPDA** or **PMDA**.

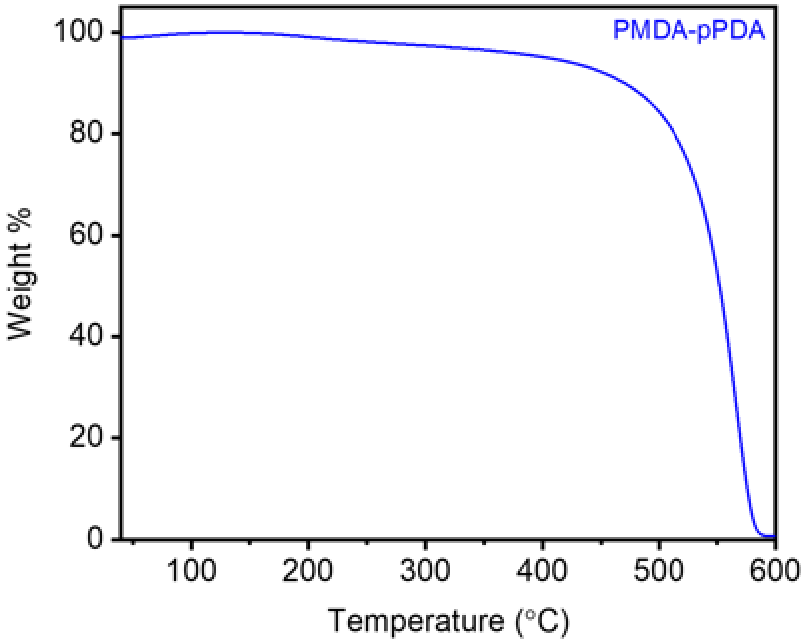

Figure S16. Thermogravimetric decomposition profile in air for **PMDA-pPDA**, indicating that it is stable below approximately 450 °C.

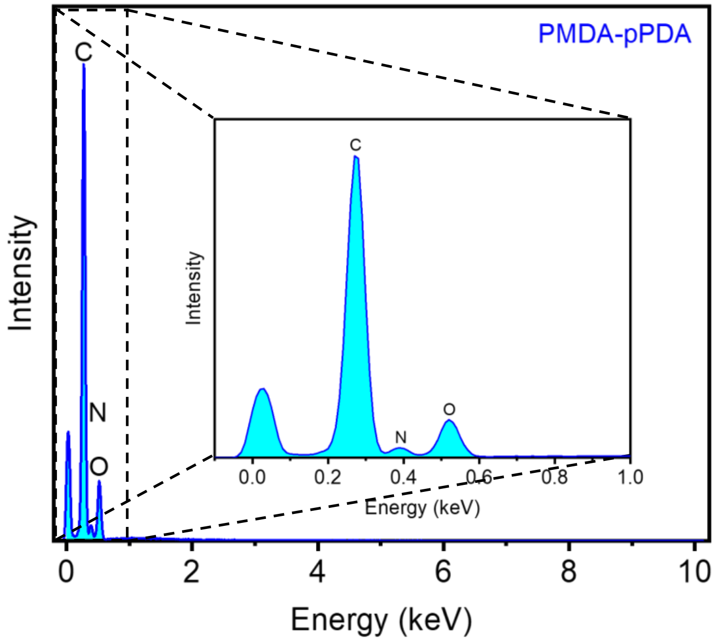

Figure S17. EDS spectrum of **PMDA-pPDA**.

Table S1. Tabulated EDS data for **PMDA-pPDA**.

Element	Line Type	Wt %	Atomic %	Theoretical Atomic %[1]
C	K series	68.78	73.57	72.73
O	K series	19.22	15.43	9.09
N	K series	11.99	11.00	18.18
Total:		100.00	100.00	100.00

[1]Excludes hydrogen.

Table S2. Tabulated combustion analysis data for **PMDA-pPDA**.

Element	Wt %	Theoretical Wt %
C	62.52	66.21
O	23.34[1]	22.05
H	2.67	2.08
N	11.47	9.65
Total:	100.00	100.00

[1]The remaining mass not attributed to C, H, and N was assumed to come from O, as O was not directly analyzed during combustion analysis.

b. Synthesis and characterization of NTCDA-pPDA from 1,4,5,8-naphthalene tetracarboxylic dianhydride (NTCDA) and *para*-phenylene diamine (pPDA).

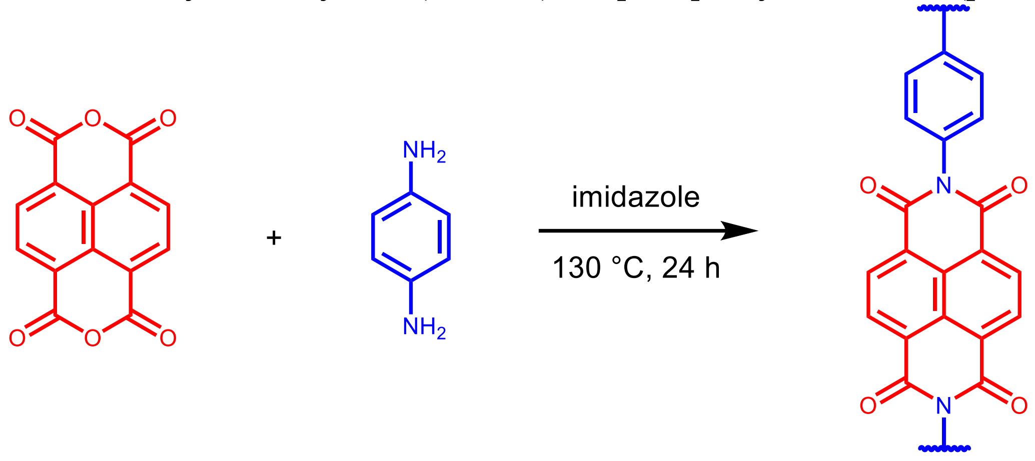

NTCDA **pPDA** **NTCDA-pPDA**

Figure S18. Scheme for the synthesis of **NTCDA-pPDA** from **NTCDA** and **pPDA**.

Following **General Procedure A**, imidazole (17.7 g), **NTCDA** (268 mg, 1.00 mmol, 1.00 equiv.) and **pPDA** (108 mg, 1.00 mmol, 1.00 equiv.) were added to a 100 mL round-bottom flask stirred at 130 °C for 24 h. The resulting solid was washed and dried according to the procedure to yield **NTCDA-pPDA** (235 mg, 69% yield) as a dark brown solid.

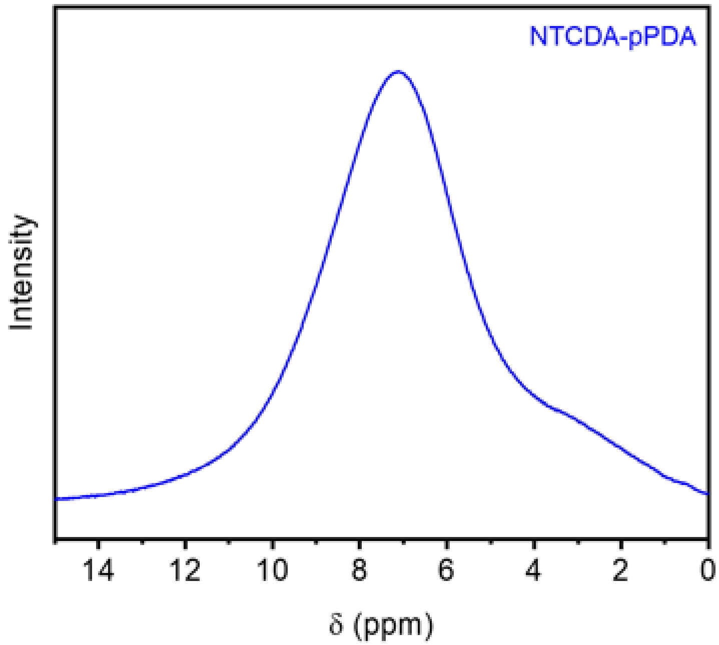

Figure S19. ^{1}H MAS SSNMR (500 MHz) spectrum of **NTCDA-pPDA**, collected at a spinning speed of 20 kHz. The peak centered near 7 ppm indicates that this material contains only aromatic protons.

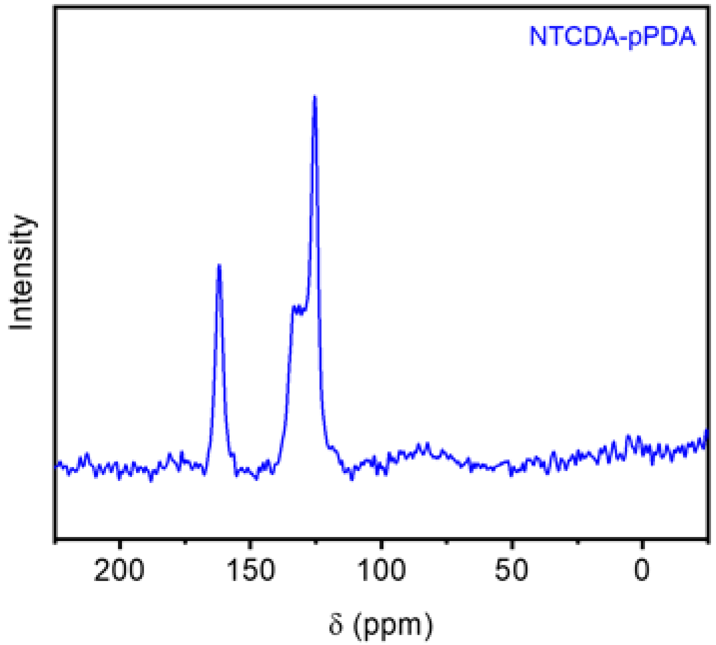

Figure S20. CP ^{13}C MAS SSNMR (125 MHz) spectrum of **NTCDA-pPDA** with a ^{1}H–^{13}C contact time of 2 ms, collected at a spinning speed of 20 kHz.

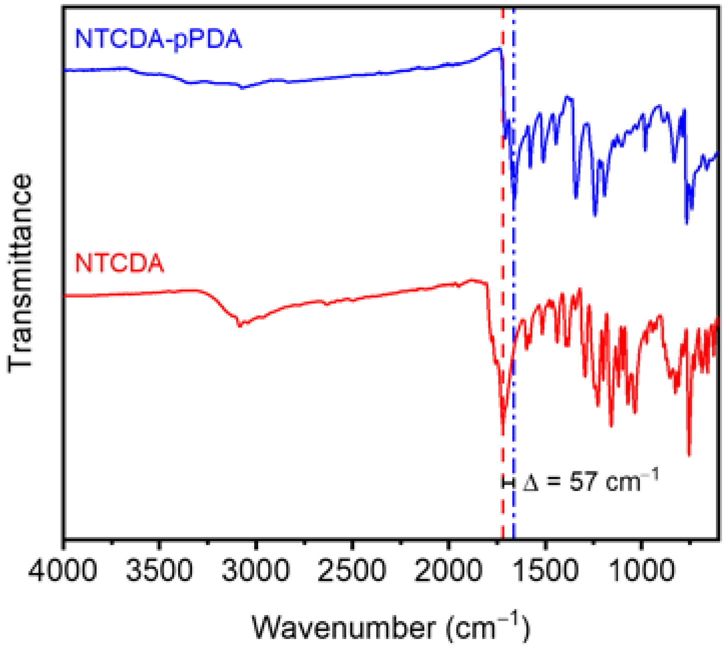

Figure S21. ATR-IR spectra for **NTCDA** and **NTCDA-pPDA**. The shift in the carbonyl stretching frequency between the dianhydride and the diimide is indicated.

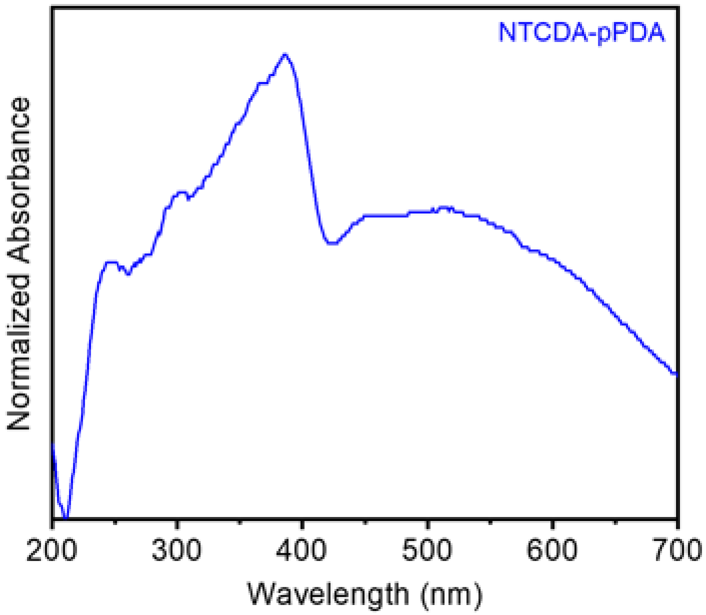

Figure S22. Solid-state UV-Vis absorption spectrum of **NTCDA-pPDA**.

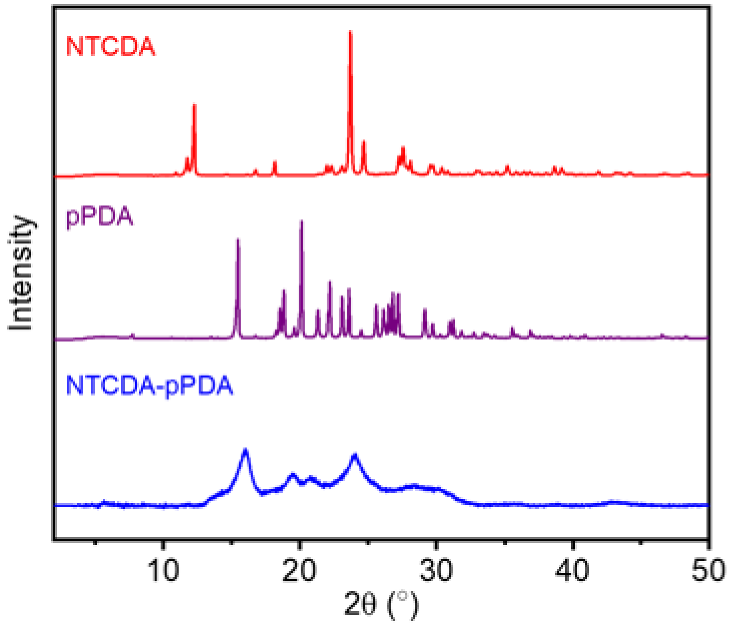

Figure S23. PXRD patterns (λ = 1.54 Å) of **NTCDA**, **pPDA** and **NTCDA-pPDA**. These patterns indicate that **NTCDA-pPDA** is a microcrystalline polymer and is not contaminated with **NTCDA** or **pPDA**.

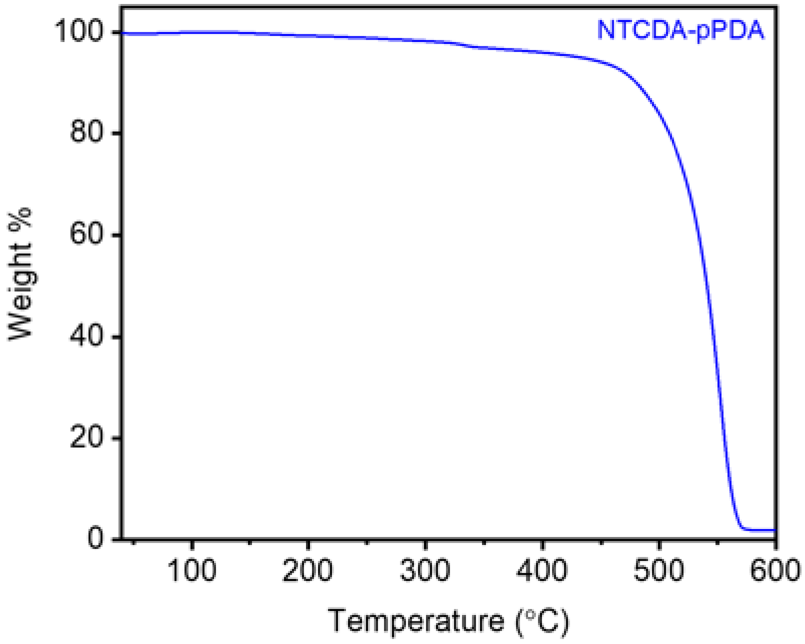

Figure S24. Thermogravimetric decomposition profile in air for **NTCDA-pPDA**, indicating that it is stable below 450 °C.

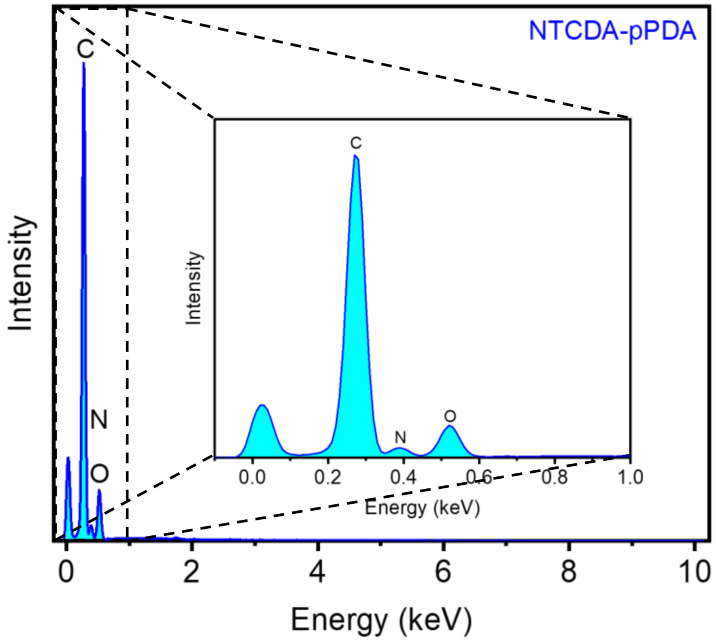

Figure S25. EDS spectrum of **NTCDA-pPDA**.

Table S3. Tabulated EDS data for **NTCDA-pPDA**.

Element	Line Type	Wt %	Atomic %	Theoretical Atomic %[1]
C	K series	69.75	74.30	76.93
O	K series	17.08	13.66	15.38
N	K series	13.17	12.03	7.69
Total:		100.00	100.00	100.00

[1]Excludes hydrogen.

Table S4. Tabulated combustion analysis data for **NTCDA-pPDA**.

Element	Wt %	Theoretical Wt %
C	65.04	70.59
O	21.27[1]	18.81
H	10.81	8.23
N	2.88	2.37
Total:	100.00	100.00

[1]The remaining mass not attributed to C, H, and N was assumed to come from O, as O was not directly analyzed during combustion analysis.

c. **Synthesis and characterization of PTCDA-pPDA from 3,4,9,10-perylene tetracarboxylic dianhydride (PTCDA) and *para*-phenylene diamine (pPDA)**

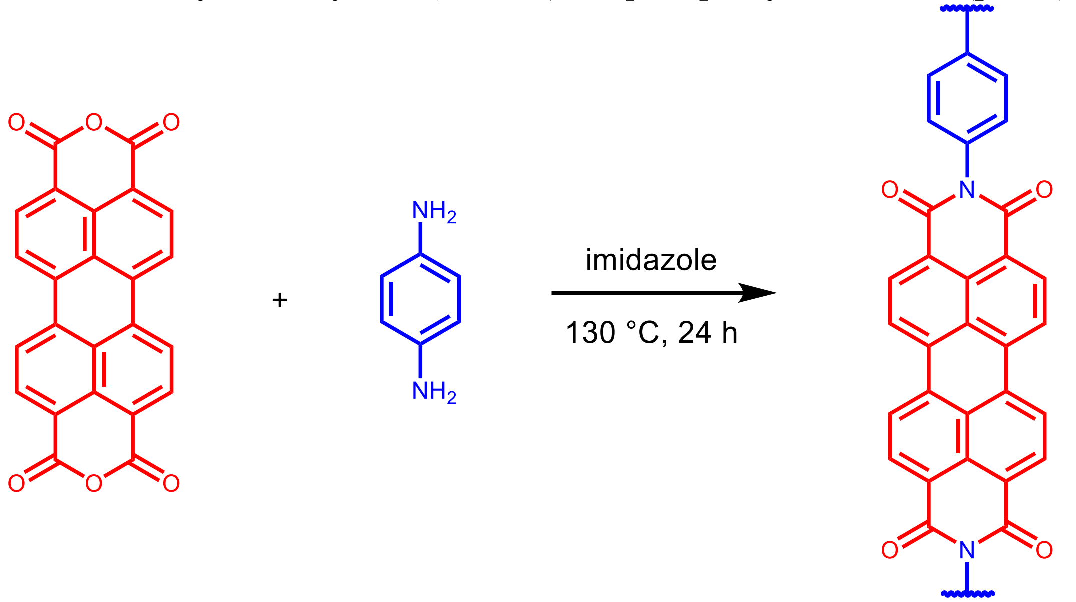

PTCDA **pPDA** **PTCDA-pPDA**

Figure S26. Scheme for the synthesis of **PTCDA-pPDA** from **PTCDA** and **pPDA**.

Following **General Procedure A**, imidazole (17.7 g), **PTCDA** (392 mg, 1.00 mmol, 1.00 equiv) and **pPDA** (108 mg, 1.00 mmol, 1.00 equiv.) were added to a 100 mL round-bottom flask and stirred at 130 °C for 24 h. The resulting solid was washed and dried according to the procedure to yield **PTCDA-pPDA** (321 mg, 69% yield) a deep burgundy solid.

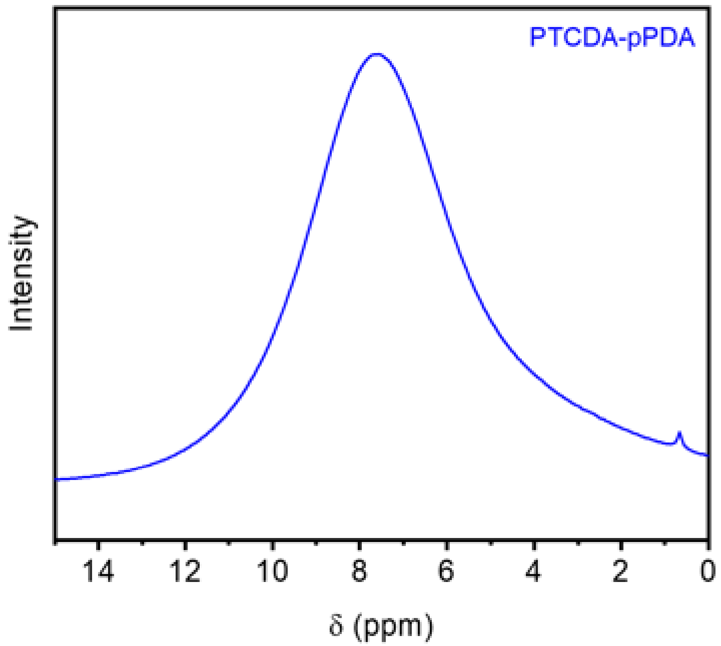

Figure S27. ^{1}H MAS SSNMR (500 MHz) spectrum of **PTCDA-pPDA**, collected at a spinning speed of 20 kHz. The peak centered near 7.5 ppm indicates that this material contains only aromatic protons.

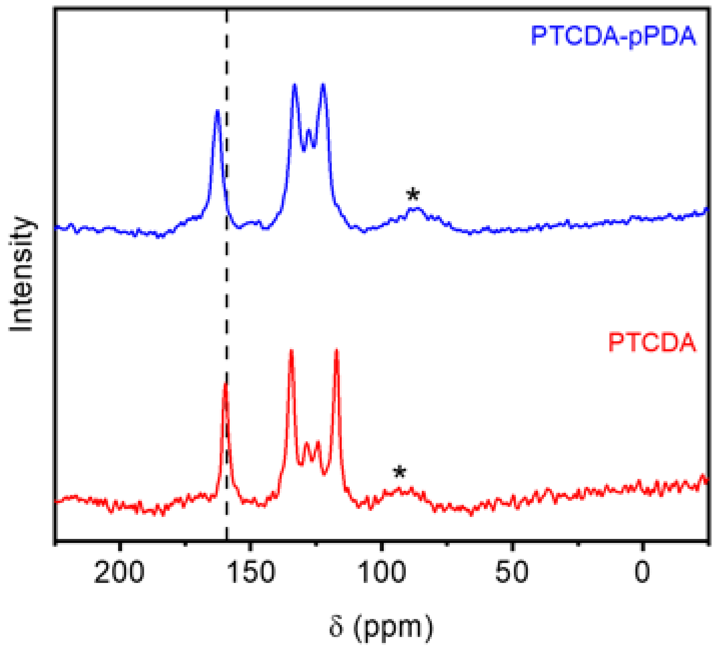

Figure S28. CP ^{13}C MAS SSNMR (125 MHz) spectra of **PTCDA** and **PTCDA-pPDA**, with a ^{1}H–^{13}C contact time of 1 ms and 5 ms, respectively, collected at a spinning speed of 20 kHz. Persistent background signals are indicated (*).

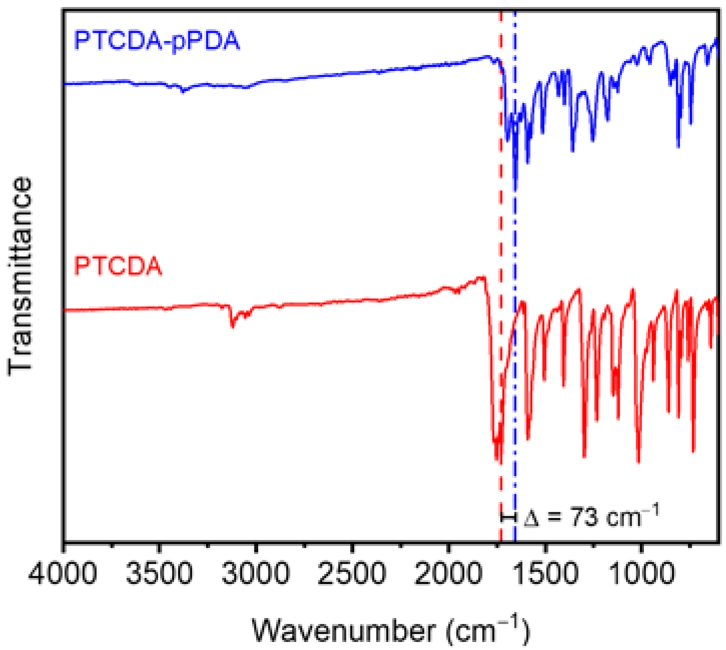

Figure S29. ATR-IR spectra for **PTCDA** and **PTCDA-pPDA**. The shift in the carbonyl stretching frequency between the dianhydride and the diimide is indicated.

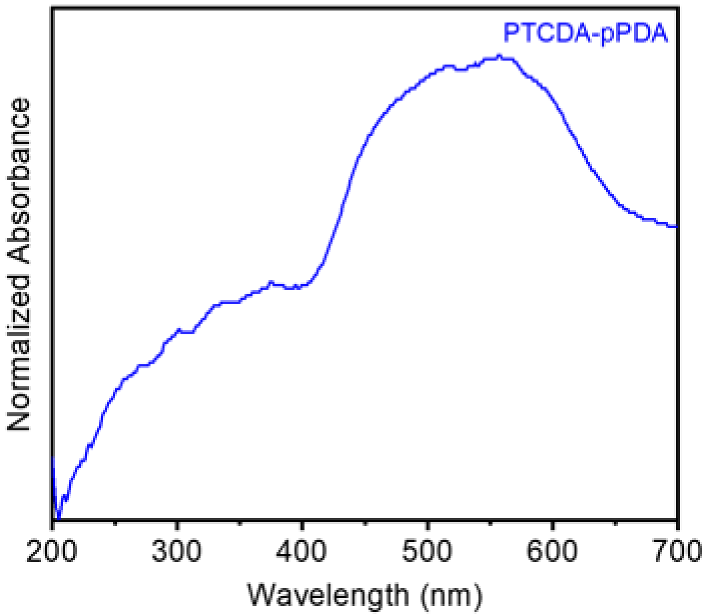

Figure S30. Solid-state UV-Vis absorption spectrum of **PTCDA-pPDA**.

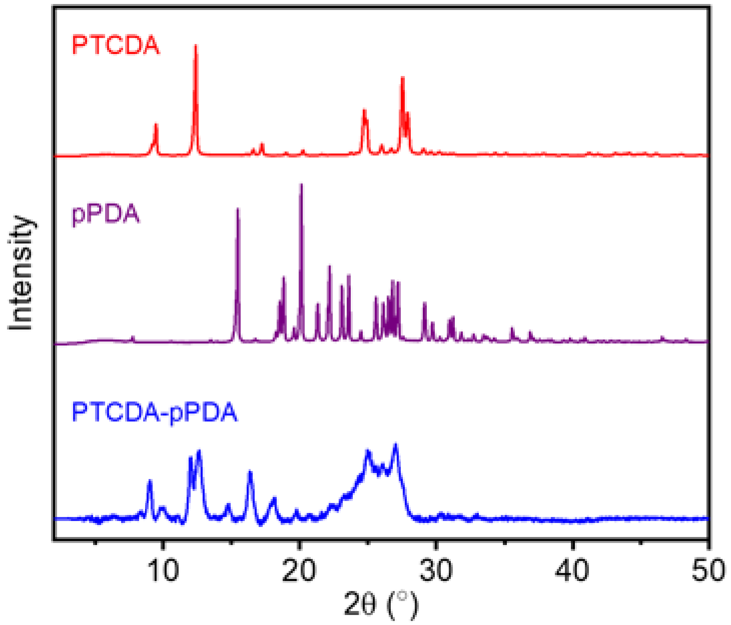

Figure S31. PXRD patterns (λ = 1.54 Å) of **PTCDA**, **pPDA**, and **PTCDA-pPDA**. These patterns indicate that **PTCDA-pPDA** is a microcrystalline polymer and is likely not contaminated with **PTCDA** or **pPDA**.

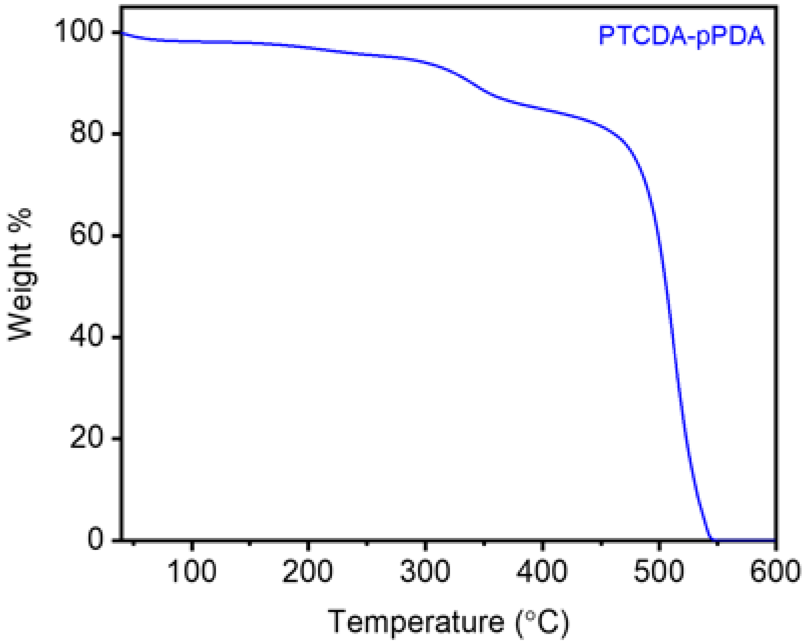

Figure S32. Thermogravimetric decomposition profile in air for **PTCDA-pPDA**, indicating that it is stable below 450 °C. The gradual weight loss from this material upon heating is likely due to the loss of solvent/water, given its microporosity (**Figure S78** and **Figure S79**).

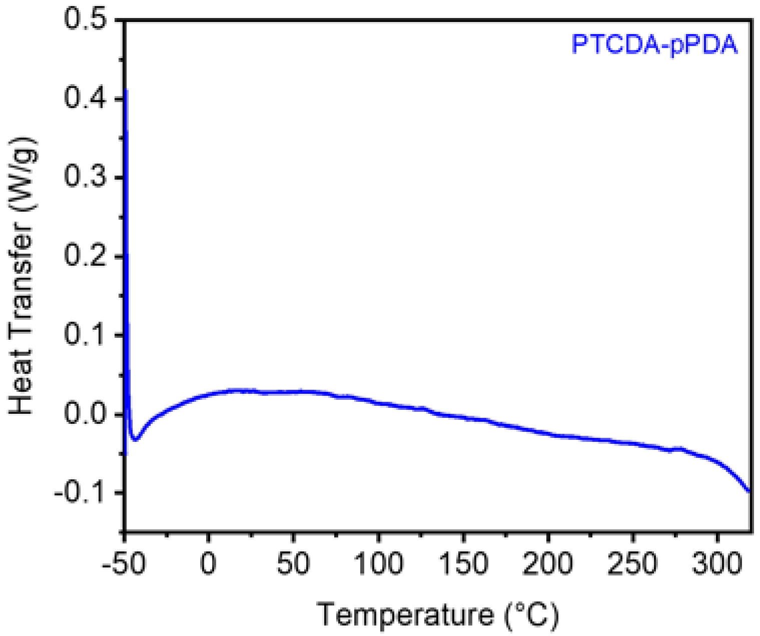

Figure S33. DSC profile under N_2 for **PTCDA-pPDA**, showing the second heating curve from −50 °C to 320 °C. No significant phase changes were observed for this material in this temperature range.

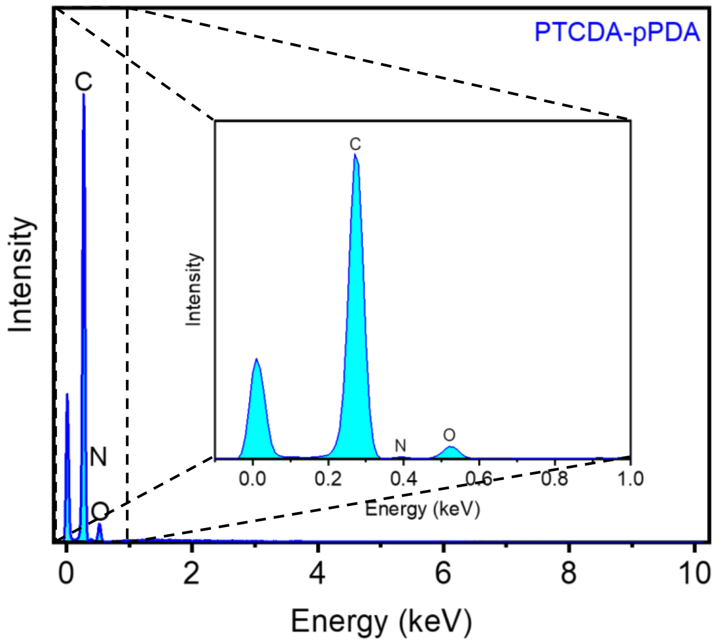

Figure S34. EDS spectrum of **PTCDA-pPDA**.

Table S5. Tabulated EDS data for **PTCDA-pPDA**.

Element	Line Type	Wt %	Atomic %	Theoretical Atomic %[1]
C	K series	86.04	88.69	83.33
O	K series	9.33	7.24	11.11
N	K series	4.63	4.07	5.56
Total:		100.00	100.00	100.00

[1]Excludes hydrogen.

Table S6. Tabulated combustion analysis data for **PTCDA-pPDA**.

Element	Wt %	Theoretical Wt %
C	71.71	77.58
O	17.38[1]	13.78
H	2.93	2.60
N	7.98	6.03
Total:	100.00	100.00

[1]The remaining mass not attributed to C, H, and N was assumed to come from O, as O was not directly analyzed during combustion analysis.

d. **Synthesis and characterization of PTCDA-chex from 3,4,9,10-perylene tetracarboxylic dianhydride (PTCDA) and 1,4-diaminocyclohexane (chex).**

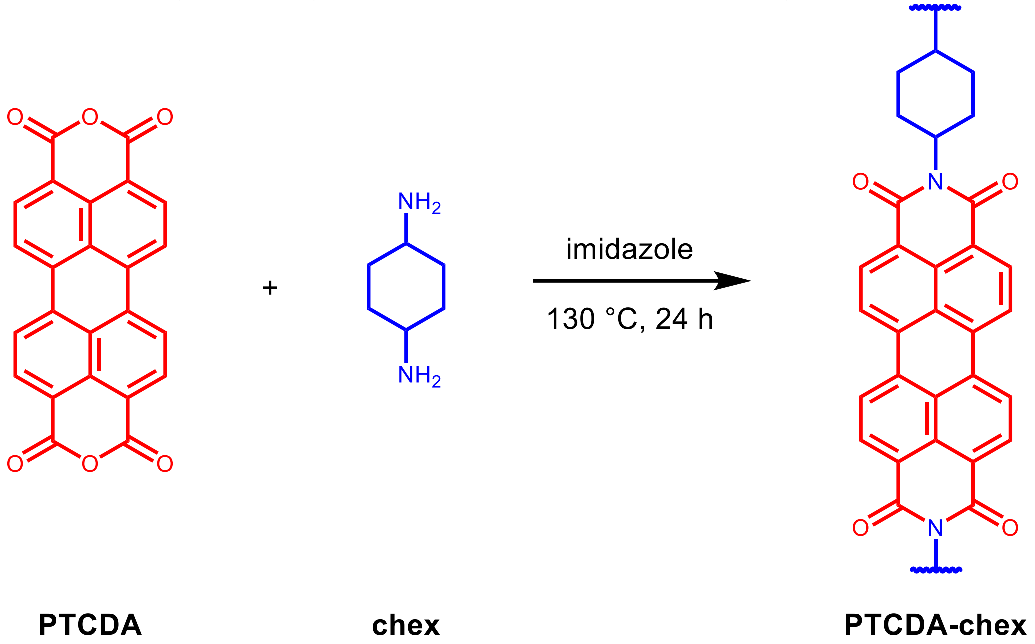

Figure S35. Scheme for the synthesis of **PTCDA-chex** from **PTCDA** and **chex**.

Following **General Procedure A**, imidazole (17.7 g), PTCDA (392 mg, 1.00 mmol, 1.00 equiv.) and chex (114 mg, 1.00 mmol, 1.00 equiv.) were added to a 100 mL round-bottom flask and stirred at 130 °C for 24 h. The resulting solid was washed and dried according to the procedure to yield **PTCDA-chex** (358 mg, 76% yield) as a purple-black solid.

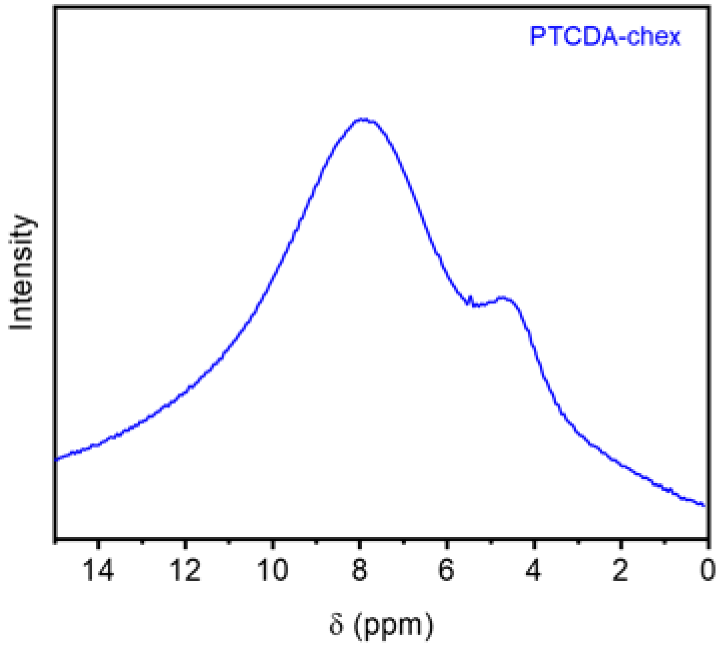

Figure S36. ^1H MAS SSNMR (500 MHz) spectrum of **PTCDA-chex**, collected at a spinning speed of 20 kHz. The peak centered near 8 ppm corresponds to the aromatic protons, and the peak near 4 ppm corresponds to the protons adjacent to the amines in the proposed structure.

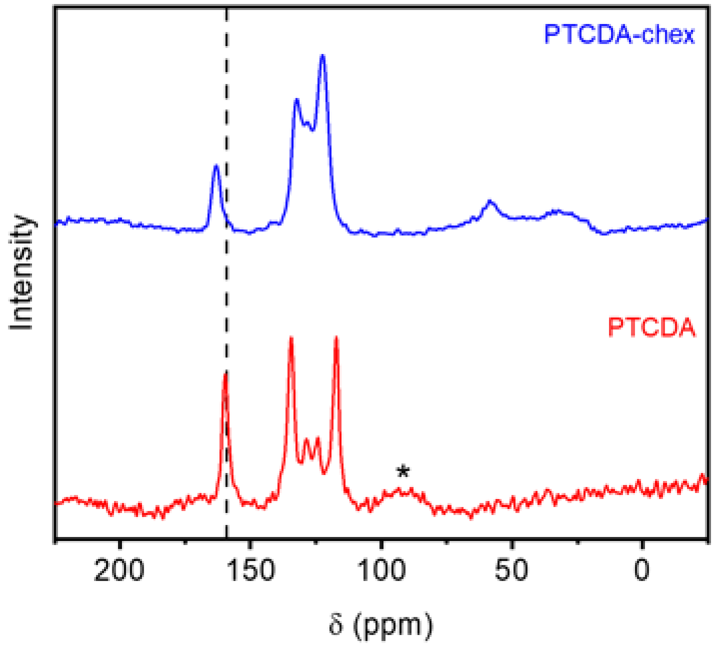

Figure S37. CP ^{13}C MAS-SSNMR (125 MHz) spectra of **PTCDA** and **PTCDA-chex**, with a ^1H–^{13}C contact time of 1 ms and 0.5 ms, respectively, collected at a spinning speed of 20 kHz. Persistent background signals are indicated (*).

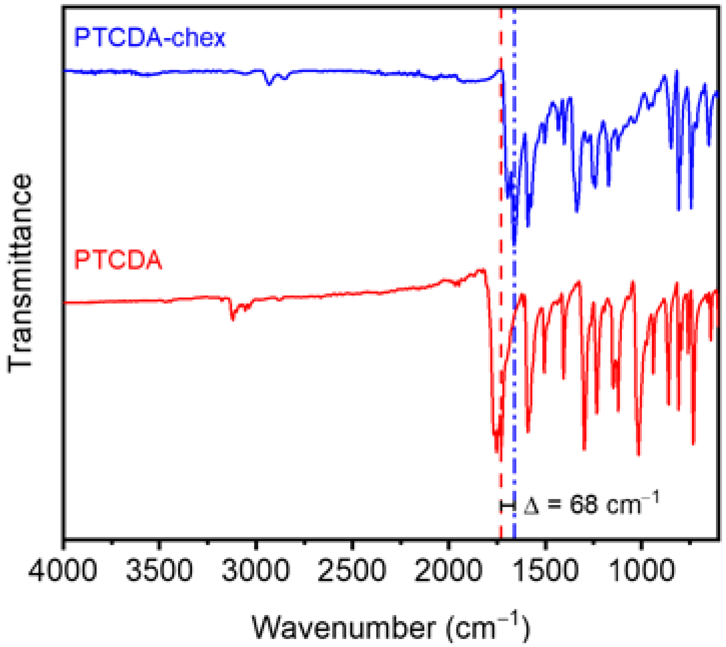

Figure S38. ATR-IR spectra for **PTCDA** and **PTCDA-chex**. The shift in the carbonyl stretching frequency between the dianhydride and the diimide is indicated.

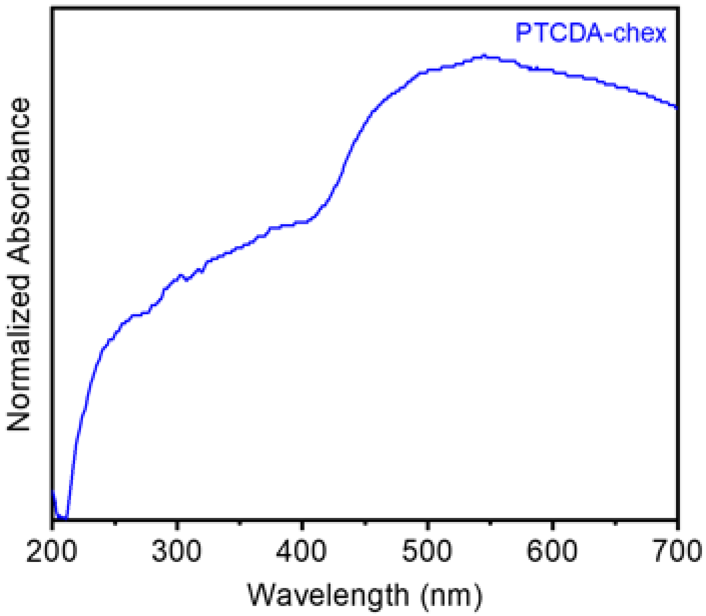

Figure S39. Solid-state UV-Vis absorption spectrum of **PTCDA-chex**.

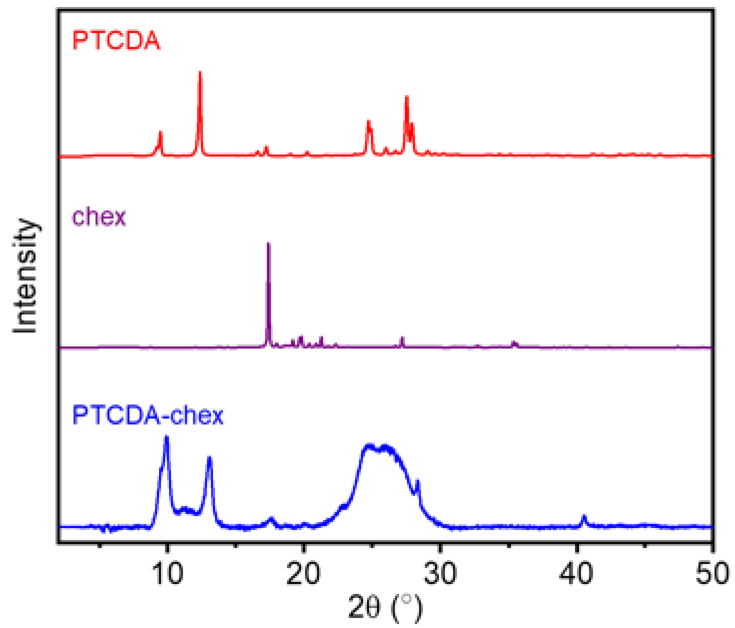

Figure S40. PXRD patterns (λ = 1.54 Å) of **PTCDA**, **chex** and **PTCDA-chex**. These patterns indicate that **PTCDA-chex** is a microcrystalline polymer and is likely not contaminated with **PTCDA** or **chex**.

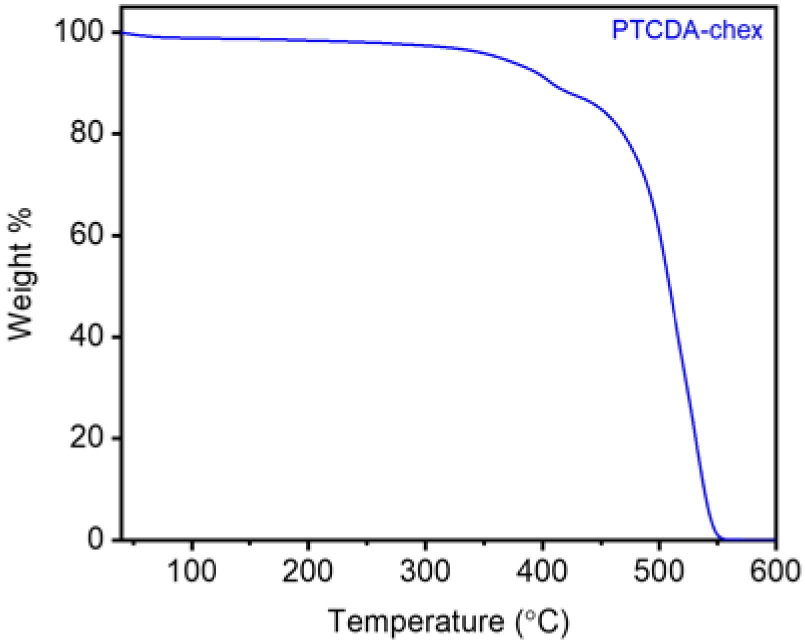

Figure S41. Thermogravimetric decomposition profile in air for **PTCDA-chex**, indicating that it is stable below 400 °C.

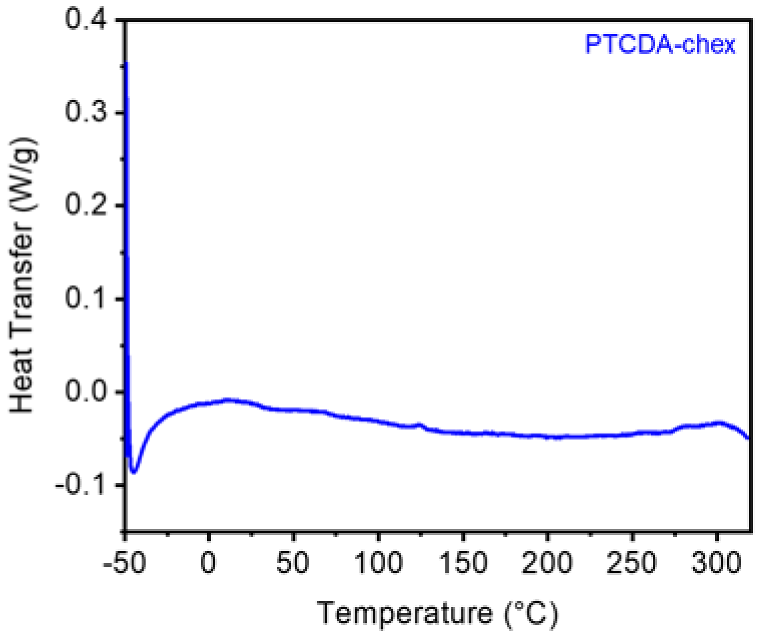

Figure S42. DSC profile under N_2 for **PTCDA-chex**, showing the second heating curve from −50 °C to 320 °C. No significant phase changes were observed for this material in this temperature range.

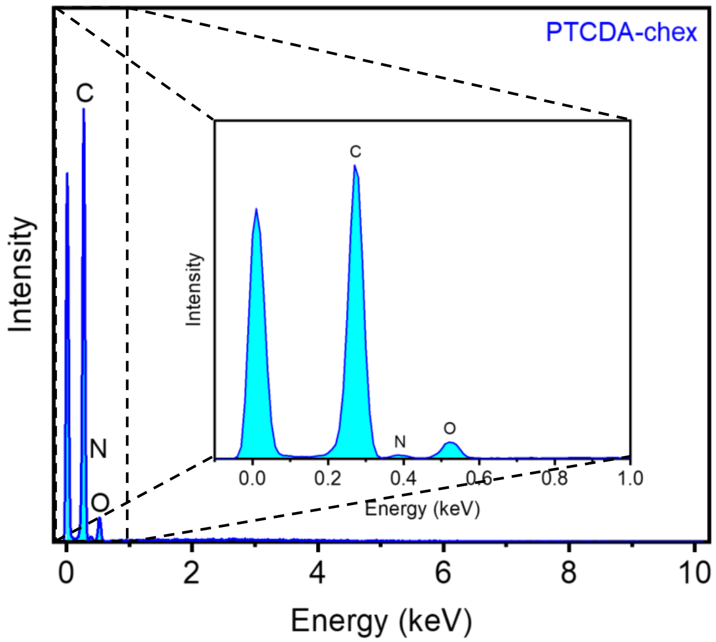

Figure S43. EDS spectrum of **PTCDA-chex**.

Table S7. Tabulated EDS data for **PTCDA-chex**.

Element	Line Type	Wt %	Atomic %	Theoretical Atomic %[1]
C	K series	76.93	84.14	83.33
O	K series	17.15	8.43	11.11
N	K series	13.22	7.42	5.56
Total:		100.00	100.00	100.00

[1]Excludes hydrogen.

Table S8. Tabulated combustion analysis data for **PTCDA-chex**.

Element	Wt %	Theoretical Wt %
C	73.14	76.59
O	15.37[1]	13.60
H	3.81	3.86
N	7.68	5.95
Total:	100.00	100.00

[1]The remaining mass not attributed to C, H, and N was assumed to come from O, as O was not directly analyzed during combustion analysis.

e. Synthesis and characterization of PTCDA-en from 3,4,9,10-perylene tetracarboxylic dianhydride (PTCDA) and 1,2-ethylenediamine (en).

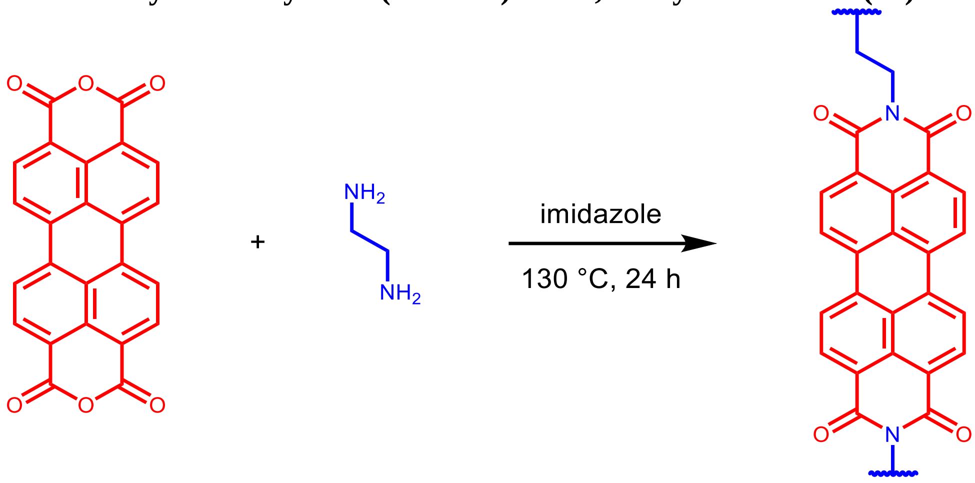

PTCDA **en** **PTCDA-en**

Figure S44. Scheme for the synthesis of **PTCDA-en** from **PTCDA** and **en**.

Following **General Procedure A**, imidazole (17.7 g), **PTCDA** (392 mg, 1.00 mmol, 1.00 equiv.) and **en** (100 µl, 1.00 mmol, 1.00 equiv.) were added to a 75 mL screw-cap reaction vessel and stirred at 130 °C for 24 h. The resulting solid was washed and dried according to the procedure to yield **PTCDA-en** (418 mg, quantitative yield) as a purple-black solid.

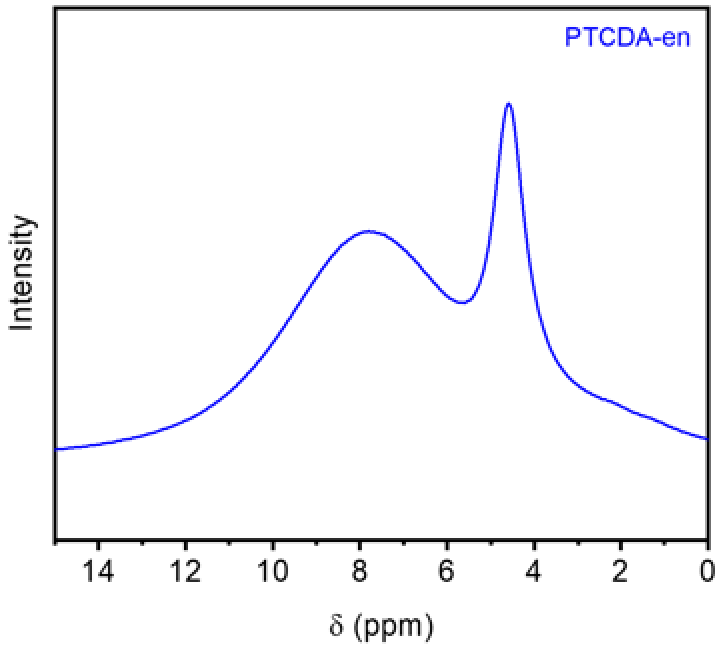

Figure S45. ^{1}H MAS SSNMR (500 MHz) spectrum of **PTCDA-en**, collected at a spinning speed of 20 kHz. The peak centered near 8 ppm corresponds to the aromatic protons, and the peak near 4 ppm corresponds to the protons adjacent to the amines in the proposed structure.

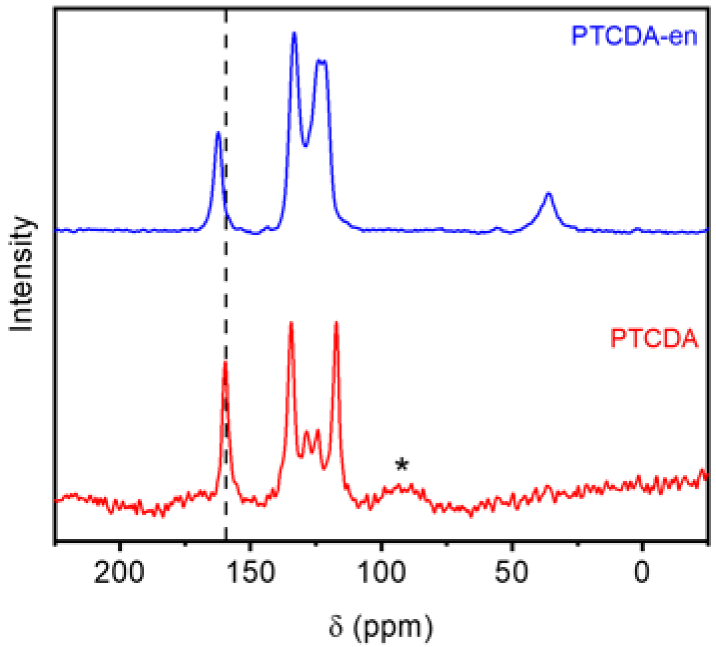

Figure S46. CP ^{13}C MAS SSNMR (125 MHz) spectra of **PTCDA** and **PTCDA-en**, both with a ^{1}H–^{13}C contact time of 1 ms, collected at a spinning speed of 20 kHz. Persistent background signals are indicated (*).

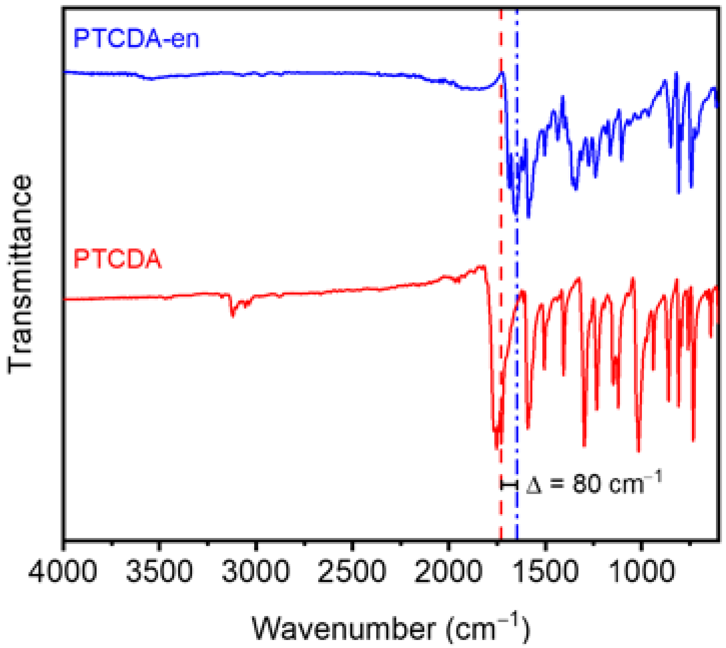

Figure S47. ATR-IR spectra for **PTCDA** and **PTCDA-en**. The shift in the carbonyl stretching frequency between the dianhydride and the diimide is indicated.

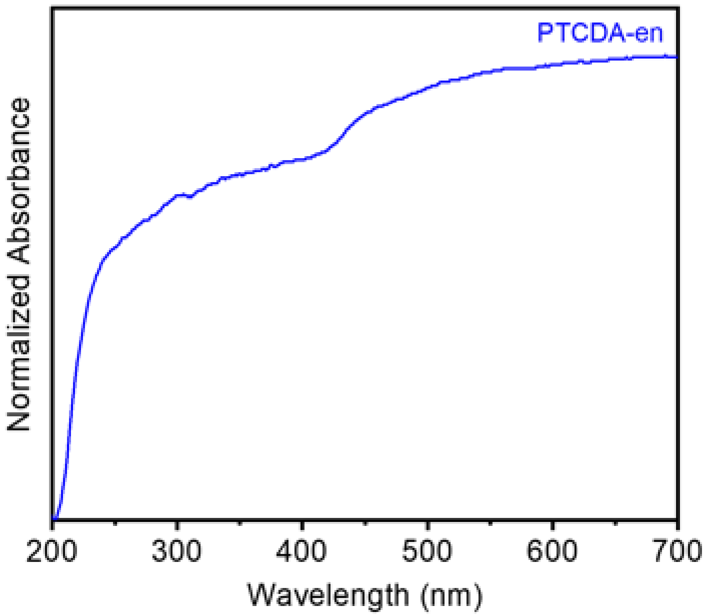

Figure S48. Solid-state UV-Vis absorption spectrum of **PTCDA-en**.

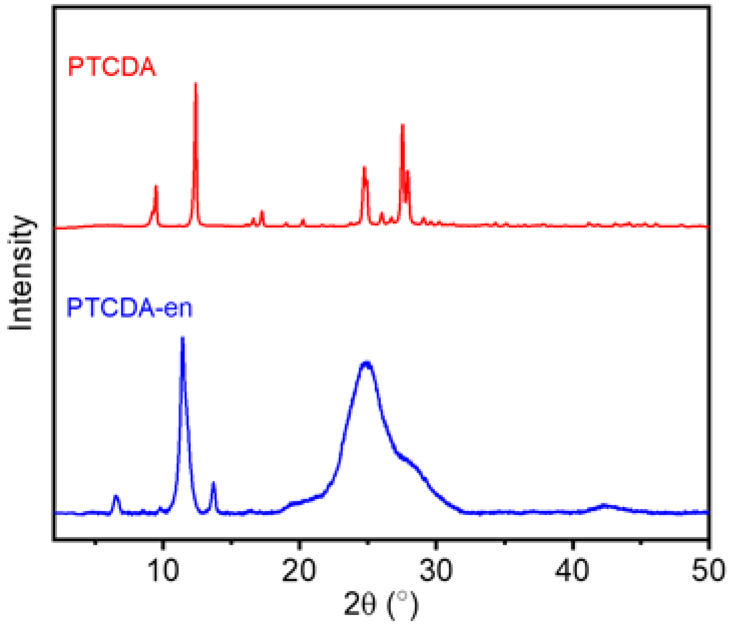

Figure S49. PXRD patterns (λ = 1.54 Å) of **PTCDA** and **PTCDA-en**. These patterns indicate that **PTCDA-en** is a microcrystalline polymer and is likely not contaminated with **PTCDA**. Note that **en** is a liquid at STP.

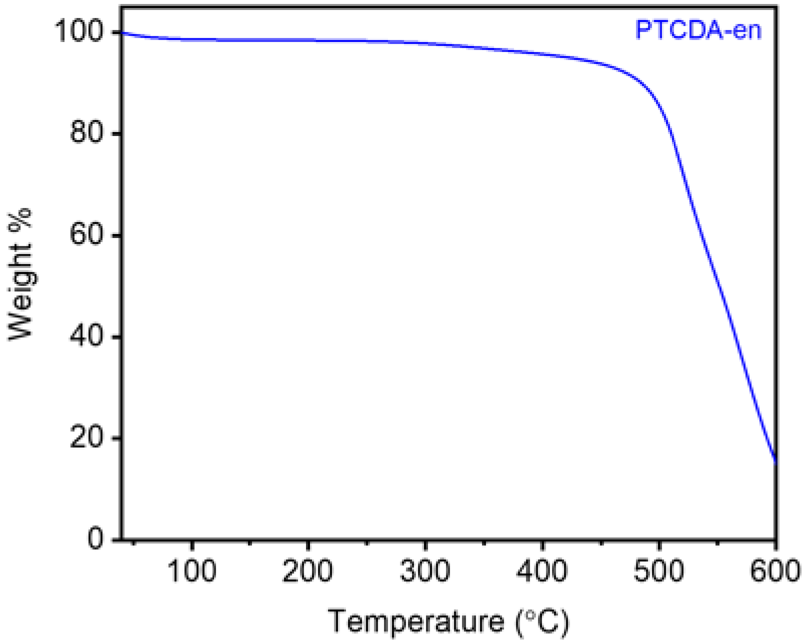

Figure S50. Thermogravimetric decomposition profile in air for **PTCDA-en**, indicating that it is stable below 450 °C.

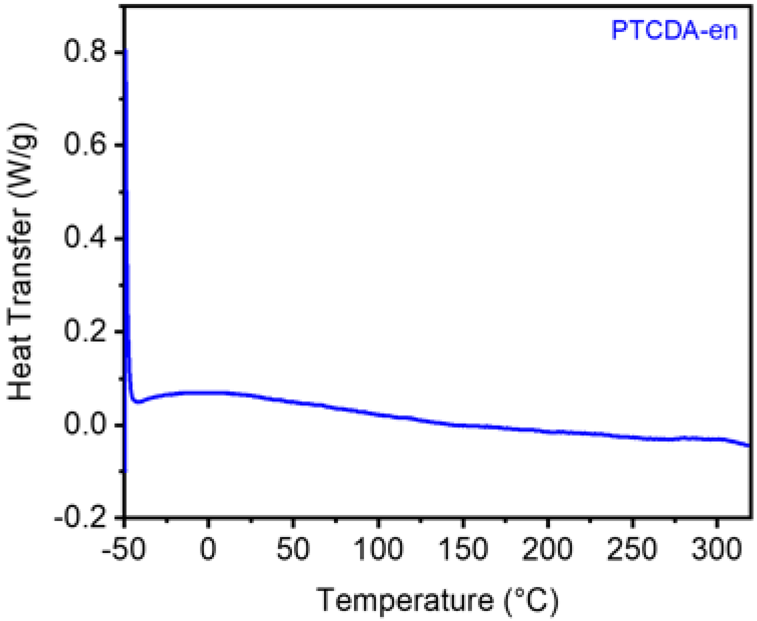

Figure S51. DSC profile under N_2 for **PTCDA-en**, showing the second heating curve from −50 °C to 320 °C. No significant phase changes were observed for this material in this temperature range.

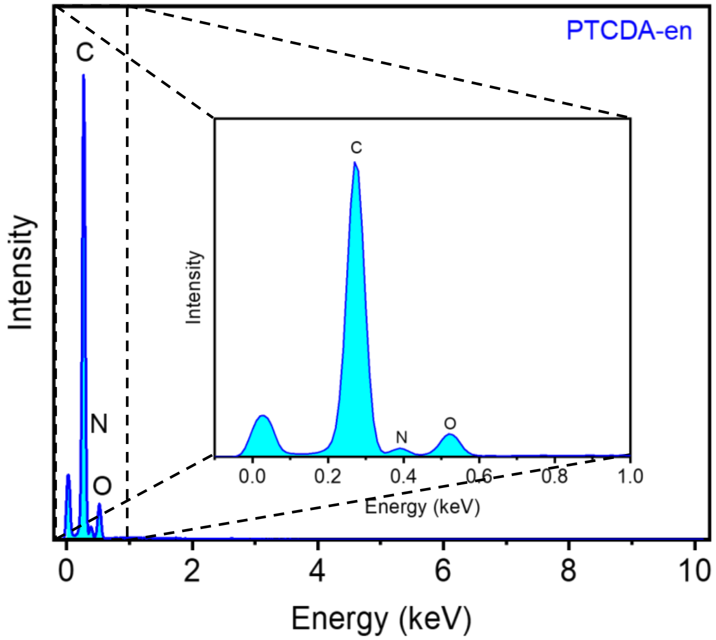

Figure S52. EDS spectrum of **PTCDA-en**.

Table S9. Tabulated EDS data for **PTCDA-en**.

Element	Line Type	Wt %	Atomic %	Theoretical Atomic %[1]
C	K series	73.64	77.71	81.25
O	K series	13.95	11.23	12.50
N	K series	12.41	11.06	6.25
Total:		100.00	100.00	100.00

[1]Excludes hydrogen.

Table S10. Tabulated combustion analysis data for **PTCDA-en**.

Element	Wt %	Theoretical Wt %
C	70.74	75.00
O	16.66[1]	15.37
H	3.08	2.90
N	9.52	6.37
Total:	100.00	100.00

[1]The remaining mass not attributed to C, H, and N was assumed to come from O, as O was not directly analyzed during combustion analysis.

4. Electrochemical experiments.

a. Solution CV/RDE experiments.

For the electrochemical evaluation of the molecular analogues (**PMDA-di^{i}PrAn**, **NTCDA-di^{i}PrAn**, and **PTCDA-di^{i}PrAn**), each of the small molecules was prepared as a 1 mM stock solution in anhydrous DMF. The solution was then filtered to remove any undissolved material and divided into four separate vials. To each vial, a different perchlorate salt was added (TBAP, $KClO_4$, $NaClO_4$, or $LiClO_4$) at 0.1 M concentration. The solutions were then added into a three-compartment cell, where the outer compartments contained blank electrolyte solution (no analyte species). The solution was thoroughly degassed with Ar to remove any dissolved oxygen. For the electrochemical measurements, a 5 mm glassy carbon electrode was used as the working electrode, a coiled Pt wire was used as the counter electrode, and a house-made Ag/AgCl (3 M KCl solution) electrode was used as the reference. Cyclic voltammetry (CV) and rotating disk electrode (RDE) experiments were carried out to characterize each solution. Following the measurements, sublimed ferrocene was added to the solution to serve as an internal reference and to correct for solution resistance. The potential scales for the figures presented in the manuscript were then converted to estimated potentials vs. SHE (Fc/Fc^+ = 0.400 V vs. SHE).

b. Coin cell fabrication.

For electrochemical measurements performed in the solid state, the polymeric materials were incorporated into 2032 coin cells. First, the active material, Super P carbon (Imerys Graphite & Carbon, dried in vacuum oven at 60 °C overnight), and PVDF (Kynar Flex, dried in vacuum oven at 60 °C overnight), were mixed at a ratio of 6:3:1 (by wt) in NMP. The resulting slurry was then spread on a carbon paper current collector using the doctor blade method and dried in a vacuum oven at 60 °C for 2 h and then at 110 °C overnight. The electrode was then punched into disks (3/16'' diameter for CV tests, 1/4'' for activation energy measurements, and 3/8'' for all other electrochemical tests) with an average active mass loading of ~1 mg/cm^2. The disks were used as the cathode within the assembled coin cells. The cells were assembled with a fiber glass separator, a 1/2'' disk metal anode, and 80 μL of an electrolyte solution consisting of the corresponding metal ion PF_6^- salt (1 M and 0.8 M $LiPF_6$ for Li batteries, 1 M $NaPF_6$ for Na batteries, and 0.8 M KPF_6 for K batteries) in EC:DEC (1:1 v:v). Cell assembly was conducted in an Ar-filled glovebox with O_2 levels below 0.5 ppm.

For Li batteries, lithium metal foil was used as received. For Na batteries, Na metal cubes were made into a foil by first removing the oxide layer with a ceramic knife. The Na metal was then placed in a plastic bag and pressed flat using a pasta maker. The resulting foil was then punched into disks. For K batteries, K metal chunks were made into a foil by first removing the oxide layer with a ceramic knife. The K metal was then placed in a plastic bag which had been washed with acetone and then rolled flat with a pasta maker. The resulting foil was then punched into disks.

Solid polymer electrolyte cells were made by dissolving LiTFSI and polyethylene oxide (PEO, average M_V 600,000) in MeCN and stirring the resulting solution for 24 h. The ratio of Li^+

to ethylene oxide units was 0.01. Following mixing, the MeCN was allowed to evaporate at room temperature until a thick, gel-like solution was obtained. The solution was then dropped onto Celgard separators. The separators were then dried in a vacuum oven at 60 °C overnight, and the resulting solid polymer electrolyte was stored in the glovebox until use.

The resulting batteries were examined using galvanostatic charge/discharge, CV, and potentiostatic electrochemical impedance spectroscopy (PEIS). Galvanostatic charge/discharge measurements were performed using a Neware battery test station. CV and PEIS measurements were performed with a Biologic SP150 potentiostat.

c. Cyclic voltammetry studies to determine diffusion-limitations.

CV experiments were performed at slow scan rates (1.00, 0.75, 0.50, 0.25, and 0.10 mV/s) to determine the kinetic processes limiting the current response throughout the charge/discharge process. Because surface-controlled kinetics exhibit a current i that scales with the scan rate v (*i.e.*, $i \propto v$), while diffusion-limited kinetics have a current that scales with $v^{1/2}$, the following relationship (eq. S1) can be used over a range of potentials employed in CV experiments to determine the contribution of each to the total current:

$$i(V) = k_1 v + k_2 v^{1/2} \qquad \text{eq. S1}$$

where k_1 is an experimentally determined constant related to the surface-limited current contribution, and k_2 is an experimentally determined constant relating to the diffusion-limited current. Integration of the diffusion-limited current term relative to the total integrated current provides the percentage of the charge arising from diffusion-limited processes.

d. Activation energy measurements.

For activation energy measurements, three-electrode coin cells were employed and assembled following a procedure described in the literature.[6] Briefly, a third electrode was incorporated into the cell to serve as a reference electrode. The reference electrode consisted of a stainless-steel strip with one end embedded in a small piece of Li foil. The reference electrode was placed between the working electrode (polymer slurry) and the counter electrode (Li metal disk). To prevent contact between the reference, counter, and working electrode, separators were placed above and beneath the reference electrode. Additionally, Kapton tape was place on the cell wall and base, under the stainless-steel strip, to prevent contact between the working electrode and reference electrode at the cell edge.

For activation energy measurements, the cells were first pre-cycled 5 times at 1 mV/s by CV. PEIS measurements were then performed at the $E_{1/2}$ of the first redox couple (between the neutral and −1 state) of each polymer. Before PEIS measurements were conducted, the cell was allowed to equilibrate for 1 h at a given temperature while being held at the potential used in the PEIS measurements. A potential amplitude of 5 mV was employed and a frequency range of 5 MHz to 50 mHz was used. The high frequency semi-circle portion of the Nyquist plot was fit with

a modified Randles-circuit (R_s + (Q_1/R_{ct}) where R_s is solution resistance, Q_1 is a constant phase element, and R_{ct} is the charge transfer resistance) using ZFit. From the fits, the R_{ct} was extracted for the calculation of the activation energy.

The PEIS measurements were conducted for each cell at four different temperatures to generate an Arrhenius plot from the measured charge transfer resistance (ln(1/R_{ct}) vs. 1/T, where T is temperature in Kelvin. From the slope of the obtained line, the activation energy, E_a, was calculated using eq. S2:

$$slope = -\frac{E_a}{R} \quad \text{Eq. S2}$$

where R is the universal gas constant.

e. Conductivity measurements.

Pressed-pellet conductivities were measured using a two-point probe. The sample powder was placed between two stainless-steel screws and two copper contacts encased in a Teflon tube, such that the entire contact area (A, 0.0919 cm^2) was covered by the powder. A normal pulse voltammogram was carried out with a Gamry Instruments Interface 1010e potentiostat, where pulses of 100–500 mV were applied across the device, and the current responses were used to generate I-V plots. A Fluke 75III multimeter was used in cases where a reliable current response could not be obtained due to the high resistance of the material (**PTCDA-en**) even with thinner pressed pellets. The slope of the plot corresponded to the resistance, R, of the sample, and the following equation was used to calculate the conductivity (σ):

$$\sigma = \frac{t}{R \times A} \quad \text{Eq. S3}$$

where t is the thickness of the layer of material between the two metal contacts.

f. Supporting figures.

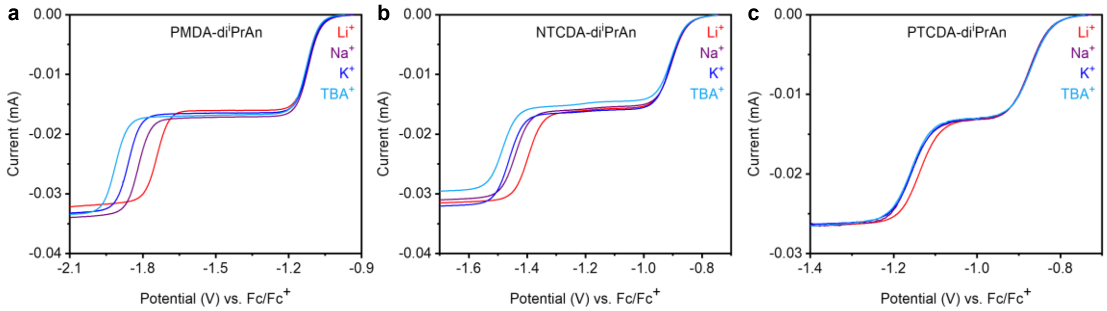

Figure S53. RDE voltammetry profiles run at 5 mV/s and 65 rpm of solutions of 0.1 M perchlorate salts in DMF containing 1 mM (a) **PMDA-di^{i}PrAn**, (b) **NTCDA-di^{i}PrAn,** and (c) **PTCDA-di^{i}PrAn**.

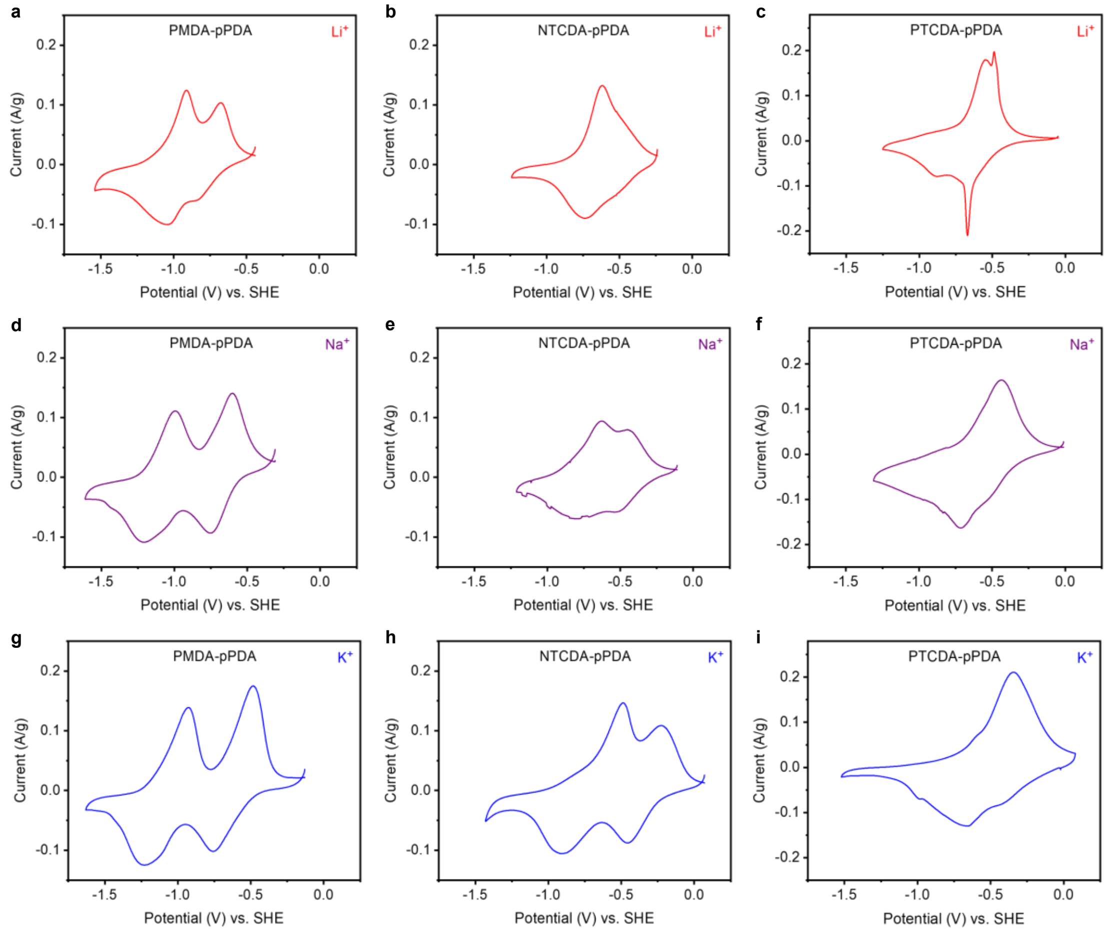

Figure S54 Cyclic voltammograms of (a) **PMDA-pPDA**, (b) **NTCDA-pPDA**, and (c) **PTCDA-pPDA** in Li half cells, (d) **PMDA-pPDA**, (e) **NTCDA-pPDA**, and (f) **PTCDA-pPDA** in Na half cells, and (g) **PMDA-pPDA**, (h) **NTCDA-pPDA**, and (i) **PTCDA-pPDA** in K half cells. All CV experiments were obtained from coin cells at 0.25 mV s^{-1}. The potential scales have been modified from their corresponding M/M^+ (where M is Li, Na, or K) potential scales to potentials vs. SHE based on tabulated standard reduction values (Li/Li^+ = −3.05 V vs. SHE; Na/Na^+ = −2.71 V vs. SHE; K/K^+ = −2.92 V vs. SHE).

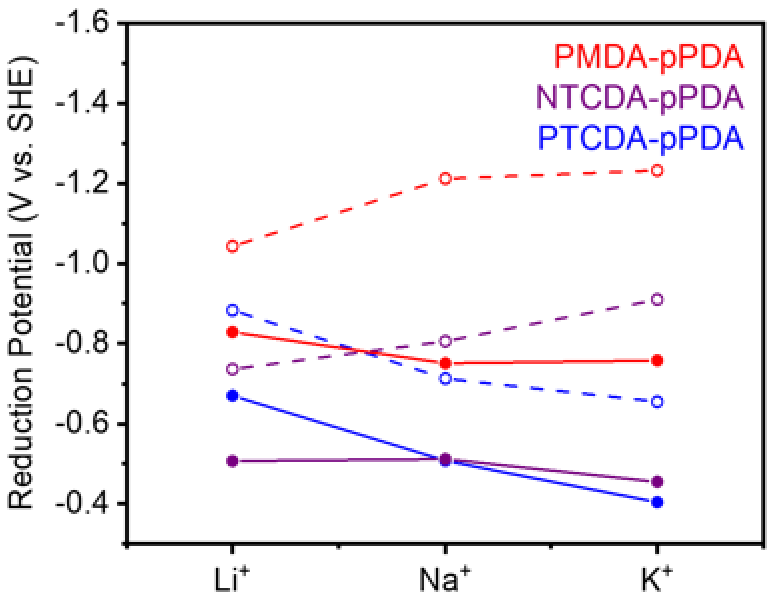

Figure S55. Reduction peak potentials from cyclic voltammograms of coin cells at 0.25 mV s^{-1} in **Figure S54**. The solid lines/filled circles correspond to the first reduction peak potential (0 → −1) and the dotted lines/open circles correspond to the second reduction peak potential (−1 → −2).

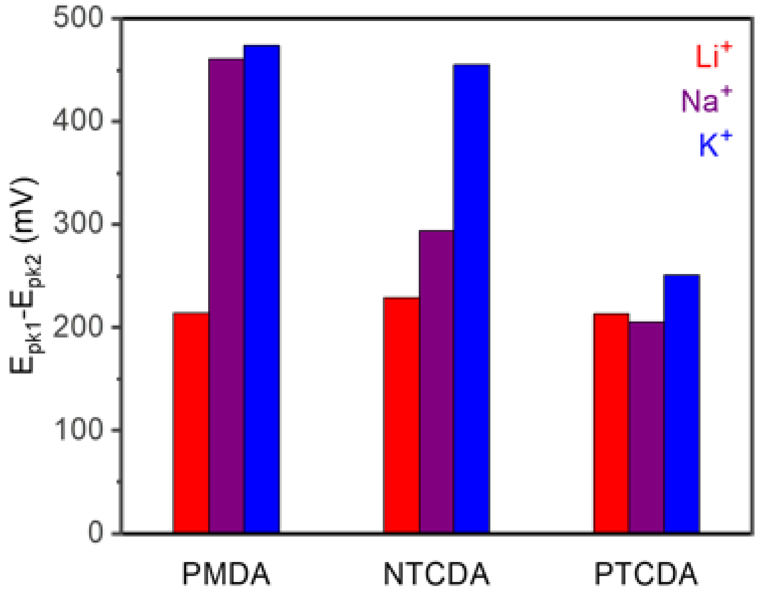

Figure S56. Difference in the reduction peak potential values between the first reduction (0 → −1) and second reduction (−1 → −2) from the cyclic voltammograms in **Figure S54** as a function of polymer and charge compensating ion of the **pPDA**-based polymers. The x-axis is labelled by the tetracarboxylic acid dianhydride used in the synthesis of the polymer.

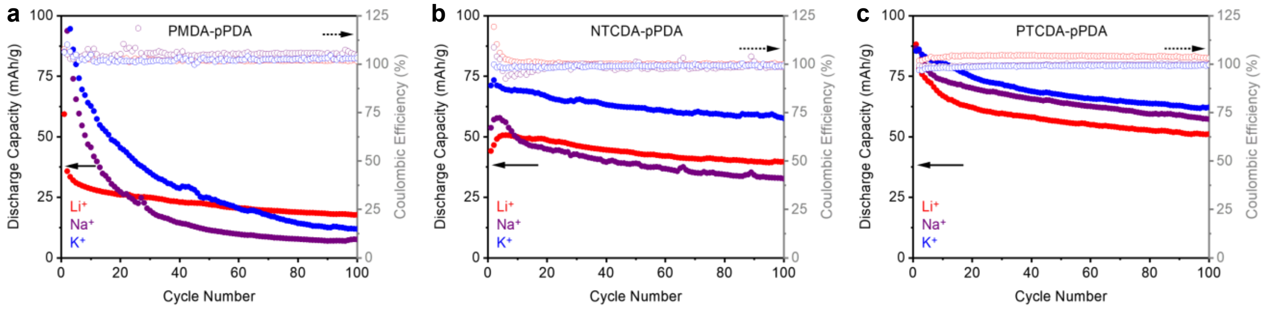

Figure S57. Cycling tests at 100 mA g^{-1} of (a) **PMDA-pPDA**, (b) **NTCDA-pPDA**, and (c) **PTCDA-pPDA** in Li, Na, and K cells. Coulombic efficiencies are shown by the open circles and the values are represented on the secondary axis.

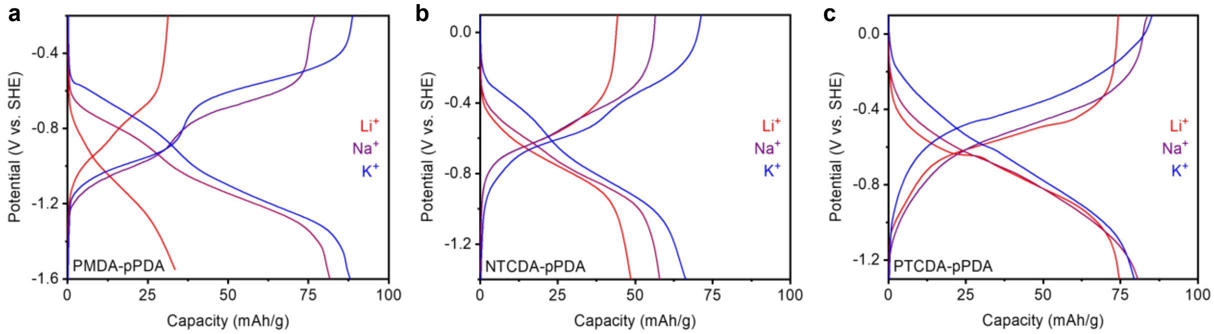

Figure S58. Charge/discharge curves at 100 mA g^{-1} of (a) **PMDA-pPDA**, (b) **NTCDA-pPDA**, and (c) **PTCDA-pPDA** where the potentials have been modified from their corresponding M/M^+ (where M is Li, Na, or K) potential scales to potentials vs. SHE based on tabulated standard reduction values.

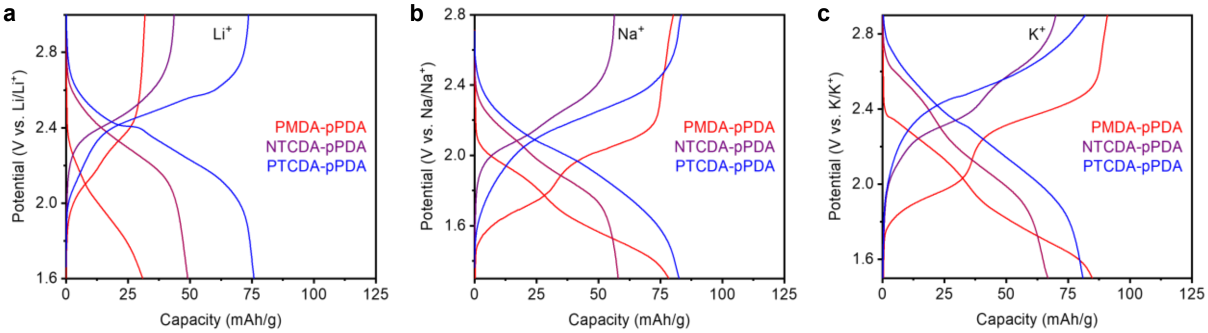

Figure S59. Charge/discharge curves at 100 mA g^{-1} of **PMDA-pPDA**, **NTCDA-pPDA**, and **PTCDA-pPDA** in (a) Li half cells, (b) Na half cells, and (c) K half cells.

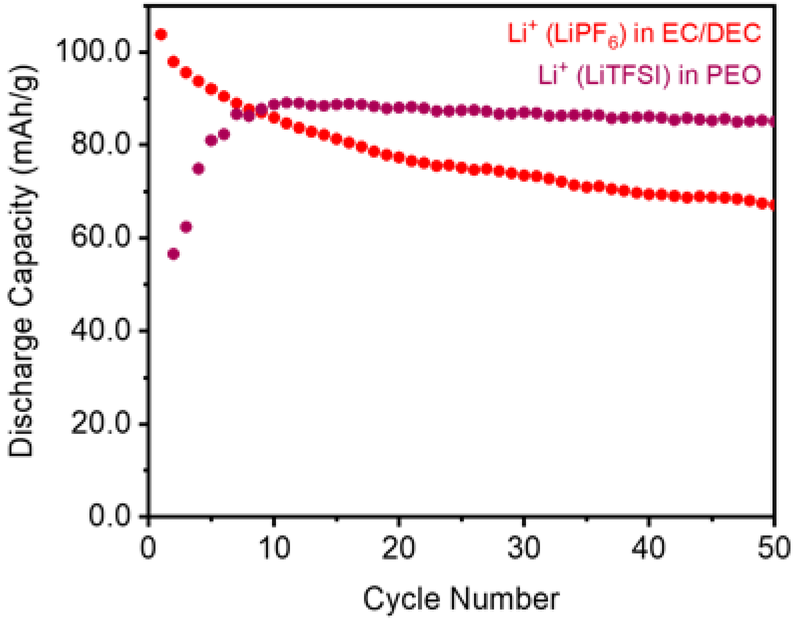

Figure S60. Cycling performance of **PTCDA-pPDA** at 100 mA g^{-1} in a coin cell in which the electrolyte solution is 1 M $LiPF_6$ in EC:DEC (red, liquid electrolyte) or LiTFSI in a PEO (maroon, solid polymer electrolyte) where r = 0.01 (where r is the ratio of Li^+ to ethylene oxide moieties). The cycling tests with the solid polymer electrolyte were performed at 60 °C such that the electrolyte would be in the amorphous phase. The high cycling stability observed with the polymer electrolyte implies that the poor cycling stability of **PTCDA-pPDA** with $LiPF_6$ in EC:DEC is likely due to dissolution of the polymer electrode material into the liquid electrolyte.

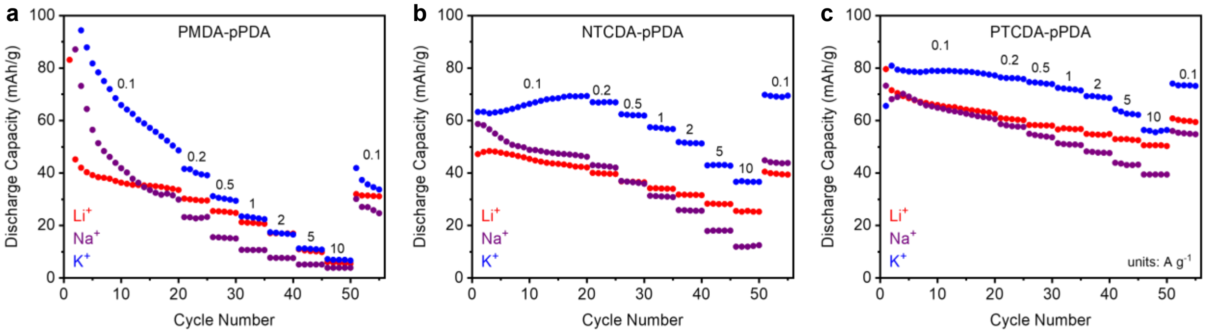

Figure S61. Representative rate tests of (a) **PMDA-pPDA**, (b) **NTCDA-pPDA**, and (c) **PTCDA-pPDA** in Li, Na, and K cells. Cells were first cycled 20 times at 0.1 A g^{-1} before being subjected to higher rates of discharge. The discharge rates are represented on the plot in units of A g^{-1}. The rate of charge was held constant at 0.1 A g^{-1} throughout the experiments.

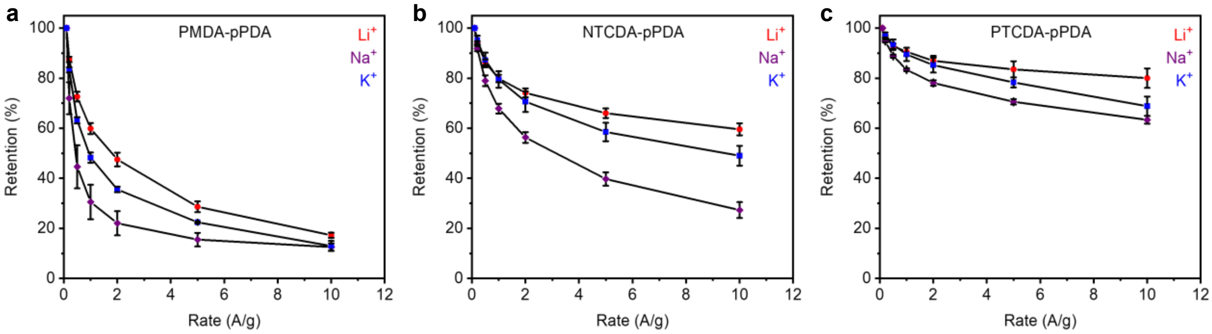

Figure S62. Capacity retention at different discharge rates compared to the 20th cycle at 100 mA g^{-1} for (a) **PMDA-pPDA**, (b) **NTCDA-pPDA**, and (c) **PTCDA-pPDA** in the presence of Li^+, Na^+, or K^+.

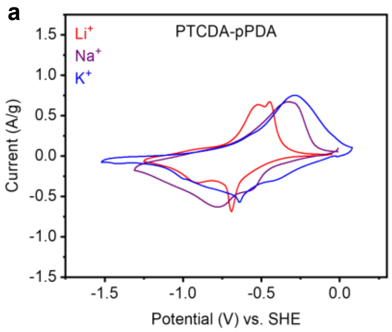

Figure S63. Cyclic voltammograms at 1 mV s^{-1} of (a) **PTCDA-pPDA**, (b) **PTCDA-chex**, and (c) **PTCDA-en** where the potentials have been modified from their corresponding M/M^+ (where M is Li, Na, or K) potential scales to potentials vs. SHE based on tabulated standard reduction values.

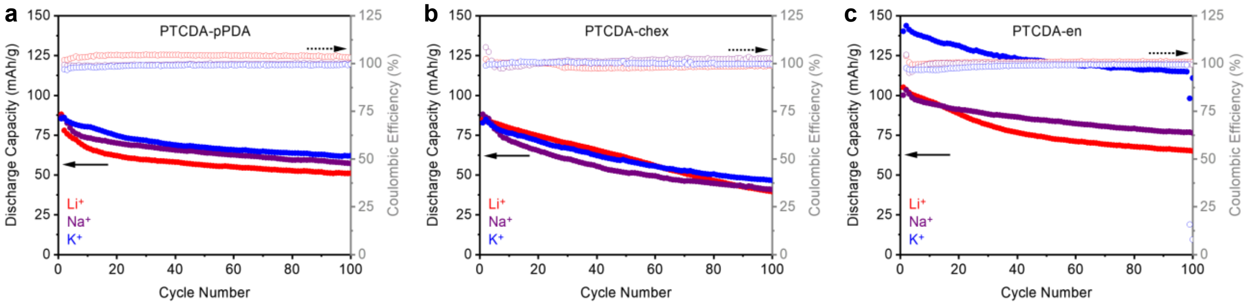

Figure S64. Cycling tests at 100 mA g^{-1} of (a) **PTCDA-pPDA**, (b) **PTCDA-chex,** and (c) **PTCDA-en** in Li, Na, and K cells. Coulombic efficiencies are represented by the open circles.

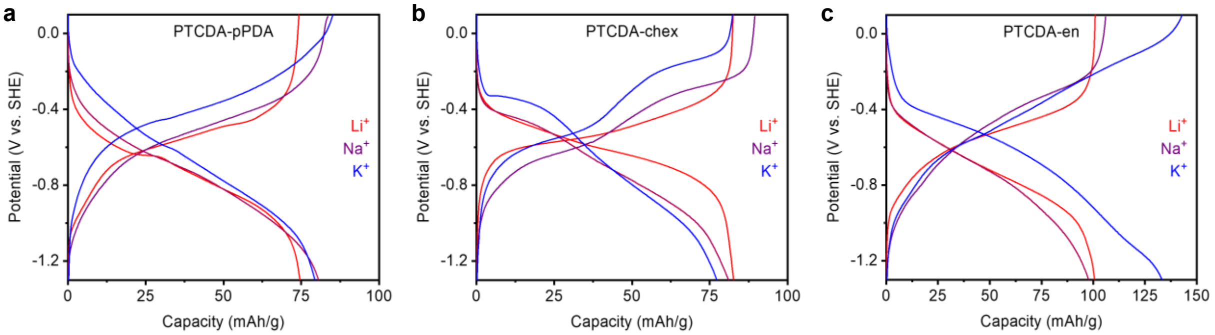

Figure S65. Charge/discharge curves at 100 mA g^{-1} of (a) **PTCDA-pPDA**, (b) **PTCDA-chex**, and (c) **PTCDA-en** where the potentials have been modified from their corresponding M/M^+ (where M is Li, Na, or K) potential scales to potentials vs. SHE based on tabulated standard reduction values.

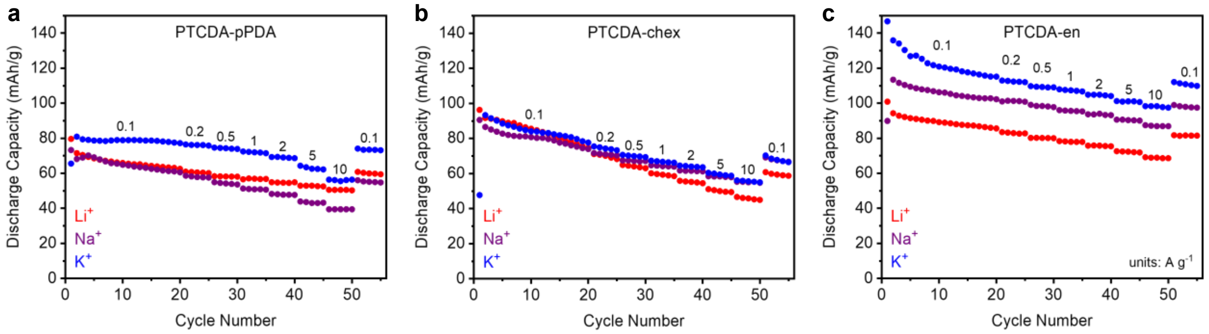

Figure S66. Representative rate tests of (a) **PTCDA-pPDA**, (b) **PTCDA-chex**, and (c) **PTCDA-en** in Li, Na, and K cells. Cells were first cycled 20 times at 0.1 A g^{-1} before being subjected to higher rates of discharge. The discharge rates are represented on the plot in units of A g^{-1}. The rate of charge was held constant at 0.1 A g^{-1} throughout the test.

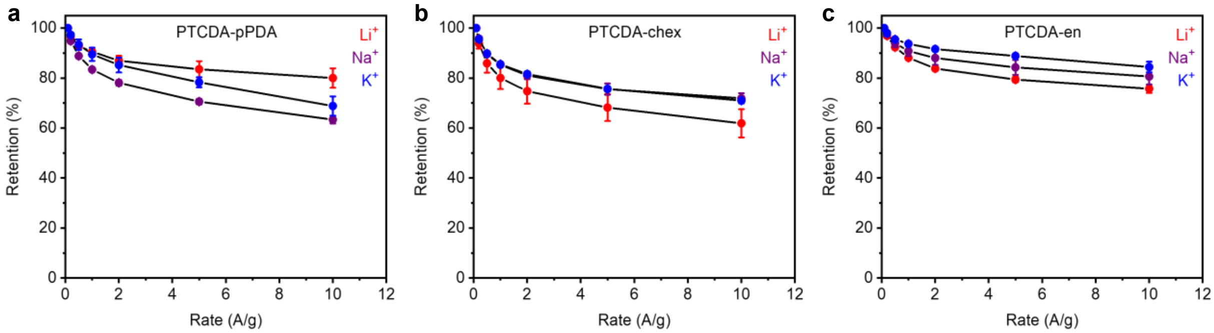

Figure S67. Capacity retention at high rates of discharge relative to the 20th cycle capacity at 0.1 A g^{-1} for (a) **PTCDA-pPDA**, (b) **PTCDA-chex,** and (c**) PTCDA-en**. Errors bars represent one standard deviation in the capacity, based on data from at least three coin cells.

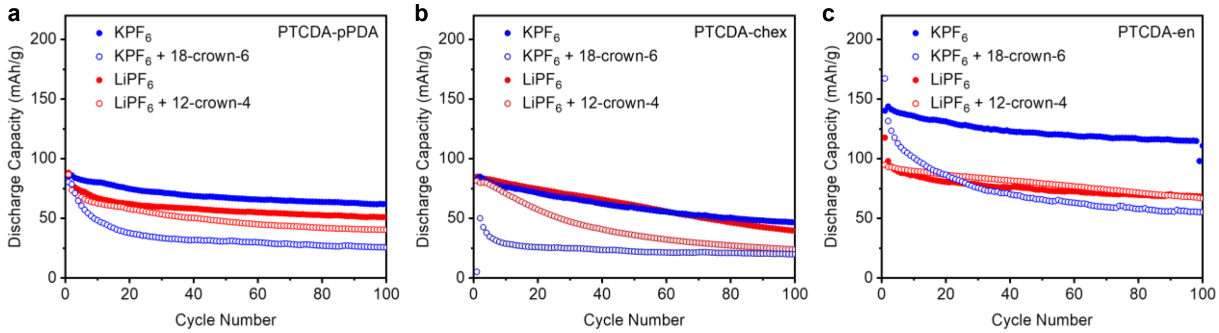

Figure S68. Extended cycling performance at 100 mA g^{-1} for (a) **PTCDA-pPDA**, (b) **PTCDA-chex**, and (c) **PTCDA-en** in a solution of EC/DEC with 0.8 M KPF_6 with (open blue circles) and without (closed blue circles) 0.8 M 18-crown-6 ether added and with 1 M $LiPF_6$ with (open red circles) and without (closed red circles) 1 M 12-crown-4 ether added.

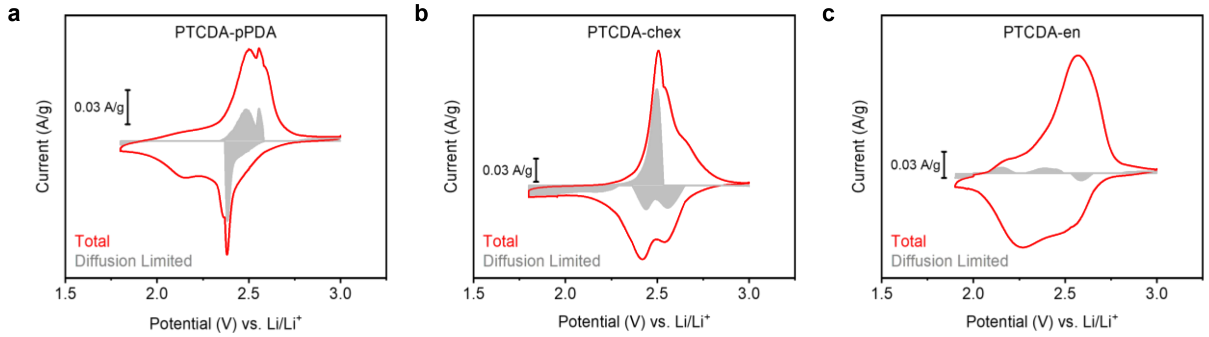

Figure S69. Cyclic voltammograms of Li^+ cells in which the red line represents the total current and the grey region represents current arising from diffusion limited processes, calculated from eq. S1, for (a) **PTCDA-pPDA**, (b) **PTCDA-chex**, and (c) **PTCDA-en**.

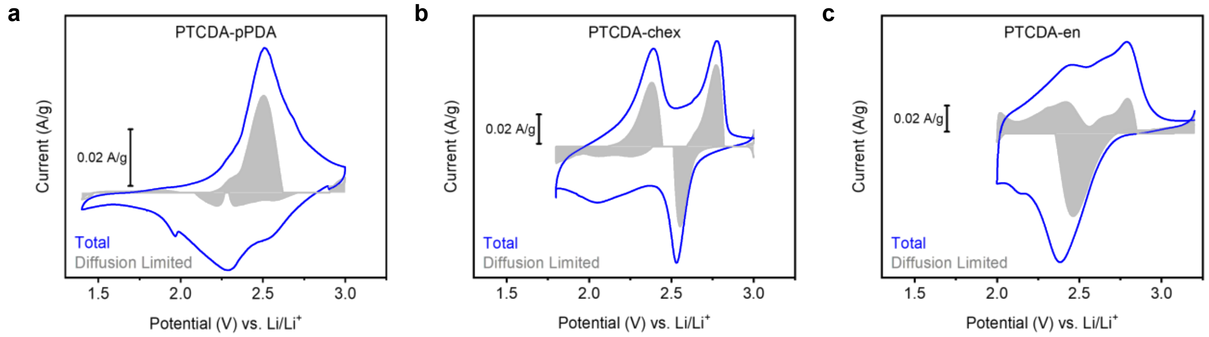

Figure S70. Cyclic voltammograms of K^+ cells in which the blue line represents the total current and the grey region represents current arising from diffusion limited processes, calculated from eq. S1, for (a) **PTCDA-pPDA**, (b) **PTCDA-chex**, and (c) **PTCDA-en**.

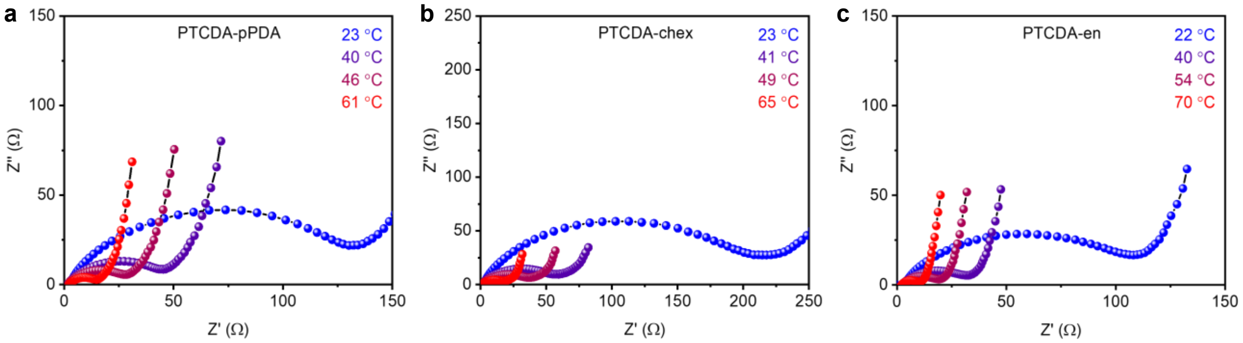

Figure S71. Nyquist plots from PEIS measurements conducted in 3-electrode Li^+ coin cells with (a) **PTCDA-pPDA**, (b) **PTCDA-chex**, and (c) **PTCDA-en**. The semi-circle region of the Nyquist plot was fit and a value for R_{ct} was extracted at each temperature.

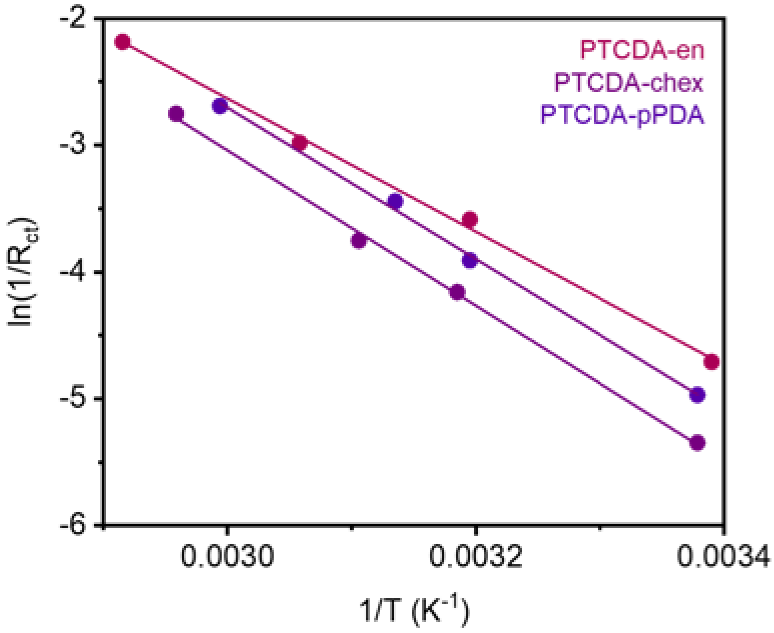

Figure S72. Arrhenius plots constructed using the R_{ct} values obtained from the PEIS measurements in **Figure S71**. The slopes of the plots were used to determine the activation energy of charge transfer in the **PTCDA**-based polymers. These measurements were repeated at least three times for each polymer to determine the error associated with the activation energies calculated from this measurement.

Table S11. Calculated activation energies of charge transfer for the **PTCDA**-based polymers.

Polymer	Activation Energy (kJ/mol)
**PTCDA-pPDA**	51.1 ± 1.6
**PTCDA-chex**	50.5 ± 0.4
**PTCDA-en**	42.5 ± 1.0

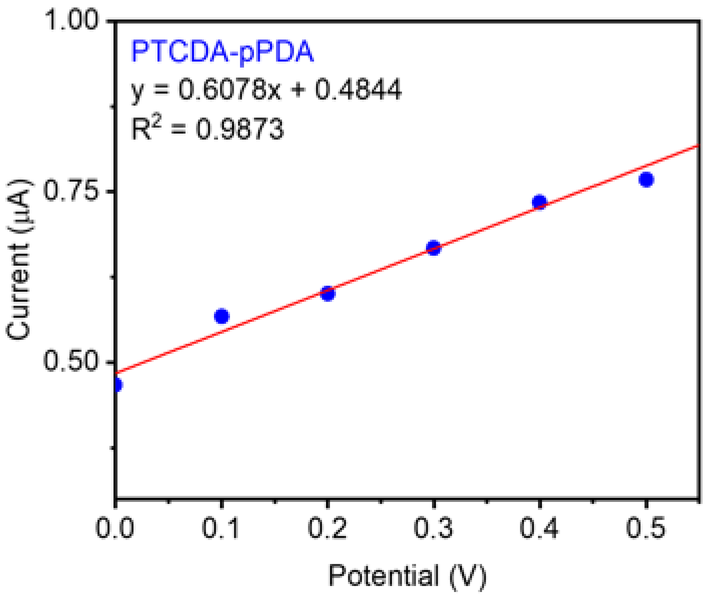

Figure S73. Two-point probe conductivity measurement of **PTCDA-pPDA** indicating that this material displays Ohmic conductor behavior with a resistance of 1650000 Ω. This results in a conductivity of 2.45×10^{-7} S/cm for a powder sample with a thickness of 0.37 mm.

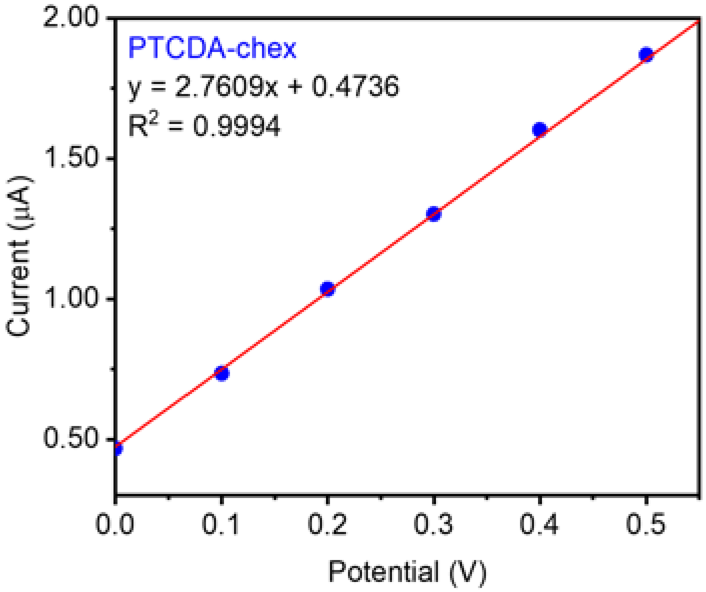

Figure S74. Two-point probe conductivity measurement of **PTCDA-chex** indicating that this material displays Ohmic conductor behavior with a resistance of 362000 Ω. This results in a conductivity of 1.6×10^{-6} S/cm for a powder sample with a thickness of 0.52 mm.

The resistance of **PTCDA-en** (7040000 Ω) was directly measured with a multimeter, resulting in a conductivity of 4.0×10^{-8} S/cm for a powder sample thickness of 0.26 mm.

g. Additional electrochemical experiments with changed parameters and solubility tests.

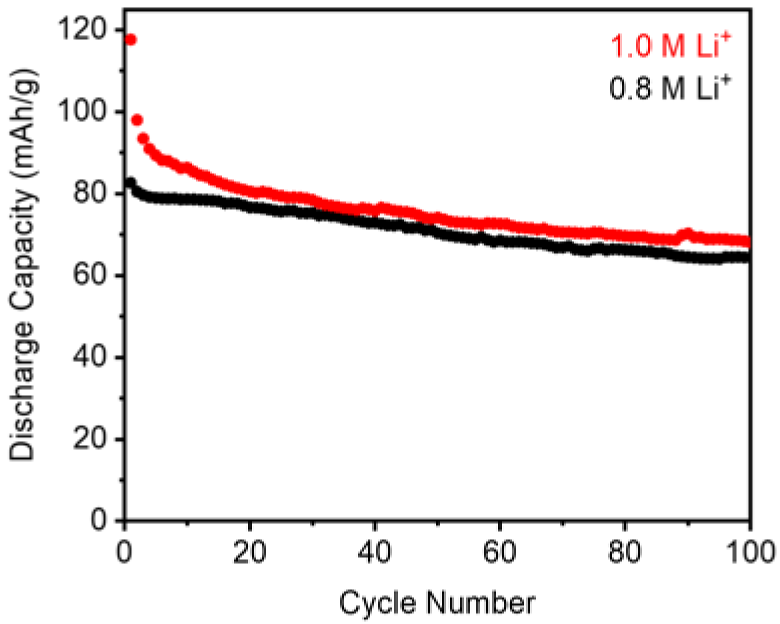

Figure S75. Cycling tests at 100 mA g^{-1} of **PTCDA-en** in 1.0 M and 0.8 M $LiPF_6$ in EC:DEC, indicating that the behavior of the polymer did not change significantly with the decrease in concentration from 1.0 M to 0.8 M $LiPF_6$.

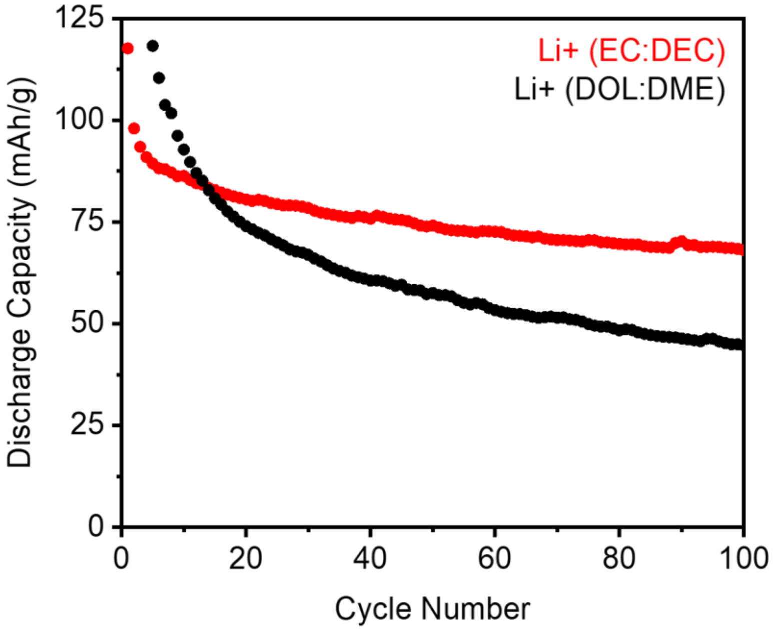

Figure S76. Cycling tests of **PTCDA-en** at 100 mA g^{-1} in 1 M $LiPF_6$ in EC:DEC (red) and 1 M LiFSI in DOL:DME (black). When cycled in the electrolyte containing DOL:DME, **PTCDA-en** delivers a higher capacity upon initial cycling as would be expected in a lower dielectric solvent where the Li^+ would interact less strongly with the solvent molecules. However, a lower cycling stability is also observed, likely due to the reduced stabilizing effects in a lower dielectric solvent.

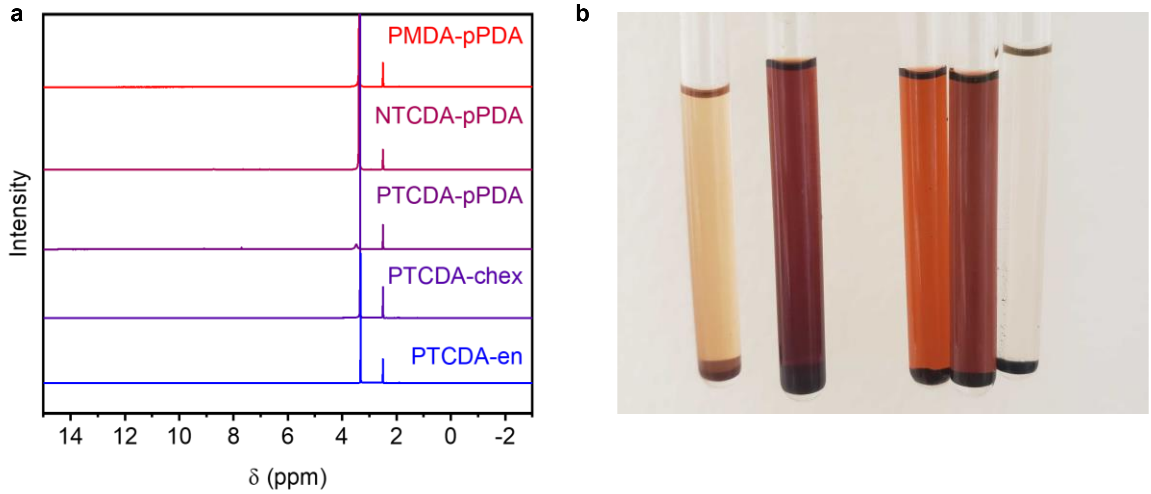

Figure S77. Solubility of the examined polymers through (a) ^{1}H-NMR spectra (500 MHz, DMSO-d_6) and (b) settling of the solids in the NMR samples (from left to right: **PMDA-pDPA**, **NTCDA-pPDA**, **PTCDA-pDPA**, **PTCDA-chex** and **PTCDA-en)**, indicating all of the polymers are only sparingly soluble (likely only short chain oligomers) in DMSO.

5. Structural comparison and surface area measurements for PTCDA-based polymers.

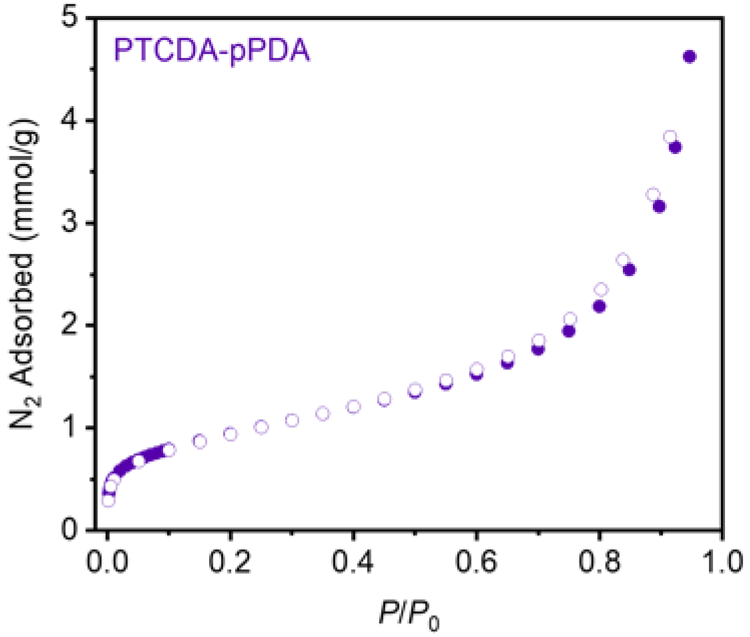

Figure S78. 77 K N_2 adsorption (filled circles) and desorption (open circles) isotherm for **PTCDA-pPDA**. The BET and Langmuir surface areas were determined to be 76 m^2/g and 260 ± 30 m^2/g, respectively.

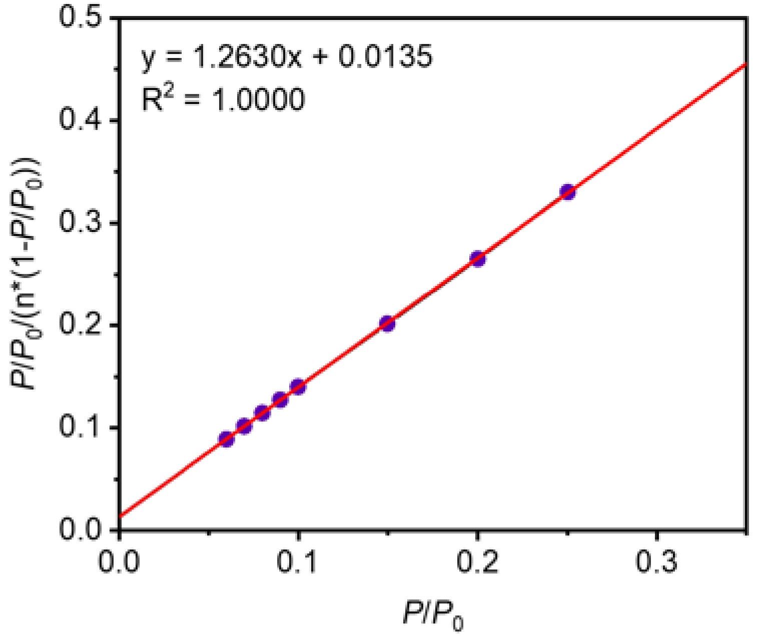

Figure S79. Linearized BET plot of **PTCDA-pPDA**.

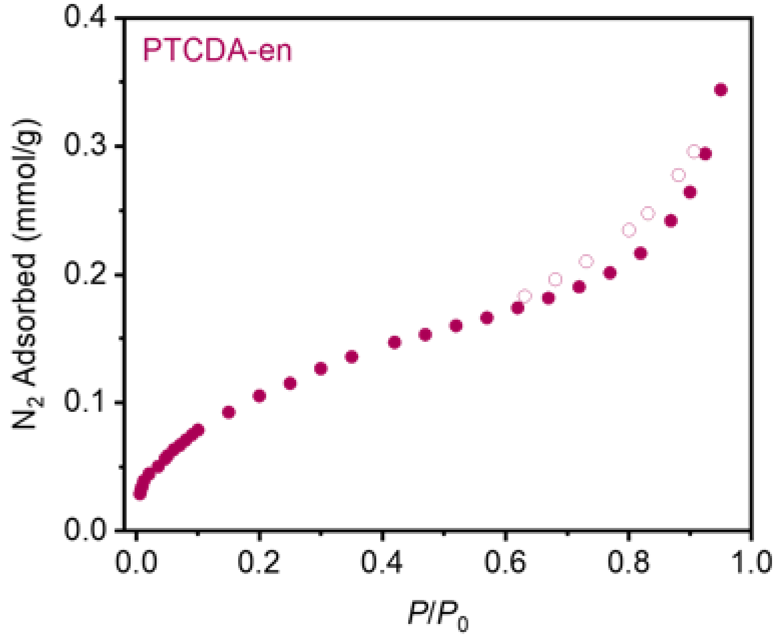

Figure S80. 77 K N_2 adsorption (filled circles) and desorption (open circles) isotherm for **PTCDA-en**. The Brunauer-Emmett-Teller (BET) and Langmuir surface areas were determined to be 10 m^2/g and 27 ± 2 m^2/g, respectively.

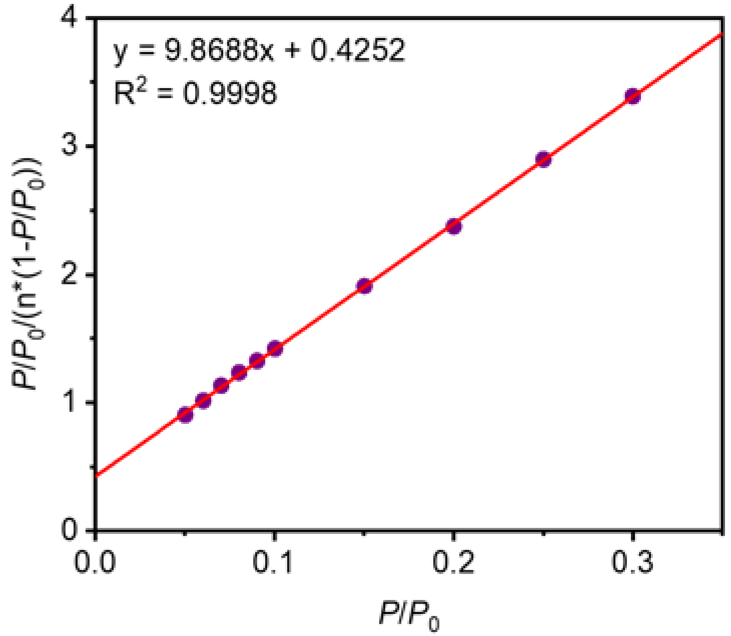

Figure S81. Linearized BET plot of **PTCDA-en**.

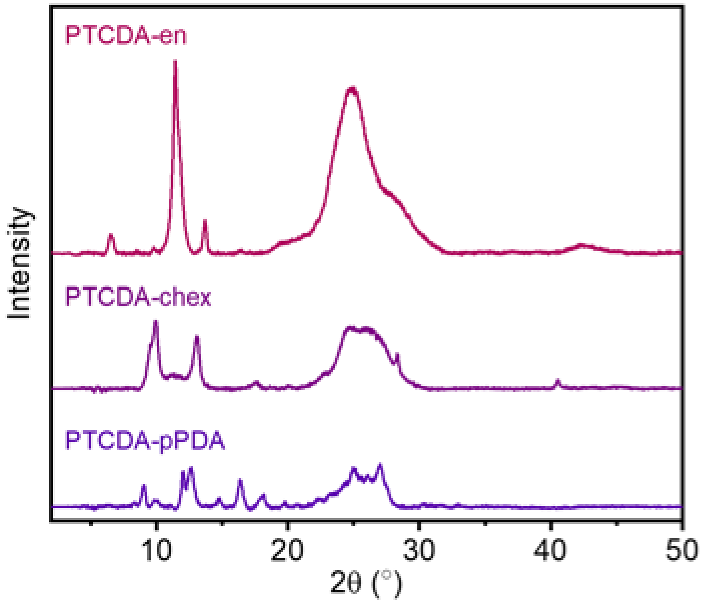

Figure S82. Stacked PXRD patterns of **PTCDA-pPDA**, **PTCDA-chex,** and **PTCDA-en**, showing **PTCDA-pPDA** has the greatest number of sharp reflections in the low angle regime (< 20 2θ). This suggests that **PTCDA-pPDA** has the highest rigidity of the three polymers.

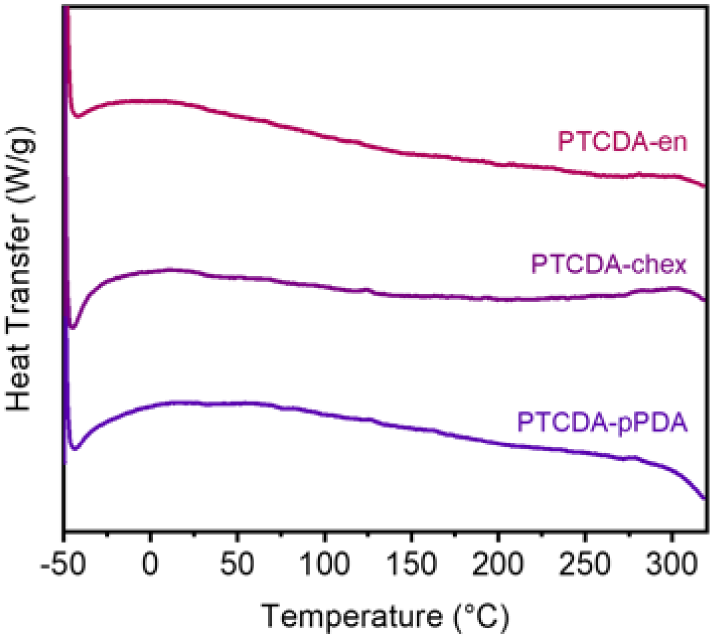

Figure S83. Stacked DSC profiles of **PTCDA-pPDA**, **PTCDA-chex,** and **PTCDA-en**, indicating that none of the polymers have a defined T_g or T_m in the examined temperature range. This finding indicates that any ordering observed in **Figure S82** for the three polymers is relatively short-ranged.

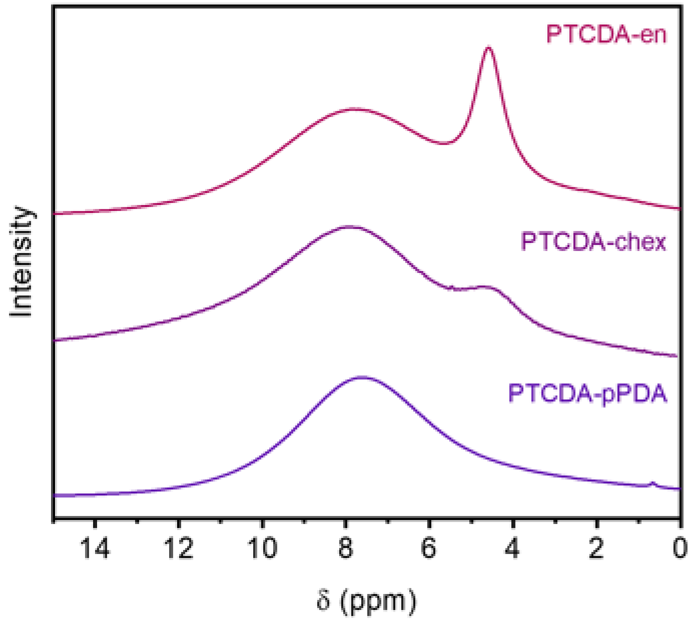

Figure S84. Stacked ^{1}H SSNMR spectra of **PTCDA-pPDA**, **PTCDA-chex,** and **PTCDA-en,** showing **PTCDA-en** has a sharp aliphatic peak while **PTCDA-chex** has a broad aliphatic peak and **PTCDA-pPDA** has no aliphatic peak. This suggests that the aliphatic segments in **PTCDA-en** are flexible and able to dynamically adopt more conformers than those of **PTCDA-chex**.

6. Computational structural models.

Density functional theory (DFT) calculations were carried out at the WB97XD level of theory using Gaussian16.[7,8] The standard Def2-SVP basis set was used for geometry optimizations and the expanded Def2-TZVP basis set was used for single point and population density with natural bonding orbital (NBO) calculations.[9] The calculated structures were then depicted using CYLview20[10].

a. PTCDA-based polymers.

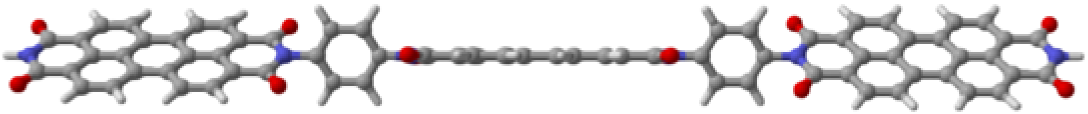

Figure S85. DFT-calculated structure of a portion of **PTCDA-pPDA**, suggesting that the polymer is not fully conjugated.

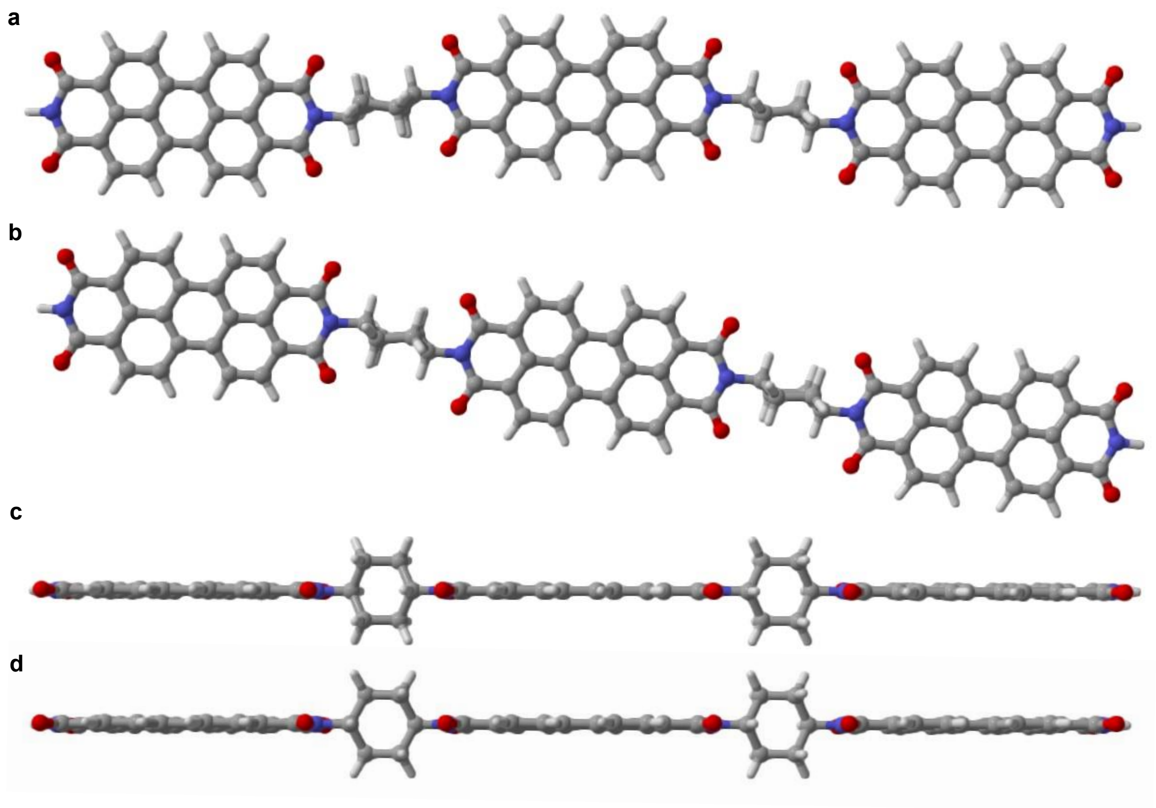

Figure S86. DFT-calculated structures of portions of **PTCDA-chex** with cyclohexane units in (a) alternating and (b) the same orientation calculated with Gaussian16. Structure (a) has a calculated energy that is 0.1 kJ mol^{-1} lower than that of (b), indicating that the two forms have similar thermodynamic stability and would result in straight rigid chains. (c) and (d) show perpendicular views of structures (a) and (b) respectively, indicating that the polyaromatic units are completely planar and perpendicular to the cyclohexane units in both conformations.

.

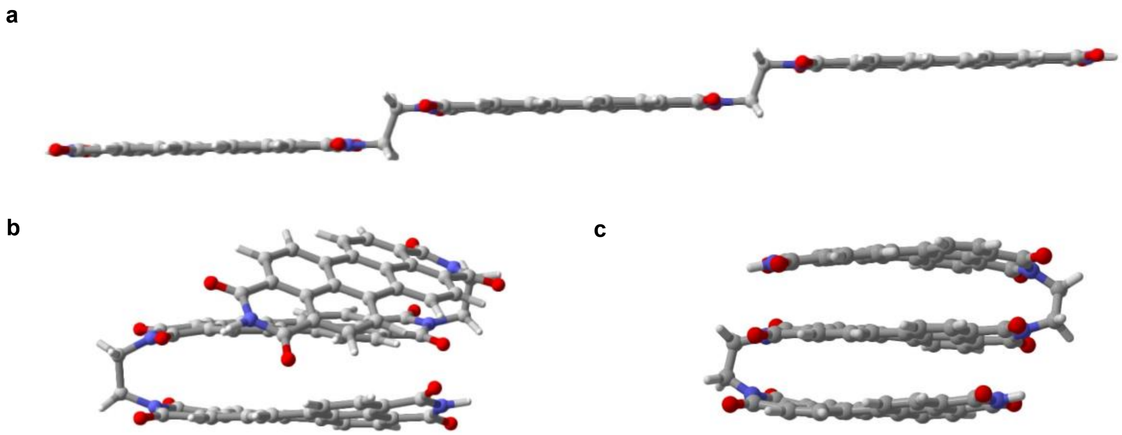

Figure S87. DFT-calculated structures of portions of **PTCDA-en** in which the polymer fragment is (a) completely unfolded, (b) partially folded, and (c) completely folded. Structures (b) and (c) have calculated energies that are approximately 121.6 kJ mol^{-1} and 150.7 kJ mol^{-1} lower than that of the unfolded structure (a), respectively. These calculations suggest multiple energetically stable conformations of **PTCDA-en** strands could be accessible. These calculated structures are consistent with the higher flexibility of **PTCDA-en** compared to the other materials prepared as part of this work.

b. Molecular analogues made with di^{i}PrAn.

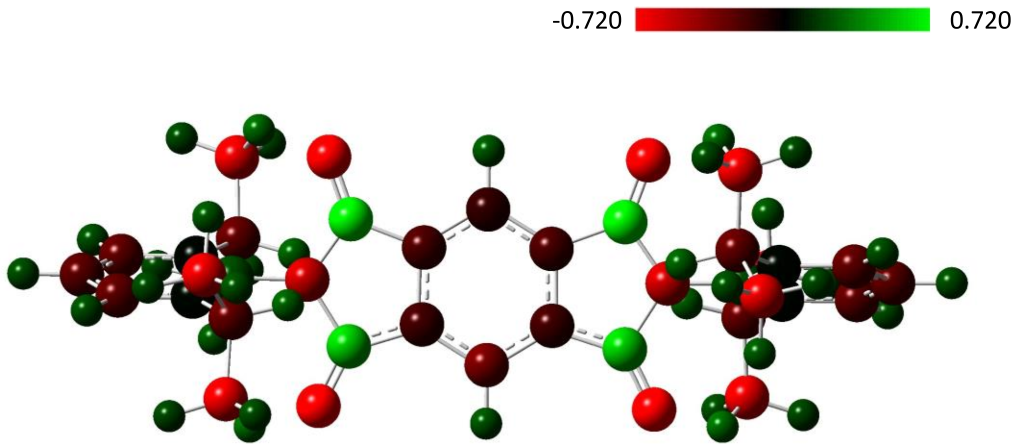

Figure S88. DFT-calculated structure and electron population density distribution of **PMDA-di^{i}PrAn^{2-}**.

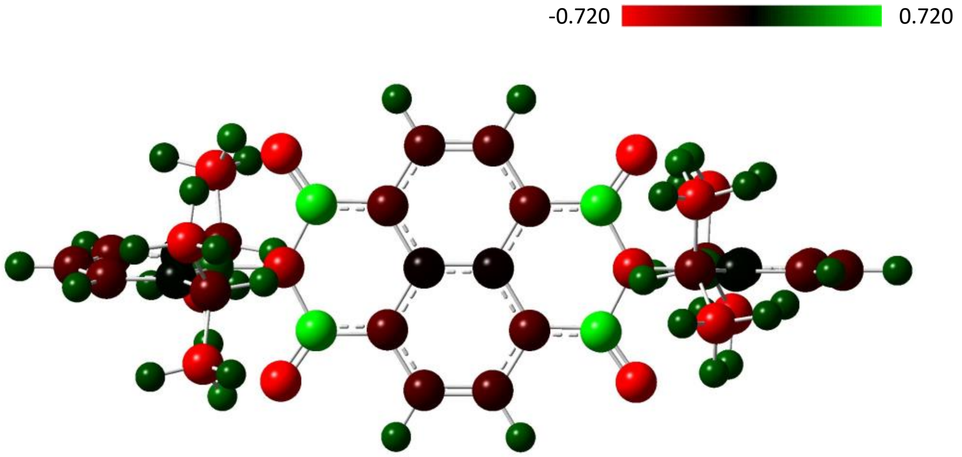

Figure S89. DFT-calculated structure and electron population density distribution of **NTCDA-di^{i}PrAn^{2-}**.

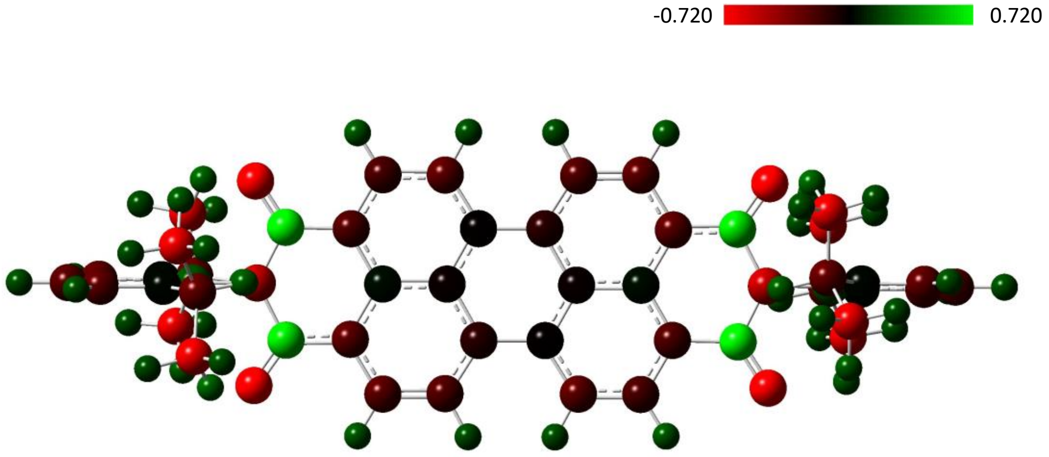

Figure S90. DFT-calculated structure and electron population density distribution of **PTCDA-di^{i}PrAn^{2-}**.

7. Tabulated calculated data for polymers and molecular analogues.

Table S12. Theoretical capacities, initial capacities, and capacity retention after 100 cycles for all studied polymers in this work.

Polymeric Material	Theoretical Capacity (mAh/g)	Initial Capacity[a] (mAh/g)	Capacity Retention[b] (%)	Rate Retention[c] (%)
**PMDA-pPDA**	184.7	**Li^+:** 33.5	**Li^+:** 53	**Li^+:** 17
		**Na^+:** 84.3	**Na^+:** 9	**Na^+:** 13
		**K^+:** 94.6	**K^+:** 13	**K^+:** 12
**NTCDA-pPDA**	157.5	**Li^+:** 49.1	**Li^+:** 81	**Li^+:** 60
		**Na^+:** 57.7	**Na^+:** 57	**Na^+:** 27
		**K^+:** 71.2	**K^+:** 81	**K^+:** 49
**PTCDA-pPDA**	115.4	**Li^+:** 75.9	**Li^+:** 67	**Li^+:** 80
		**Na^+:** 82.5	**Na^+:** 69	**Na^+:** 63
		**K^+:** 84.0	**K^+:** 74	**K^+:** 69
**PTCDA-chex**	113.9	**Li^+:** 83.5	**Li^+:** 47	**Li^+:** 62
		**Na^+:** 83.6	**Na^+:** 48	**Na^+:** 72
		**K^+:** 82.8	**K^+:** 74	**K^+:** 71
**PTCDA-en**	128.7	**Li^+:** 93.4	**Li^+:** 73	**Li^+:** 76
		**Na^+:** 100.9	**Na^+:** 76	**Na^+:** 80
		**K^+:** 141.3	**K^+:** 78	**K^+:** 84

[a]Initial capacity extracted from the third cycle at 100 mA g^{-1}.
[b]Capacity retention from cycle 3 to cycle 100 at 100 mA g^{-1}.
[c]Capacity retention from cycle 20 at 100 mA g^{-1} to cycle 48 at 10,000 mA g^{-1}.

The binding energies of the charge compensating ions to the small molecule analogues were estimated based on the observed reduction potentials relative to the TBA$^+$ binding energy. The potential shift relative to the reduction potential in the presence of TBA$^+$ (ΔE) was used to calculate the binding energy ($\Delta G_{\text{binding}}$) by the following equation:

$$\Delta G_{binding} = nF\Delta E \qquad \text{Eq. S4}$$

Table S13. Changes in the second binding energies ($\Delta G_{\text{binding}}$) of each charge balancing ion in comparison to the binding energy of TBA$^+$ calculated from Eq. S4.

	**PMDA-di^{i}PrAn**		**NTCDA-di^{i}PrAn**		**PTCDA-di^{i}PrAn**	
	E^0 (−1/−2) (V vs. SHE)	$\Delta G_{binding}$ (kJ/mol)	E^0 (−1/−2) (V vs. SHE)	$\Delta G_{binding}$ (kJ/mol)	E^0 (−1/−2) (V vs. SHE)	$\Delta G_{binding}$ (kJ/mol)
Li^+	−1.349	16.6	−1.003	6.5	−0.745	2.3
Na^+	−1.424	9.4	−1.049	2.1	−0.767	0.2
K^+	−1.467	5.3	−1.071	-0.1	−0.765	0.4
TBA^+	−1.522	0	−1.070	0	−0.769	0

8. References.

1) A. J. Greenlee, C. K. Ofosu, Q. Xiao, M. M. Modan, D. E. Janzen and D. D. Cao, *ACS Omega*, 2018, **3**, 240–245.
2) N. G. W. Cowper, C. P. Chernowsky, O. P. Williams and Z. K. Wickens, *J. Am. Chem. Soc.* 2020, **142**, 2093–2099.
3) L. H. Tong, P. Pengo, W. Clegg, J. P. Lowe, P. R. Raithby, J. K. M. Sanders and S. I. Pascu, *Dalton Trans.*, 2011, **40**, 10833–10842.
4) I. Ghosh, T. Ghosh, J. I. Bardagi and B. König, *Science*, 2014, **346**, 725–728.
5) M. J. Robb, B. Newton, B. P. Fors and C. J. Hawker, *J. Org. Chem.*, 2014, **79**, 6360–6365.
6) J. Seok, C. N. Gannett, S. H. Yu and H. D. Abruña, *Anal. Chem.*, 2021, **93**, 15459–15467.
7) J. Da Chai and M. Head-Gordon, *J. Chem. Phys.*, 2008, **128**, 084106.
8) M. J. Frisch, G. W. Trucks, H. B. Schlegel, G. E. Scuseria, M. a. Robb, J. R. Cheeseman, G. Scalmani, V. Barone, G. a. Petersson, H. Nakatsuji, X. Li, M. Caricato, a. V. Marenich, J. Bloino, B. G. Janesko, R. Gomperts, B. Mennucci, H. P. Hratchian, J. V. Ortiz, a. F. Izmaylov, J. L. Sonnenberg, Williams, F. Ding, F. Lipparini, F. Egidi, J. Goings, B. Peng, A. Petrone, T. Henderson, D. Ranasinghe, V. G. Zakrzewski, J. Gao, N. Rega, G. Zheng, W. Liang, M. Hada, M. Ehara, K. Toyota, R. Fukuda, J. Hasegawa, M. Ishida, T. Nakajima, Y. Honda, O. Kitao, H. Nakai, T. Vreven, K. Throssell, J. a. Montgomery Jr., J. E. Peralta, F. Ogliaro, M. J. Bearpark, J. J. Heyd, E. N. Brothers, K. N. Kudin, V. N. Staroverov, T. a. Keith, R. Kobayashi, J. Normand, K. Raghavachari, a. P. Rendell, J. C. Burant, S. S. Iyengar, J. Tomasi, M. Cossi, J. M. Millam, M. Klene, C. Adamo, R. Cammi, J. W. Ochterski, R. L. Martin, K. Morokuma, O. Farkas, J. B. Foresman and D. J. Fox, Gaussian 16, Revision C.01, 2016.
9) F. Weigend and R. Ahlrichs, *Phys. Chem. Chem. Phys.*, 2005, **7**, 3297–3305.
10) C. Y. Legault, CYLview20, Université de Sherbrooke, http://www.cylview.org.
